# Impact of reduced dose of ready-to-use therapeutic foods in children with uncomplicated severe acute malnutrition: A randomised non-inferiority trial in Burkina Faso

**DOI:** 10.1371/journal.pmed.1002887

**Published:** 2019-08-27

**Authors:** Suvi T. Kangas, Cécile Salpéteur, Victor Nikièma, Leisel Talley, Christian Ritz, Henrik Friis, André Briend, Pernille Kaestel

**Affiliations:** 1 Department of Nutrition, Exercise and Sports, University of Copenhagen, Copenhagen, Denmark; 2 Expertise and Advocacy Department, Action Against Hunger (ACF), Paris, France; 3 Nutrition and Health Department, Action Against Hunger (ACF) mission, Ouagadougou, Burkina Faso; 4 Centers for Disease Control and Prevention, Atlanta, United States of America; 5 Center for Child Health Research, University of Tampere School of Medicine, Tampere University, Tampere, Finland; London School of Hygiene and Tropical Medicine, UNITED KINGDOM

## Abstract

**Background:**

Children with uncomplicated severe acute malnutrition (SAM) are treated at home with ready-to-use therapeutic foods (RUTFs). The current RUTF dose is prescribed according to the weight of the child to fulfil 100% of their nutritional needs until discharge. However, there is doubt concerning the dose, as it seems to be shared, resulting in suboptimal cost-efficiency of SAM treatment. We investigated the efficacy of a reduced RUTF dose in community-based treatment of uncomplicated SAM.

**Methods and findings:**

We undertook a randomised trial testing the non-inferiority of weight gain velocity of children with SAM receiving (a) a standard RUTF dose for two weeks, followed by a reduced dose thereafter (reduced), compared with (b) a standard RUTF dose throughout the treatment (standard). A mean difference of 0.0 g/kg/day was expected, with a non-inferiority margin fixed at −0.5 g/kg/day. Linear and logistic mixed regression analyses were performed, with study site and team as random effects. Between October 2016 and July 2018, 801 children with uncomplicated SAM aged 6–59 months were enrolled from 10 community health centres in Burkina Faso. At admission, the mean age (± standard deviation [SD]) was 13.4 months (±8.7), 49% were male, and the mean weight was 6.2 kg (±1.3). The mean weight gain velocity from admission to discharge was 3.4 g/kg/day and did not differ between study arms (Δ 0.0 g/kg/day; 95% CI −0.4 to 0.4; *p* = 0.92) confirming non-inferiority (*p* = 0.013). However, after two weeks, the weight gain velocity was significantly lower in the reduced dose with a mean of 2.3 g/kg/day compared with 2.7 g/kg/day in the standard dose (Δ −0.4 g/kg/day; 95% CI −0.8 to −0.02; *p* = 0.041). The length of stay (LoS) was not different (*p* = 0.73) between groups with a median of 56 days (interquartile range [IQR] 35–91) in both arms. No differences were found between reduced and standard arm in recovery (52.7% and 55.4%; *p* = 0.45), referral (19.2% and 20.1%; *p* = 0.80), defaulter (12.2% and 8.5%; *p* = 0.088), non-response (12.7% and 12.5%; *p* = 0.95), and relapse (2.4% and 1.8%; *p* = 0.69) rates, respectively. However, the reduced RUTF dose had a small 0.2 mm/week (95% CI 0.04 to 0.4; *p* = 0.015) negative effect on height gain velocity with a mean height gain of 2.6 mm/week with reduced and 2.8 mm/week with standard RUTF dose. The impact was more pronounced in children under 12 months of age (interaction, *p* = 0.019) who gained 2.8 mm/week with reduced and 3.1 mm/week with standard dose (Δ −0.4 mm/week; 95% CI −0.6 to −0.2; *p* < 0.001). Limitations include not blinding participants to the RUTF dose received and excluding all children with negative appetite test. The results are generalisable for relatively food secure contexts with a young SAM population.

**Conclusions:**

Reducing the RUTF dose provided to children with SAM after two weeks of treatment did not reduce overall weight or mid-upper arm circumference (MUAC) gain velocity nor affect recovery or lengthen treatment time. However, it led to a small but significant negative effect on linear growth, especially among the youngest. The potential effect of reducing the RUTF dose in a routine program on treatment outcomes should be evaluated before scaling up.

**Trial registration:**

ISRCTN registry ISRCTN50039021.

## Abstract 2

Suvi Kangas and colleagues reveal a lowered dose of ready-to-use therapeutic food for malnourished children does not reduce weight gain, but can reduce linear growth.

## Author summary

**Why was this study done?:**

- Severe acute malnutrition (SAM) affects 19 million children worldwide and is treated with ready-to-use therapeutic foods (RUTFs).
- The current RUTF formulation and dosage are based on an inpatient treatment model and aim at achieving fast weight gain and recovery.
- However, when used in a home-based setting, the RUTF dose prescribed is often considered too large, resulting in sharing and a suboptimal cost-efficiency of SAM treatment.
- We sought to investigate the efficacy of reducing the RUTF dosage used in community-based treatment of uncomplicated SAM.

**What did the researchers do and find?:**

- We conducted a randomised controlled trial testing the non-inferiority of a reduced RUTF dose in the management of uncomplicated SAM compared with standard dose. This was called the MANGO trial.
- We enrolled 801 children 6–59 months of age and randomised them individually into receiving (1) a standard dose of RUTF throughout the treatment and (2) a standard dose during the first two weeks, followed by a reduced dose from third treatment week onwards.
- The trial showed non-inferior weight gain velocity from admission to discharge and similar recovery and length of stay in treatment.
- However, the reduced dose also resulted in a significantly slower height gain velocity compared with the standard dose.

**What do these findings mean?:**

- Our findings suggest that the reduction of the RUTF dose after the first two weeks results in similar weight gain velocity and recovery rates as with the standard dose given throughout SAM treatment.
- However, the reduced RUTF dose seems to slow down the height gain velocity of children and might thus not be fully optimal for children’s healthy growth.
- The reduced dose approach should be tested in a routine programmatic setting and in different food security contexts before scale-up.

## Introduction

Worldwide, 19 million children under 5 years of age suffer from severe acute malnutrition (SAM), contributing to over 500,000 deaths per year [1]. According to the World Health Organization (WHO) guidelines for community-based management of acute malnutrition (CMAM), children without medical complications at admission are treated as outpatients, with weekly checkup visits [2]. Treatment consists of a systematic antibiotic regimen, as well as a ready-to-use therapeutic food (RUTF), prescribed according to the weight of the child and continued until discharge [2].

RUTFs are highly fortified energy dense pastes that are designed to fulfil 100% of the nutritional needs of children during the recovery from SAM [3]. In theory, the prescribed dose should enable weight gains up to 20 g/kg/day, as observed in inpatient treatment of SAM with RUTFs [4]. However, high weight gain rates have never been observed in community settings where the average ranges between 1.0 and 5.5 g/kg/day using RUTFs [5–19], suggesting a lower intake of the therapeutic product in home-based treatment. Several studies have suspected or reported product sharing within and outside the household [8,13,20] as a reason for lower weight gain.

The perceived high cost and large quantity of RUTFs administered [20–25] have sparked attempts to optimise the product formulation and use [11,26–28]. One cluster-randomised trial in Sierra Leone gradually reduced the RUTF dose of children recovering from SAM when they reached moderate acute malnutrition (MAM) criteria [28]. However, the use of different recovery criteria between intervention and control groups limits the interpretation of the results. A retrospective analysis of a CMAM program in Myanmar, where, due to RUTF shortage, the dose was reduced once children with SAM reached MAM status, showed high recovery rates (90.2%). The lack of a control group in the study limits the interpretability of data [18]. To our knowledge, no rigorous clinical trial looking at the efficacy of reducing the RUTF dose among children with SAM has been conducted. Reducing the RUTF dose given to children treated for SAM in a cost restrained setting could enable the management of more malnourished children with the same resources.

The present study aimed to test, in a non-inferiority randomised controlled design, the impact of reducing the RUTF dose, after two weeks, on the weight gain velocity of children treated for uncomplicated SAM in the community. The reduction aimed to support weight gain rates of 5 g/kg/day and simplify the distribution and use of RUTF by children with SAM to 1 or 2 daily sachets for children <7 kg and ≥7 kg, respectively.

## Methods

### Ethics

The study was performed in accordance with the principles in the Declaration of Helsinki. The research protocol obtained ethical clearance from the national ethics committee (Comité d'éthique pour la recherche en santé [CERS]) and the clinical trials board (Direction Générale de la Pharmacie, du Médicament et des Laboratoires [DGPML]) of Burkina Faso. An independent Data Safety Monitoring Board composed of one paediatrician and one statistician was responsible for monitoring serious adverse events and conducted five complete data reviews during the course of the study. Caregivers provided verbal and written consent prior to enrolment and were made aware of their right to withdraw from the study at any time. Caregivers in both arms were given an instant photo of their child at the end of the treatment period and a bucket with soap at the end of the 3-month post-discharge follow-up period to compensate for the time spent on study procedures.

### Study design

We conducted a randomised controlled clinical trial (called MANGO) comparing the efficacy of a reduced RUTF dose to a standard RUTF dose in the management of uncomplicated SAM in children 6–59 months of age in a non-inferiority design.

### Study setting and participants

The study was conducted in the Fada N’Gourma health district located in the Eastern region of Burkina Faso. Malaria is endemic, with 69.3% of children presenting a positive rapid test [29]. HIV prevalence is 1.0% among 15–49-year-olds. In 2016, the prevalence of severe wasting (weight-for-height z-score [WHZ] <−3) and moderate wasting (WHZ between −3 and −2) was 2.4% and 8.6%, respectively [30]. There were 42 health centres in the district in 2015, all run by the Ministry of Health and supported by Action Against Hunger; 10 were chosen as study sites based on criteria on minimum SAM caseload (>7 new SAM admissions/month), accessibility, and a suitable schedule to couple study visit days with routine growth monitoring days.

Between October 2016 and July 2018, study participants were selected from children presenting with SAM at the 10 participating health centres for curative and preventive activities. Study staff checked admission criteria: WHZ <−3 and/or mid-upper arm circumference (MUAC) <115 mm, positive appetite test (performed as per the national protocol [31]), no oedema or medical complications, and between 6 and 59 months of age. Exclusion criteria included having received treatment for SAM within 6 months, caregiver planning to travel or unable to comply with the weekly checkup schedule, peanut or milk allergy, or disability affecting food intake. Children with any grade of oedema or medical complications, as defined by the Burkina national protocol for CMAM [31], at any time during the study were referred to inpatient care.

### Randomisation

Randomisation was stratified by health centre using varying block sizes from 2 to 8. Randomisation lists were generated using the website www.randomization.com. After confirming eligibility and obtaining consent from the caregiver, children were given a unique study identifier (ID) by a team supervisor and assigned to a treatment group. Only the RUTF distributors had access to the randomisation lists, while staff involved in assessing the eligibility and study outcomes of the child were blinded to the trial arm. Participants could not be blinded to the RUTF dose received. Investigators remained blinded to treatment groups until the final analysis stage.

### Study visits and procedures

Upon admission, the child’s caregiver was interviewed regarding household socioeconomic characteristics, care practices, and recent morbidity of the child and encouraged to adhere to weekly visits until recovery. Anthropometric measurements and a clinical examination were performed at each visit from admission to discharge. As per national SAM treatment protocol, seven key messages were delivered to caregivers in both groups, including advice to continue breastfeeding and to offer family foods in addition to RUTF if needed.

Anthropometrics were measured in duplicate at each visit: weight using an electronic scale (SECA 876, SECA, Hamburg, Germany) to the nearest 100 g, height (recumbent for <24 months of age; standing for ≥24 months of age) using a wooden measuring board (locally made) to the nearest 1 mm, and MUAC using a non-stretchable colourless measuring tape to the nearest 1 mm. Using WHO field tables, WHZ was determined and used for admission and discharge. In later analysis, WHZ was calculated using the package ‘zscore06’ [32] in STATA 15 (StataCorp, College Station, TX).

Children were followed up until recovery. Children missing their study visit were contacted either directly by telephone or via a community health worker and encouraged to return. Children referred did not return to trial after inpatient phase, as referral was considered a trial endpoint. Recovered children were followed up fortnightly for 12 weeks and relapses recorded. A supplementary feeding program accompanied the post-discharge follow-up, providing ready-to-use supplementary foods when available.

### Treatment protocol

Treatment followed the Burkina national CMAM guidelines in all aspects except the RUTF dose. Half of the children received a reduced dose from the third treatment week onwards (Table 1). Medical treatment included 7 days of amoxicillin for all children at admission (50–100 mg/kg/day), albendazole at the second treatment visit for children ≥12 months (200 mg to 12–23-month-olds; 400 mg to ≥24-month-olds) and catch-up doses for missed routine vaccinations or vitamin A supplementation (100,000 IU to 6–11-month-olds; 200,000 IU to 12–59-month-olds, every 6 months) at admission. Any illness, such as malaria, respiratory tract infections, or diarrhoea, diagnosed during the study was treated according to national protocol. See S2 Text for the full protocol for the study.

**Table 1:** RUTF dose in reduced and standard dose groups.

Weight (kg)	Sachets/week			Percent of reduction
	Standard RUTF dose	Reduced RUTF dose		Reduced RUTF dose
	Admission to discharge	Week 1–2	Week 3 to discharge	From week 1–2 to week 3
3.0–3.4	8	8	7	13%
3.5–4.9	10	10	7	30%
5.0–6.9	15	15	7	53%
7.0–9.9	20	20	14	30%
10.0–14.9	30	30	14	53%

Abbreviation: RUTF, ready-to-use therapeutic foods.

### Data collection and management

Two study teams were comprised of one nurse, three measurers, one food distributor, and one supervisor per team. All team members were trained on research ethics and processes; standard operating procedures were defined, tested, and applied. Data were collected via tablets using the Open Data Kit (ODK1 software), and continuous data monitoring and cleaning were performed by a data manager under the supervision of the principal investigator. Electronic data were password protected, and field registries were kept in a locked office. Data were de-identified prior to analysis.

### Outcomes

The primary outcome was weight gain velocity (g/kg/day) from admission to discharge. Other outcomes included weight gain velocity after two weeks, length of stay (LoS), discharge anthropometrics, linear and MUAC growth, treatment outcome, morbidity, and relapses.

Weight gain velocity from admission to discharge was calculated by dividing the weight gain (weight at discharge − weight at admission) in grams by the weight at admission in kilograms and the LoS in days. Weight gain velocity after two weeks was measured as follows: (weight at discharge − weight at visit 3 [in g]) ÷ (weight at admission [in kg]) ÷ (LoS − 14 [in days]). Missing weights at visit 3 (60 in reduced and 58 in standard arm) were imputed using mean weekly weight gained between an earlier visit (1 or 2) and later visit (4 or 5). The length of the stay was calculated as the number of days spent from admission to either recovery, referral, nonresponse, false discharge, or last visit before defaulting, lost to follow-up, or death. Linear and MUAC growth were defined as gains in millimetres (exit measure − admission measure)/week (LoS/7). A minimum acceptable mean rate of weight gain of 3.0 g/kg/day was defined at the protocol stage as a quality cutoff for evaluating general program performance.

Nutritional recovery was defined as reaching a WHZ of ≥−2 for those admitted with a WHZ <−3 only, or MUAC ≥125 mm for those admitted with a MUAC <115 mm only, or both WHZ ≥−2 and MUAC ≥125 mm for those admitted with both WHZ <−3 and MUAC <115 mm upon two consecutive visits and absence of any illness. Referrals included children referred to inpatient care as a result of medical complications, >5% weight loss within three weeks, or ≤100 g weight gain over four weeks in the absence of apparent illness. Nonresponse included children not reaching anthropometric discharge criteria by 16 weeks of treatment who were referred to inpatient care for further examinations. Defaulters were defined as having missed three consecutive visits, but the child was confirmed to be alive. Transfers to health centres not involved in the study were categorised as defaulters. ‘Lost to follow-up’ was defined as having missed three consecutive visits without a known status of the child. False discharges included children who were erroneously discharged as recovered or referred, but upon analysis did not meet the criteria. Relapses were recorded over 12 weeks following recovery and were defined as presenting a WHZ <−3 and/or a MUAC <115 mm, or any grade of bilateral oedema.

### Sample size

We assumed an expected mean difference in weight gain velocity between the two groups of 0.0 g/kg/day and a standard deviation (SD) of 2.6 g/kg/day with a non-inferiority margin of 0.5 g/kg/day. Assuming a power of 80% and a 5% significance level for a one-sided test, 335 children were needed in each group to demonstrate non-inferiority. To allow for dropout, the total target sample size was 800 children. Applying a 0.22 SD difference as could be observed in the main outcome with the calculated sample would allow us to detect a difference of 12% in recovery, seven days in LoS, 0.4 mm/week in MUAC gain velocity, and 0.3 mm/week in height gain velocity.

### Data analysis

Baseline characteristics of the study population were summarised as percentages and means (±SDs). Linear mixed models were used to compare primary and secondary outcomes of weight, linear and MUAC growth velocities, LoS, and anthropometric endpoints. Results were reported as differences of reduced dose from standard dose (reduced minus standard), with positive values meaning greater estimates among reduced dose. For programmatic outcomes, logistic mixed models were used to compare groups. Time to recovery was analysed using the Cox proportional hazards model. Study sites and research teams were included in the mixed models as random effects. Unadjusted models and models adjusted for sex, age, admission measure of weight, height, MUAC, WHZ, wealth, LoS in treatment, and month of admission were fitted. Adjustments were defined in the statistical analysis plan development stage prior to data analysis (S3 Text). Model checking was based on residual plots and normal probability plots, when applicable. All analyses were performed using STATA 15 (StataCorp).

Both intention to treat (ITT) and per protocol (PP) analyses were carried out for the main outcome and key secondary outcomes. ITT analysis included all children admitted to the study for whom an endpoint observation was available. PP analysis included children without missed visits who, according to maternal recall, consumed >50% of the daily dose at all times and excluded those who had received a wrong treatment dose or had been falsely discharged.

Interactions were only investigated in ITT analyses. Interactions between treatment and age group (<12 months versus ≥12 months), sex, MUAC category (<115 versus ≥115 mm), WHZ category (<−3 versus ≥−3), and stunting (height-for-age z-score [HAZ] < −2 versus HAZ ≥ −2) at admission were evaluated for the main outcome of weight gain velocity and the key secondary outcomes of recovery, LoS and height gain velocity, by means of likelihood ratio tests. Only significant interaction terms led to subgroup analyses.

‘Urban’ was defined as those living ≤30 minutes’ return trip from the regional capital city. Low birth weight (<2,500 g) was confirmed from an official birth certificate or health card. Household Food Insecurity Access Scale (HFIAS) was constructed according to FANTA indicator guide [33].

## Results

**Fig 1:**
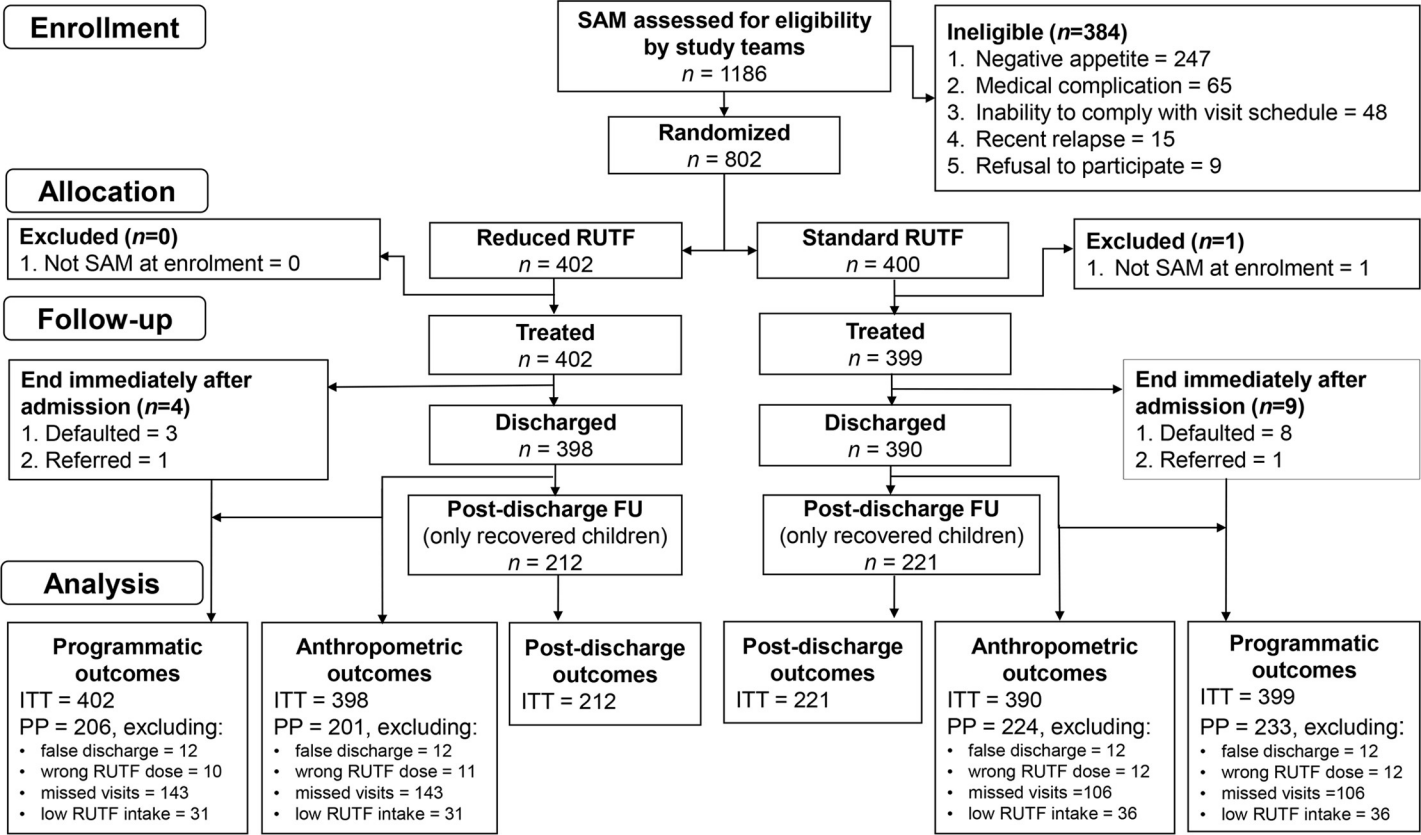
Patient flowchart. FU, follow-up; ITT, intention to treat; PP per protocol; RUTF, ready-to-use therapeutic food; SAM, severe acute malnutrition.

From October 17, 2016, to July 20, 2018, 1,186 children were diagnosed with SAM and assessed for eligibility at 10 study sites. Of these, 802 (68%) children were eligible for the study and randomised to standard or reduced RUTF dose (Fig 1). One child was excluded after randomisation for not meeting the SAM criteria at admission. Therefore, 801 patients were included in the trial: 402 in the reduced dose and 399 in the standard dose arm. Thirteen children defaulted or were referred immediately after admission; four and nine in reduced and standard dose arms, respectively. Three children developed oedema (two in reduced and one in standard dose arm) and were excluded from weight gain calculation. Only recovered children continued to the post-discharge follow-up, contributing to the post-discharge outcome analyses including relapse rate (Fig 1).

Randomisation resulted in baseline equivalence between the reduced and standard dose arms with respect to potential confounders (Table 2). The mean age at admission was 13.4 months, 49% were boys, and the mean admission weight was 6.2 kg. At visit 3, no children were <3.5 kg, 5% were 3.5–4.9 kg, 61% were 5.0–6.9 kg, 30% were 7.0–9.9 kg, and 4% were 10.0–14.9 kg. Caregivers were, on average, 28 years of age, 76% had no formal education, and 88% were categorised as food secure.

**Table 2:** Baseline characteristics of 801 children with SAM, randomised to reduced or standard RUTF dose.

Characteristic	*n*	Reduced RUTF	Standard RUTF
Age, months	801	13.3 ± 8.6	13.4 ± 8.9
Male, % (*n*)	801	49.5 (*199*)	49.4 (*197*)
Weight, kg	801	6.2 ± 1.2	6.2 ± 1.4
Height, cm	801	69.1 ± 7.4	69.1 ± 8.0
MUAC, mm	801	113 ± 7	113 ± 7
WHZ	801	−3.1 ± 0.7	−3.1 ± 0.7
HAZ	801	−2.4 ± 1.3	−2.4 ± 1.3
WAZ	801	−3.5 ± 0.8	−3.5 ± 0.8
Admission criteria, % (*n*)	801		
WHZ only		27 (*107*)	26 (*102*)
MUAC only		39 (*156*)	38 (*153*)
WHZ and MUAC		35 (*139*)	36 (*144*)
Low birth weight, % (*n*)	502	23 (*60*)	21 (*50*)
Urban, % (*n*)	801	15 (*59*)	14 (*54*)
Health centre ≤30-minute return trip, % (*n*)	801	39 (*155*)	37 (*149*)
Caregiver’s age	801	27.8 ± 7.6	27.6 ± 7.9
Mother has no formal education, % (*n*)	801	76 (*305*)	75 (*301*)
HFIAS category, % (*n*)	801		
Food secure		88 (*353*)	88 (*350*)
Mild food insecurity		9 (*35*)	9 (*34*)
Moderate or severe food insecurity		3 (*14*)	4 (*15*)
Open defecation, % (*n*)	801	77 (*311*)	75 (*298*)

All values are means ± SD unless otherwise indicated.

Abbreviations: HAZ, height-for-age z-score; HFIAS, Household Food Insecurity Access Scale; MUAC, mid-upper arm circumference; RUTF, ready-to-use therapeutic food; SAM, severe acute malnutrition; SD, standard deviation; WAZ, weight-for-age z-score; WHZ, weight-for-height z-score.

### Primary outcome

**Fig 2:**
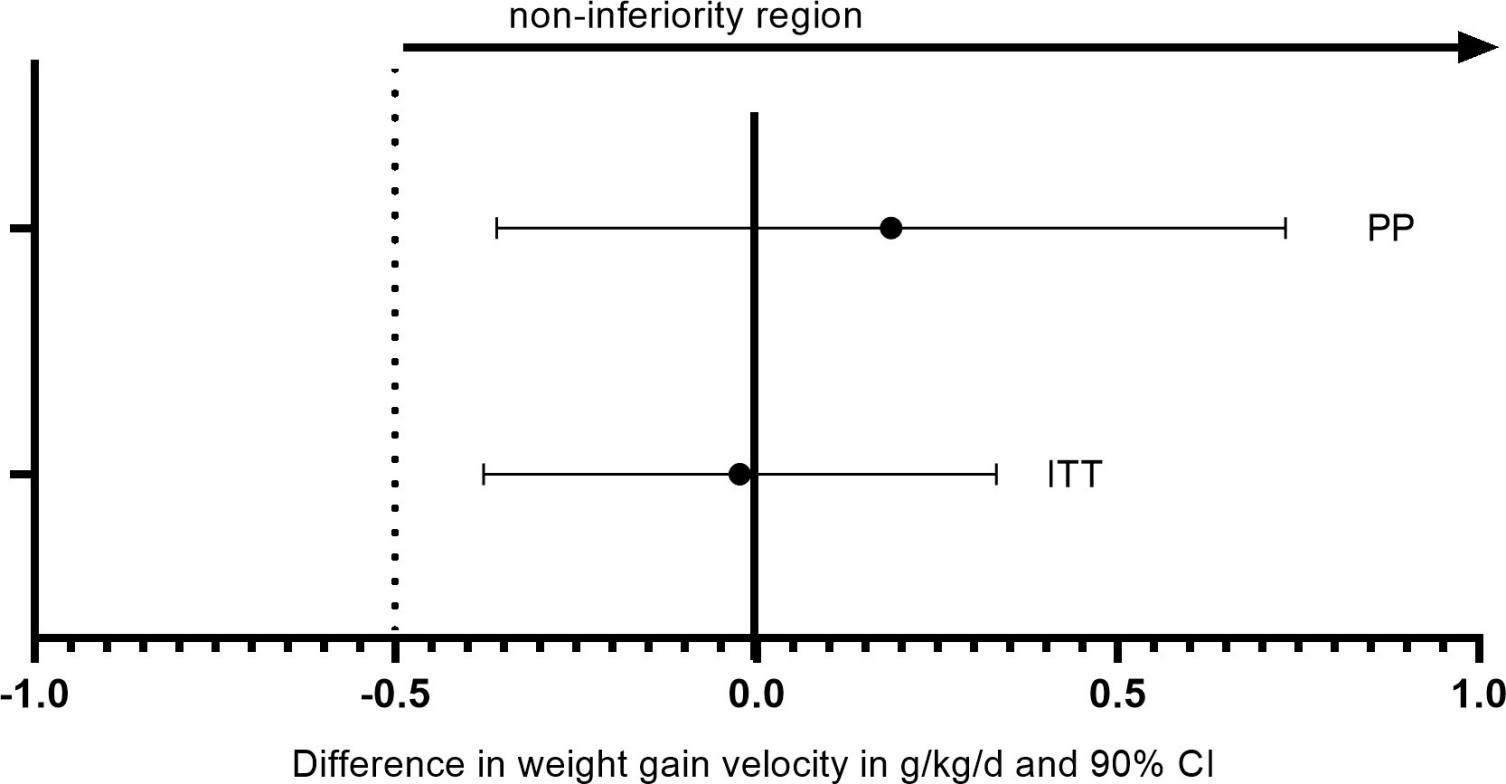
Difference in mean weight gain velocity (g/kg/day and 90% CI) in children with SAM randomised to reduced dose compared with standard dose in ITT and PP confirming non-inferiority. ITT, intention to treat; PP, per protocol; SAM, severe acute malnutrition.

**Fig 3:**
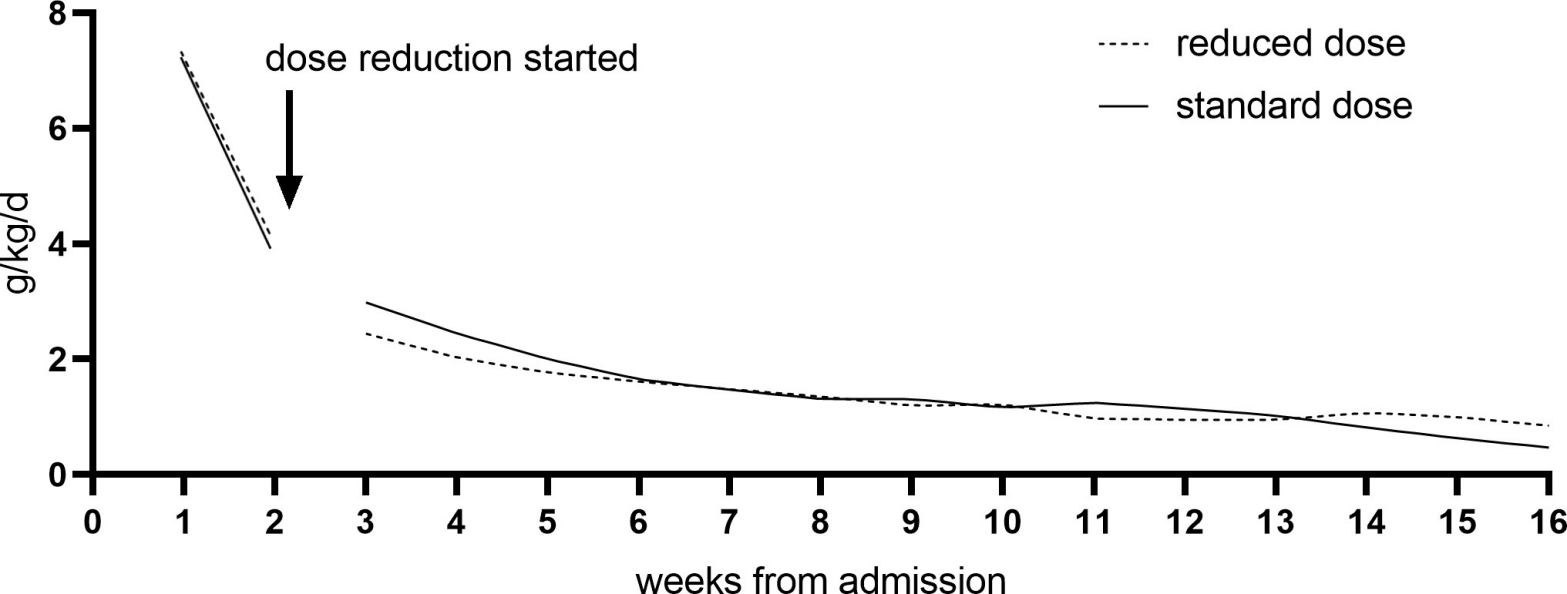
Weekly weight gain velocity (g/kg/day) of children with SAM randomised to reduced or standard RUTF dose, modeled using the mean weight of each group per visit. The first two weeks, when both groups were receiving the standard dose, were plotted apart in order not to mask any effect on weight gain after the reduction came into effect at week 3. A lowess curve was fitted with 224 points calculated between the weeks ranging from 3 to 16. RUTF, ready-to-use therapeutic food; SAM, severe acute malnutrition.

The mean weight gain velocity from admission to discharge was 3.4 g/kg/day in both groups in ITT analysis (Δ 0.0 g/kg/day; 95% CI −0.4 to 0.4). Non-inferiority of the reduced dose could be confirmed in both ITT (inferiority rejected: *p* = 0.013) and PP (inferiority rejected: *p* = 0.019) for this main outcome (Fig 2). No differences were found in weight gain velocity in PP analysis (Δ 0.2 g/kg/day; 95% CI −0.5 to 0.8), in ITT among recovered only (Δ −0.1 g/kg/day; 95% CI −0.6 to 0.4), referrals (Δ 0.5 g/kg/day; 95% CI −0.6 to 1.5), or defaulters (Δ −0.3 g/kg/day; 95% CI −1.3 to 0.8) (Table 3). No interactions were found between treatment and sex, age, MUAC category, WHZ category, or stunting status at admission. In general, mean weight gain velocity was high at the start of the treatment and decreased rapidly (Fig 3). When entering third treatment week, 27% of children still had SAM (WHZ <−3 and/or MUAC <115 mm): 108 children in the reduced and 106 children in the standard group.

**Table 3:** Weight and MUAC gain velocity of children with SAM randomised to reduced or standard RUTF dose in unadjusted model.

Outcome	Reduced RUTF		Standard RUTF		Difference (95% CI)	*p*-value
				
Admission to Discharge	*n*	mean ± SD	*n*	mean ± SD		
**Weight gain velocity (g/kg/day)**						
ITT	396	3.4 ± 3.1	389	3.4 ± 3.1	0.0 (−0.4 to 0.4)	0.92
PP^1^	200	4.3 ± 3.4	223	4.1 ± 3.6	0.2 (−0.5 to 0.8)	0.58
Recovered	212	4.9 ± 2.6	221	4.9 ± 2.5	−0.1 (−0.6 to 0.4)	0.73
Referred	74	0.9 ± 3.4	78	0.4 ± 3.0	0.5 (−0.6 to 1.5)	0.37
Defaulted	46	2.5 ± 1.9	26	2.7 ± 2.7	−0.3 (−1.3 to 0.8)	0.64
**MUAC gain velocity (mm/week)**						
ITT	398	1.8 ± 1.8	390	1.9 ± 1.9	−0.1 (−0.3 to 0.2)	0.58
PP^1^	201	2.4 ± 2.1	224	2.4 ± 2.1	−0.1 (−0.5 to 0.3)	0.78
**After Two Weeks**						
**Weight gain velocity (g/kg/day)**						
ITT	376	2.3 ± 2.6	368	2.7 ± 2.9	−0.4 (−0.8 to −0.02)	0.041
PP^1^	188	2.7 ± 2.9	207	3.1 ± 3.4	−0.4 (−1.0 to 0.2)	0.22
**MUAC gain velocity (mm/week)**						
ITT	378	1.1 ± 1.7	368	1.4 ± 1.9	−0.2 (−0.5 to −0.001)	0.051
PP^1^	189	1.6 ± 1.9	207	1.8 ± 2.2	−0.2 (−0.6 to 0.2)	0.27

Data are mean ± SD and mean difference (95% CI) when using linear mixed models, with study site and team as random effects.

^1^PP (per protocol) includes children that had no missed visits, that consumed >50% of daily dose throughout treatment, that were not falsely discharged, and that received the correct RUTF dose throughout treatment.

Abbreviations: ITT, intention to treat; MUAC, mid-upper arm circumference; PP, per protocol; RUTF, ready-to-use therapeutic food; SAM, severe acute malnutrition.

Weight gain velocity after the first two weeks of treatment (in ITT) was significantly different between groups, with a mean of 2.3 g/kg/day with reduced versus 2.7 g/kg/day with standard dose (Δ −0.4 g/kg/day; 95% CI −0.8 to −0.02). Results comparing the MUAC gain velocity between reduced and standard doses mirrored the results obtained with weight gain velocity (Table 3). Adjusted analysis yielded similar results that are found in S1 Table.

### Secondary outcomes

No differences were found in anthropometry at discharge between study arms in the unadjusted model (all *p* > 0.2). However, when using the adjusted model (adjusting for sex, age, admission measure of weight, MUAC, WHZ and height, month of admission, LoS, and wealth index), height at discharge was significantly smaller in the reduced dose arm (Table 4). This difference in height of 0.1 cm could still be observed 3 months post-recovery, although the difference was then no longer significant (*p* = 0.33). Weight, MUAC, weight-for-age z-score (WAZ), WHZ, and HAZ were not different at three months post-recovery.

**Table 4:** Anthropometry at discharge of children with SAM randomised to reduced or standard RUTF dose with difference (95% CI) in unadjusted and adjusted ITT analysis.

Outcome	n	Reduced RUTF	Standard RUTF	Unadjusted		Adjusted*	
				Difference (95% CI)	*p*-value	Difference (95% CI)	*p*-value
Weight, kg	785	7.3 ± 1.4	7.3 ± 1.5	−0.0 (−0.2 to 0.2)	0.74	−0.0 (−0.1 to 0.03)	0.25
Height, cm	788	71.5 ± 7.0	71.6 ± 7.5	−0.1 (−1.1 to 0.9)	0.87	−0.1 (−0.2 to −0.03)	0.013
MUAC, mm	788	125.2 ± 8.1	125.9 ± 8.5	−0.7 (−1.9 to 0.4)	0.21	−0.7 (−1.7 to 0.3)	0.17
WHZ	784	−2.0 ± 0.9	−1.9 ± 1.0	−0.0 (−0.2 to 0.1)	0.64	−0.0 (−0.2 to 0.1)	0.63
HAZ	788	−2.3 ± 1.2	−2.3 ± 1.3	0.0 (−0.2 to 0.2)	0.93	−0.0 (−0.1 to 0.1)	0.60
WAZ	785	−2.7 ± 0.9	−2.6 ± 1.0	−0.0 (−0.1 to 0.1)	0.82	−0.0 (−0.1 to 0.1)	0.51

Data are mean ± SD and mean difference (95% CI) when using linear mixed models with study site and team as random effects.

*Adjusted for sex, age, admission measure of weight, MUAC, WHZ and height, month of admission, LoS, and wealth index.

Abbreviations: HAZ, height-for-age z-score; ITT, intention to treat; LoS, length of stay; MUAC, mid-upper arm circumference; RUTF, ready-to-use therapeutic food; SD, standard deviation; WAZ, weight-for-age z-score; WHZ, weight-for-height z-score.

**Fig 4:**
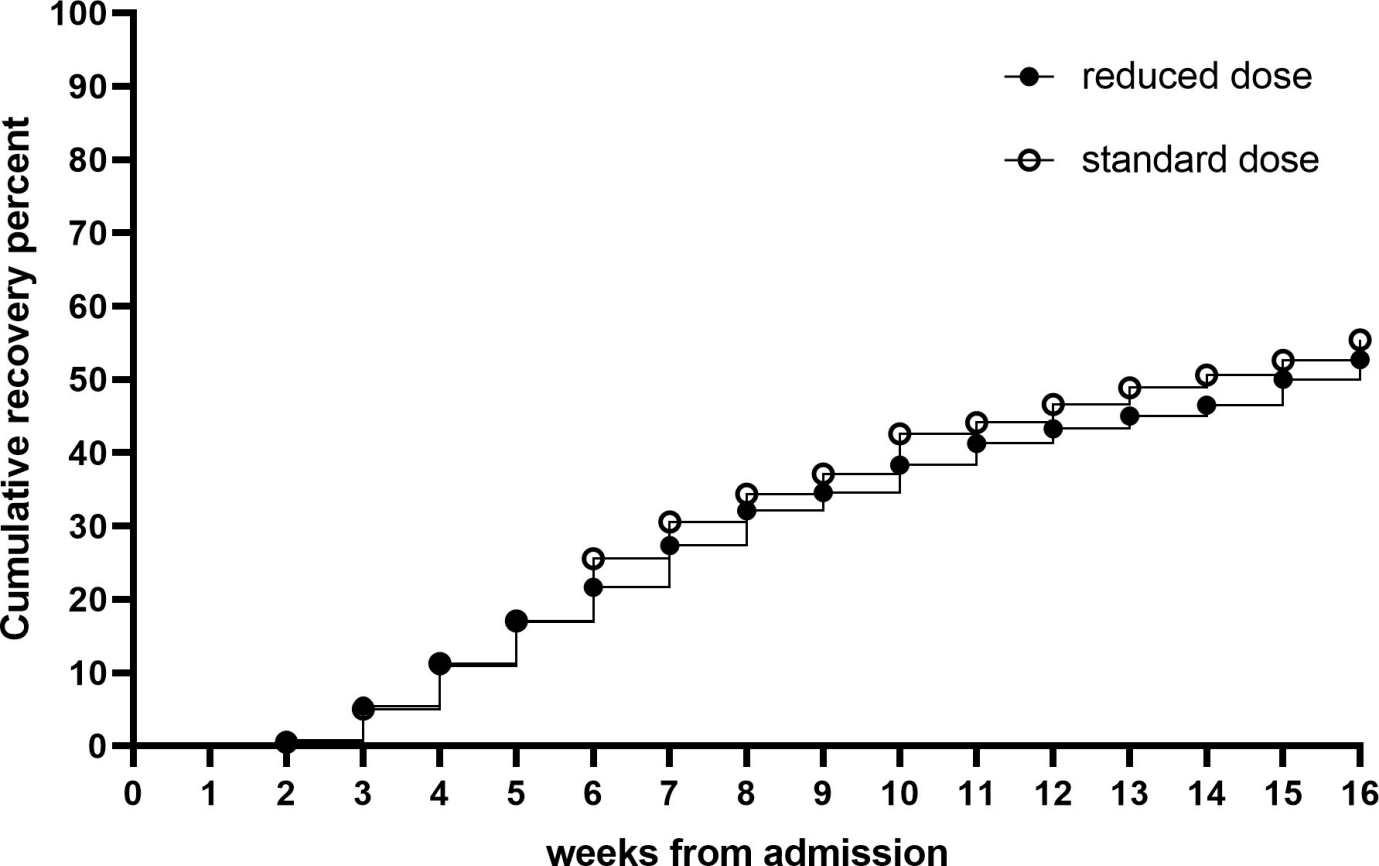
Weekly recovery among children with SAM randomised to reduced or standard RUTF dose. RUTF, ready-to-use therapeutic food; SAM, severe acute malnutrition.

The median LoS was 56 days (interquartile range [IQR] 35–91) in both arms. The WHZ category was an effect modifier (interaction, *p* = 0.028) whereby children who were admitted with a WHZ ≥−3 and treated with the reduced dose had a LoS 6.9 days (95%CI −0.1 to 13.9; *p* = 0.055) longer than those treated with the standard dose. No difference was found in the LoS of children admitted with WHZ <−3 between reduced and standard doses. No effect modification for LoS was observed between treatment and age, sex, MUAC category, or stunting at admission. The recovery rate was similar in both arms: 52.7% in reduced dose and 55.4% in standard dose (Δ −2.6%; 95% CI −9.5 to 4.3) (Table 5). Cox proportional hazards model showed no difference (*p* = 0.54) in the time to recovery between the two arms (Fig 4). No significant interactions were found for recovery. No differences were found in the proportion of children referred (19.2% and 20.1%), defaulting (12.2% and 8.5%), nonresponding (12.7% and 12.5%), and relapsed (2.4% and 1.8%) between reduced and standard RUTF dose arms, respectively (Table 5). PP analysis provided similar results and is found in S2 Table. The number or duration of illnesses between study arms did not differ (*p* > 0.2): 60% of children had a respiratory illness, 38% had malaria, and 52% had diarrhoea at some point after admission. During the intervention, two children died (one in each group), one was lost to follow-up (standard dose), and 24 were falsely discharged (12 in each group).

**Table 5:** Programmatic outcomes of children with SAM randomised to reduced or standard RUTF dose with risk difference (95% CI) in unadjusted ITT analysis.

Outcome	Reduced RUTF		Standard RUTF		Difference (95% CI)	*p*-value
	*n*	median [IQR]	*n*	median [IQR]		
LoS, days	402	56 [35–91]	399	56 [35–91]	0.8 (−3.7 to 5.3)	0.73
Subgroup analysis by						
WHZ at admission	402		399			0.028*
<−3	238	56 [35–84]	238	56 [35–98]	−3.3 (−9.1 to 2.5)	0.26
≥−3	164	56 [35–91]	161	49 [35–77]	6.9 (−0.1 to 13.9)	0.055
		**% (*n*)**		**% (*n*)**		
Recovery	402	52.7 (*212*)	399	55.4 (*221*)	−2.6 (−9.5 to 4.3)	0.45
Referral	402	19.2 (*77*)	399	20.1 (*79*)	−0.7 (−6.2 to 4.8)	0.80
Weight loss	402	12.9 (*52*)	399	15.3 (*61*)	−2.4 (−7.2 to 2.5)	0.34
Stagnant weight	402	4.0 (*16*)	399	3.8 (*15*)	0.0 (−2.6 to 3.0)	0.88
Medical complication	402	2.2 (*9*)	399	1.0 (*4*)	1.2 (−0.5 to 2.8)	0.18
Defaulter	402	12.2 (*49*)	399	8.5 (*34*)	3.7 (−0.5 to 7.9)	0.088
Lost to follow-up	402	0.0 (*0*)	399	0.3 (*1*)	NA	
Nonresponse	402	12.7 (*51*)	399	12.5 (*50*)	−0.2 (−4.4 to 4.8)	0.95
Died	402	0.3 (*1*)	399	0.3 (*1*)	NA	
False discharge	402	3.0 (*12*)	399	3.0 (*12*)	−0.0 (−2.4 to 2.3)	0.99
Relapse	212	2.4 (*5*)	221	1.8 (*4*)	0.5 (−2.1 to 3.2)	0.69

Data are median [IQR] for LoS and percentage (*n*) for other outcomes and mean difference (95% CI) for the differences. Linear mixed models were used, with study site and team as random effects.

*p for interaction. Interactions were tested in ITT for sex, age, MUAC category, WHZ category, and stunting status at admission, and only significant terms (*p* < 0.05) and subsequent subgroups analysis are reported.

Abbreviations: IQR, interquartile range; ITT, intention to treat; LoS, length of stay; MUAC, mid-upper arm circumference; RUTF, ready-to-use therapeutic food; WHZ, weight-for-height z-score.

Height gain velocity was lower in children who received the reduced dose (2.6 mm/week) than the standard dose (2.8 mm/week) (Δ −0.2 mm/week; 95% CI −0.4 to −0.04). Age was an effect modifier (interaction, *p* = 0.019): the height gain of children under 12 months of age was 2.8 mm/week with reduced dose and 3.1 mm/week with standard dose (Δ −0.4 mm/week; 95% CI −0.6 to −0.2). Similarly, from admission to discharge, HAZ increased by 0.05 SD with reduced and 0.09 SD with standard RUTF dose (Δ −0.04 SD; 95% CI −0.09 to 0.002). Again, age was an effect modifier (interaction, *p* = 0.016): children under 12 months of age had 0.00 SD catch-up in HAZ with reduced dose compared with 0.09 SD with standard dose (Δ −0.09 SD; 95% CI −0.15 to −0.03) (Table 6). Height gain or HAZ catch-up did not differ between RUTF dose among children ≥12 months. Adjusted analysis provided similar results that are found in S3 Table.

**Table 6:** Height gain velocity (mm/week) and HAZ change (SD) from admission to discharge of children with SAM randomised to reduced or standard RUTF dose and difference (95% CI) in unadjusted model.

Outcome	Reduced RUTF		Standard RUTF		Difference (95% CI)	*p*-value
	*n*	mean ± SD	*n*	mean ± SD		
**Height gain velocity (mm/week)**						
ITT	398	2.6 ± 1.3	390	2.8 ± 1.3	−0.2 (−0.4 to −0.04)	0.015
PP^1^	201	2.5 ± 1.3	224	2.9 ± 1.4	−0.3 (−0.6 to −0.1)	0.009
Subgroup analysis by						
Admission age	398		390			0.019*
<12 months	244	2.8 ± 1.2	235	3.1 ± 1.1	−0.4 (−0.6 to −0.2)	<0.001
≥12 months	154	2.3 ± 1.3	155	2.2 ± 1.2	0.03 (−0.2 to 0.3)	0.85
**HAZ change (SD)**						
ITT	398	0.05 ± 0.35	390	0.09 ± 0.32	−0.04 (−0.09 to 0.002)	0.063
PP^1^	201	0.06 ± 0.31	224	0.10 ± 0.27	−0.04 (−0.10 to 0.01)	0.12
Subgroup analysis by						
Admission age	398		390			0.016*
<12 months	244	0.00 ± 0.39	235	0.09 ± 0.35	−0.09 (−0.15 to −0.03)	0.003
≥12 months	154	0.13 ± 0.25	155	0.10 ± 0.25	0.03 (−0.05 to 0.10)	0.47

Data are shown as mean ± SD and mean difference (95% CI) using linear mixed models, with study site and research team as random effects.

*p for interaction. Interactions were tested in ITT for sex, age, MUAC category, WHZ category, and stunting status at admission, and only significant terms (*p* < 0.05) and subsequent subgroups analysis are reported.

^1^PP (per protocol) includes children that had no missed visits, that consumed >50% of the daily dose throughout treatment, that were not falsely discharged, and that received the correct RUTF dose throughout treatment.

Abbreviations: HAZ, height-for-age z-score; ITT, intention to treat; MUAC, mid-upper arm circumference; PP, per protocol; RUTF, ready-to-use therapeutic food; SD, standard deviation; WHZ, weight-for-height z-score.

## Discussion

Evidence is needed to inform policy on the optimisation of treatment of uncomplicated SAM. The current trial investigated the efficacy of reducing the RUTF dose after two weeks and showed that there was no effect on the total weight or MUAC gain velocity, recovery, or LoS in treatment. However, the linear growth of children receiving the reduced dose was significantly slower, particularly among the youngest group of <12-month-old children.

The mean weight gain velocity of 3.4 g/kg/day observed from admission to discharge is in line with those reported in earlier CMAM studies [5–20]. While non-inferiority was confirmed for weight gain velocity from admission to discharge, the difference in weight gain after two weeks was 0.4 g/kg/day (*p* = 0.041) between children receiving the reduced dose of RUTF (mean 2.3 g/kg/day) compared with the standard dose (mean 2.7 g/kg/day). This finding suggests that the rapid weight gain in the first two weeks, when all children received the standard dose, masks the small negative effect the reduced dose has on the subsequent growth of children. The weight gain velocity during treatment shows a quickly decreasing pattern, in which the first weeks represent high catch-up in weight. From the fifth treatment week onward, the weight gain velocity drops to <2 g/kg/day and resembles that observed in MAM treatment programs [34–36]. From the eighth week onward, the weight gain velocity was approximately 1 g/kg/day, similar to normal weight gain velocity for a healthy one-year-old child [37]; this despite continuing to receive RUTF. However, recovery continues throughout treatment until the 16th week, when those not yet recovered were considered ‘nonresponse’ to treatment.

Linear growth observed in the trial (2.6 mm/week with reduced dose and 2.8 mm/week with standard dose) is in line with other CMAM studies [5,6,9,10,16,19,38] and with the 2.8 mm/week growth rates expected in healthy 13-month-old children [39]. However, the reduced dose slowed down the linear growth of children by 0.2 mm/week (95% CI 0.04 to 0.4; *p* = 0.015). Whether this difference is clinically significant remains questionable. In general, linear growth is considered at least as important, if not more important, than weight gain for the healthy growth of children [40]. A follow-up study of children recovered from SAM found that seven years later, these children had similar weight- and BMI-for-age, but significantly lower HAZ and absolute height compared with their siblings and community controls [41]. HAZ and absolute height are both predictors of chronic disease in later life [42]. In our trial, the mean HAZ was −2.4 at admission, and it increased by 0.05 and 0.09 with the reduced and standard doses, respectively (Δ −0.04 HAZ; 95% CI −0.09 to 0.002; *p* = 0.063). On the contrary, among children treated for MAM, HAZ decreased by 0.17 during 12 weeks of supplementary feeding in Burkina Faso [34]. Reasons for different linear catch-up growth between children with MAM and SAM could include therapeutic food quality and quantity. Linear growth requires micronutrients, in particular, type 2 micronutrients such as zinc, magnesium, and potassium [43], which are provided by RUTF [3] at much higher levels than are available in local diets [44]. A reduction in the RUTF dose or quality possibly reduces the quantity and density of these nutrients and by consequence may affect linear growth.

The observed recovery rates (52.7% with reduced dose and 55.4% with standard dose) are low but are explained by the strict application of referral criteria in our trial, and that referral was considered an effective study endpoint. When using the SPHERE calculation method [45] excluding referred and false discharge, recovery rates were 68% in the reduced dose and 72% in standard dose, somewhat under the recommended >75%. Up to 20% of children were referred primarily as a result of weight loss or stagnant weight, which may not often be identified in routine programs. Nevertheless, the similar referral rate between study arms suggests these referrals are not related to a dose effect. In a post hoc analysis, weight loss was associated with higher numbers and longer duration of illness episodes. Episodes of infection are known to drive undernutrition via appetite loss, reduced nutrient absorption, nutrient losses, diversion of nutrients to inflammatory responses, and tissue repair [46]. Open defecation was practised by 76% of households, indicating a poorly sanitised home environment with high risk of exposure to pathogens [47]. This could partially explain a proportion of illness episodes and the relatively high proportion of cases with weight loss or stagnant weight. Beyond acute illness, environmental enteric dysfunction is another potential driver of suboptimal recovery [46].

In the current trial, the reduced RUTF dose had no negative effects on children ≥12 months of age but slowed down the height gain of children <12 months of age. Bahwere and colleagues (2016) found that providing milk-free RUTF had no adverse effect among children with SAM ≥24 months of age, while children <24 months of age had a significantly lower recovery rate. Seemingly, younger children are somewhat more sensitive to changes in RUTF quantity and quality, possibly requiring standard treatment in order to gain the full benefit from SAM treatment. Whether separate protocols should be considered for different age groups remains a question requiring operational feasibility, effectiveness, and cost estimations.

In the context of the current global transition from a focus on purely ensuring the survival of children to actually enabling them to thrive, the quest is emerging in the malnutrition community to go beyond recovery and seek to optimise the functional and long-term outcomes of children treated for SAM [41,48]. This will require looking at body composition, micronutrient status, cognitive development, and other long-term outcomes. At the same time, the resources are limited and cost-efficiency should be taken into account and existing and new investments evaluated against the benefits they can bring.

The main strength of the study is the individually randomised design with few dropouts immediately after admission, which reduces confounding and enables a causal analysis of the effect of the reduced RUTF dose on the weight gain of uncomplicated SAM. The field study was implemented by well-trained and experienced research staff who were responsible for the diagnosis, treatment, and discharge of children with SAM. The number of patients per day was also limited to enable thorough care and follow-up of the study children.

As with all studies, there are limitations. First, although we did not reveal the study arm to participants, it was not possible to blind them to the RUTF dose received. However, because the daily dose prescribed to children in both arms depended on the weight of the child, we did not expect caregivers to be fully aware of the treatment allocation. With the exception of the RUTF distributor, the research staff were blinded to the dose. Second, we excluded and referred to inpatient care all children who did not pass the appetite test at admission. While officially a referral criterion, the appetite test is not always implemented in the field. It is possible that children who in routine practice fail the appetite test have a slower weight gain at the beginning of treatment and thereafter an inferior response to a reduced dose after two weeks.

The study findings are only generalisable to a nonemergency context with relatively good food security and where SAM cases are primarily very young (<24 months of age). However, further research is needed to corroborate these findings in a routine program with fewer resources and time per patient, and to translate these finding to an emergency context or to older-aged SAM cases.

In conclusion, reducing the RUTF dose prescribed to children with SAM after two weeks does not appear to affect the total weight or MUAC gain velocity, recovery rate, nor the LoS in treatment. However, the linear growth of children became slower with the RUTF reduction, especially in young children. Before considering a reduction of RUTF during SAM treatment, an effectiveness study in a routine program setting is needed to confirm the results.

## Supporting information

S1 Table Results of the adjusted analysis of weight and MUAC gain velocity. MUAC, mid-upper arm circumference.
(DOCX)

S2 Table Results of the PP analysis of programmatic outcomes. PP, per protocol.
(DOCX)

S3 Table Results of the adjusted analysis of height and HAZ gain. HAZ, height-for-age z-score.
(DOCX)

S1 Text CONSORT checklist. (DOC)

S2 Text MANGO study protocol. (DOCX)

S3 Text MANGO statistical analysis plan. (DOCX)

S1 Abstract French language translation of the Abstract. (DOCX)

## Abbreviations

CERS: Comité d'éthique pour la recherche en santé
CMAM: community-based management of acute malnutrition
DGPML: Direction Générale de la Pharmacie, du Médicament et des Laboratoires
HAZ: height-for-age z-score
HFIAS: Household Food Insecurity Access Scale
ID: identifier
IQR: interquartile range
ITT: intention to treat
LoS: length of stay
MAM: moderate acute malnutrition
MUAC: mid-upper arm circumference
PP: per protocol
RUTF: ready-to-use therapeutic food
SAM: severe acute malnutrition
SD: standard deviation
WAZ: weight-for-age z-score
WHO: World Health Organization
WHZ: weight-for-height z-score
