# Supplementary material for: Impact of reduced dose of ready-to-use therapeutic foods in children with uncomplicated severe acute malnutrition: A randomised non-inferiority trial in Burkina Faso
Source: PLoS Med. 2019 Aug 27;16(8):e1002887. doi: 10.1371/journal.pmed.1002887 (PMC6711495; doi:10.1371/journal.pmed.1002887)
Supplement: S1 Table — MUAC, mid-upper arm circumference. (DOCX) [file pmed.1002887.s001.docx]

S1 Table: Weight and MUAC gain velocity of children with SAM randomised to reduced or standard RUTF dose and difference when adjusted for sex, age, admission measure of weight, MUAC, WHZ and height, month of admission, length of stay and wealth index.

| **Outcome** | n | Reduced  RUTF | Standard RUTF | Difference  (95% CI) | *p* value |
| --- | --- | --- | --- | --- | --- |
| **ADMISSION TO DISCHARGE** | | | | | |
| **Weight gain velocity (g/kg/d)** |  |  |  |  |  |
| Intention to treat | 785 | 3.4 ± 3.1 | 3.4 ± 3.1 | -0.02 (-0.4; 0.4) | 0.93 |
| Per protocol^1^ | 423 | 4.3 ± 3.4 | 4.1 ± 3.6 | 0.1 (-0.5; 0.7) | 0.76 |
| Recovered only | 433 | 4.9 ± 2.6 | 4.9 ± 2.5 | 0.01 (-0.3; 0.3) | 0.97 |
| Referred | 155 | 0.9 ± 3.4 | 0.4 ± 3.0 | 0.4 (-0.5; 1.3) | 0.42 |
| Defaulted | 73 | 2.5 ± 1.9 | 2.7 ± 2.7 | -0.2 (-1.3; 0.8) | 0.67 |
| **MUAC gain velocity (mm/w)** |  |  |  |  |  |
| Intention to treat | 788 | 1.8 ± 1.8 | 1.9 ± 1.9 | -0.1 (-0.3; 0.1) | 0.51 |
| Per protocol | 425 | 2.4 ± 2.1 | 2.4 ± 2.1 | -0.1 (-0.4; 0.3) | 0.76 |
| **AFTER FIRST 2 WEEKS** | | | | | |
| **Weight gain velocity (g/kg/d)** |  |  |  |  |  |
| Intention to treat | 744 | 2.3 ± 2.6 | 2.7 ± 2.9 | -0.4 (-0.8; -0.01) | 0.044 |
| Per protocol^1^ | 395 | 2.7 ± 2.9 | 3.1 ± 3.4 | -0.4 (-1.0; 0.2) | 0.22 |
| **MUAC gain velocity (mm/w)** |  |  |  |  |  |
| Intention to treat | 746 | 1.1 ± 1.7 | 1.4 ± 1.9 | -0.2 (-0.5; -0.01) | 0.041 |
| Per protocol^1^ | 396 | 1.6 ± 1.9 | 1.8 ± 2.2 | -0.3 (-0.6; 0.1) | 0.18 |
| Data are mean ± SD and mean difference (95% CI) when using linear mixed models with study site and team as random effects and when adjusted for sex, age, admission measure of weight, MUAC, WHZ and height, month of admission, length of stay and wealth index.  ^1^ Per protocol: includes children that had no missed visits, that consumed > 50% of daily dose throughout treatment, that were not falsely discharged and that received the correct RUTF dose throughout treatment. MUAC, mid-upper arm circumference; RUTF, ready-to-use therapeutic food. | | | | | |
