# Supplementary material for: Impact of reduced dose of ready-to-use therapeutic foods in children with uncomplicated severe acute malnutrition: A randomised non-inferiority trial in Burkina Faso
Source: PLoS Med. 2019 Aug 27;16(8):e1002887. doi: 10.1371/journal.pmed.1002887 (PMC6711495; doi:10.1371/journal.pmed.1002887)
Supplement: S2 Table — PP, per protocol. (DOCX) [file pmed.1002887.s002.docx]

S2 Table: Programmatic outcomes of children with SAM randomised to reduced or standard RUTF dose with risk difference (95% CI) in per protocol analysis

| **Per protocol^1^** | n | Reduced  RUTF | Standard RUTF | Difference  (95% CI) | *p* value |
| --- | --- | --- | --- | --- | --- |
| Length of stay, days | 425 | 42 [28; 70] | 42 [28; 63] | 1 (-5; 6) | 0.78 |
| Subgroup analysis by |  |  |  |  |  |
| WHZ at admission |  |  |  |  | 0.73* |
| <-3 |  | 49 [28; 70] | 42 [28; 70] | -0.4 (-7.4; 6.6) | 0.91 |
| ≥-3 |  | 42 [28; 56] | 42 [28;56] | 1.5 (-6.7; 9.7) | 0.72 |
| Recovery | 425 | 67.7 (*136*) | 64.7 (*145*) | -2.8 (-6.2; 11.9) | 0.54 |
| Referral | 425 | 16.9 (*34*) | 23.2 (*52*) | -6.2 (-14.0; 1.6) | 0.12 |
| Weight loss | 425 | 12.4 (*25*) | 17.0 (*38*) | -4.5 (-11.2; 2.2) | 0.19 |
| Stagnant weight | 425 | 3.5 (*7*) | 4.9 (*11*) | -1.5 (-5.5; 2.5) | 0.47 |
| Medical complication | 425 | 1.0 (*2*) | 1.3 (*3*) | -0.3 (-2.4; 1.7) | 0.74 |
| Defaulter | 425 | 9.5 (*19*) | 5.8 (*13*) | 3.6 (-1.4; 8.7) | 0.16 |
| Lost to follow-up | 425 | 0 | 0 | NA |  |
| Non-response | 425 | 5.5 (*11*) | 5.8 (*13*) | -0.3 (-4.7; 4.1) | 0.88 |
| Died | 425 | 0.5 (*1*) | 0.5 (*1*) | 0.1 (-1.3; 1.3) | 0.94 |
| Relapse | 281 | 2.2 (*3*) | 1.4 (*2*) | 0.8 (-2.3; 3.9) | 0.60 |
| Data are median [IQR] for length of stay and percentage (*n*) for other outcomes and mean difference (95% CI) for the differences. Linear mixed models were used with study site and team as random effects.  **^1^** Per protocol: includes children that had no missed visits, that consumed > 50% of daily dose throughout treatment, that were not falsely discharged and that received the correct RUTF dose throughout treatment.  **p* for interaction.  RUTF, ready-to-use therapeutic food; WHZ, weight-for-height z-score. | | | | | |
