# Supplementary material for: Impact of reduced dose of ready-to-use therapeutic foods in children with uncomplicated severe acute malnutrition: A randomised non-inferiority trial in Burkina Faso
Source: PLoS Med. 2019 Aug 27;16(8):e1002887. doi: 10.1371/journal.pmed.1002887 (PMC6711495; doi:10.1371/journal.pmed.1002887)
Supplement: S3 Table — HAZ, height-for-age z-score. (DOCX) [file pmed.1002887.s003.docx]

S3 Table. Height gain velocity (mm/w) and HAZ change (SD) from admission to discharge of children with SAM randomised to reduced or standard RUTF dose and difference (95% CI) when adjusting for sex, age, admission measure of weight, MUAC, WHZ and height, month of admission, length of stay and wealth index.

| **Outcome** | n | Reduced RUTF | Standard RUTF | Difference  (95% CI) | *p* value |
| --- | --- | --- | --- | --- | --- |
| **Height gain velocity (mm/w)** |  |  |  |  |  |
| Intention to treat | 788 | 2.6 ± 1.2 | 2.8 ± 1.2 | -0.2 (-0.4; -0.1) | 0.010 |
| Per protocol^1^ | 425 | 2.5 ± 1.4 | 2.8 ± 1.5 | -0.3 (-0.5; -0.02) | 0.036 |
| Subgroup analysis by |  |  |  |  |  |
| Admission age |  |  |  |  | 0.057* |
| <12 months | 479 | 2.8 ± 1.2 | 3.1 ± 1.1 | -0.3 (-0.5; -0.1) | 0.001 |
| ≥12 months | 309 | 2.3 ± 1.3 | 2.3 ± 1.2 | -0.02 (-0.3; 0.2) | 0.88 |
| **HAZ change (SD)** |  |  |  |  |  |
| Intention to treat | 788 | 0.05 ± 0.35 | 0.09 ± 0.32 | -0.04 (-0.08; 0.00) | 0.053 |
| Per protocol^1^ | 425 | 0.06 ± 0.31 | 0.10 ± 0.27 | -0.04 (-0.09; 0.02) | 0.18 |
| Subgroup analysis by |  |  |  |  |  |
| Admission age |  |  |  |  | 0.11* |
| <12 months | 479 | 0.00 ± 0.39 | 0.09 ± 0.35 | -0.07 (-0.12; -0.01) | 0.014 |
| ≥12 months | 309 | 0.13 ± 0.25 | 0.10 ± 0.25 | 0.00 (-0.06; 0.07) | 0.95 |
| Data are mean ± SD and mean difference (95% CI) using linear mixed models with study site and team as random effects and when adjusted for sex, age, admission measure of weight, MUAC, WHZ and height, month of admission, length of stay and wealth index.  ^1^ Per protocol: includes children that had no missed visits, that consumed > 50% of daily dose throughout treatment, that were not falsely discharged and that received the correct RUTF dose throughout treatment.  **p* for interaction.  HAZ, height-for-age z-score; RUTF, ready-to-use therapeutic food. | | | | | |
