# Supplementary material for: Impact of reduced dose of ready-to-use therapeutic foods in children with uncomplicated severe acute malnutrition: A randomised non-inferiority trial in Burkina Faso
Source: PLoS Med. 2019 Aug 27;16(8):e1002887. doi: 10.1371/journal.pmed.1002887 (PMC6711495; doi:10.1371/journal.pmed.1002887)
Supplement: S2 Text — (DOCX) [file pmed.1002887.s005.docx]

**MANGO**

**Modelling an Alternative Nutrition Protocol Generalizable for Outpatient**

Efficacy of an optimized dosage of RUTF for the treatment of Severe Acute Malnutrition: A randomized controlled, non-inferiority trial in Burkina Faso

| **Version** | **Date** | **Update concerning** | **Clarification to update** |
| --- | --- | --- | --- |
| 1 | 15/8/2015 |  |  |
| 2 | 10/7/2016 | **Added**: Body composition measurement for all children included in the trial and a cohort of normal children to act as reference | The measure was added to increase scientific value of the study at to be able to study the physiological response to treatment of SAM children.  A cohort of normal children were also measured in order to obtain context adapted comparison data. |
|  |  | **Dropped out** : Knee-heel length measure | It was judged there was no need nor time for the measure. |
|  |  | **Dropped out**: use of anaesthetic cream before blood sampling | After testing various creams in the field with un-satisfying effect, it was decided to leave out the use of an anaesthetic cream. This would also speed up the procedure and thus benefit the patients who don’t need to wait for the cream to act. |
|  |  | **Revised**: Referral criteria for stagnant weight | In order to align with national protocol, the stagnant weight definition was re-defined to mean a maximum of 100g of weight gain over the course of 4 weeks (instead of 0g in 3 weeks). |
|  |  | **Revised:** post-discharge follow-up | To align with the national protocol, the post-discharge follow-up period was reduced to 3 months (instead of 4) and the visits were increased to fortnightly instead of once a month. |

# Overview

| **TITLE** | **MANGO - Modelling an Alternative Nutrition Protocol Generalizable for Outpatient**  **Efficacy of an optimized dosage of RUTF for the treatment of Severe Acute Malnutrition: A randomized controlled, non-inferiority trial in Burkina Faso** |
| --- | --- |
| DATE | July 2016 |
| STUDY TYPE | Randomised controlled trial |
| FUNDING SOURCES | CIFF, ECHO, HIF small grant, ACF |
| PRINCIPAL INVESTIGATOR | Suvi Kangas, ACF |
| CO-PRINCIPAL INVESTIGATOR | Cécile Salpéteur, ACF |
| COLLABORATORS | André Briend, University of Copenhagen  Henrik Friis, University of Copenhagen  Carlos Navarro Colorado, CDC  Jonathan Wells, UCL |
| PARTNER INSTITUTIONS | Action contre la faim (ACF), Paris, France  Action contre la faim (ACF), Burkina Faso  University of Copenhagen, Denmark  Center for Diseases Control & Prevention, Atlanta, USA  University College of London Institute of Child Health, UK |
| DURATION | 2014-2020 |

# List of abbreviations

ACF Action Contre la Faim – France

AGP α_1-_acid glycoprotein

APP Acute phase protein

CMAM Community-based Management of Acute Malnutrition

CRF Case report form

CRP C-reactive protein

DSMB Data and Safety Monitoring Board

FGD Focus group discussion

HB Haemoglobin

LMIC Low and Middle Income Countries

MAM Moderate Acute Malnutrition

MUAC Mid upper arm circumference

RBP Retinol binding protein

RUSF Ready-to-use Supplementary Food

RUTF Ready-to-Use Therapeutic Food

s-TfR soluble transferrin receptors

SAM Severe Acute Malnutrition

SFP Supplementary feeding Program

WHO World Health organization

WHZ Weight-for-height Z-score

# Summary

**Background**

Severe acute malnutrition (SAM) affects around 19 million children worldwide as estimated by Lancet in 2013 (1). Without proper treatment the case-fatality rate is high (up to 50 %), but with recent development of treatment protocols mortality from SAM can be reduced to less than five percent (2). The majority of cases present with uncomplicated forms of SAM which can be treated at home with lipid based ready-to-use-therapeutic foods (RUTF) distributed through programs for community based management of acute malnutrition (CMAM). However, even though CMAM programs are effective in terms of recovery from SAM, the average weight gains observed in most CMAM programs remain far below the weight gains obtained with RUTF in inpatient settings. It is assumed that selling and sharing of RUTF are the main reasons behind the lower weight gains. To prevent misuse of RUTF for other population groups through sharing or selling, and to increase program effectiveness through minimized program costs, the MANGO project will use a reduced ration of RUTF. The reduced ration is based on the estimated RUTF consumed to obtain the mean weight gain achieved in routine CMAM programs.

**Objectives and outcomes**

The general objective is to assess the efficacy and cost of an optimized dosage scheme of RUTF to children aged 6 to 59 months with uncomplicated severe acute malnutrition. The primary objective is to assess the effect of the optimized ration of RUTF on **rate of weight gain** (g/kg/d) until recovery or other treatment outcome. Secondary objective is to assess the effect of the optimized ration on **duration of treatment**, i.e. number of days until recovery. Other outcomes include **cost-effectiveness** (average and incremental costs per child recovered) of the CMAM program, change in **vitamin A and iron status** and change in body composition of children treated for SAM.

**Interventions**

The standard RUTF sachet of 92 g (500 kcal) will be used for all participants, and the ration will depend on the weight of the child. However, the number of sachets given per day will differ between participants in the two groups after the scheme outlined below.

|  | Normal dose–RUTF | | Reduced dose - RUTF | | | |
| --- | --- | --- | --- | --- | --- | --- |
|  | From admission to discharge | | Weeks 1 and 2 | | From week 2 onwards until discharge | |
| Weight (kg) | Sachets/week | Kcal/kg/d | Sachets/week | Kcal/kg/d | Sachets/week | Kcal/kg/d |
| 3.0-3.4 | 8 | 168-190 | 8 | 168-190 | 7 | 147-167 |
| 3.5-4.9 | 10 | 183-204 | 10 | 183-204 | 7 | 102-143 |
| 5.0-6.9 | 15 | 155-214 | 15 | 155-214 | 7 | 72-100 |
| 7.0-9.9 | 20 | 144-204 | 20 | 144-204 | 14 | 101-143 |
| 10.0-14.9 | 30 | 144-214 | 30 | 144-214 | 14 | 67-100 |

**Study design**

The study is a randomized controlled non-inferiority trial using individual randomisation to standard or reduced ration of RUTF. Treatment will continue with weekly distribution of RUTF according to the intervention group until discharge. Discharge criteria are 1) WHZ ≥ -2 for children admitted to the program with WHZ < -3, 2) MUAC ≥ 125 mm for children admitted to the program with MUAC < 115mm or 3) WHZ ≥ -2 and MUAC ≥ 125 mm for children admitted to the program with WHZ < -3 and MUAC < 115 mm, at two consecutive visits. After discharge, and as per the Burkina national protocol, children will be enrolled into a supplementary feeding program (SFP) for 3 months. The supplementary feeding product used currently is a lipid based Ready-to-use supplementary food (RUSF), Plumpy’Sup. Children will be followed biweekly during this time to assess their relapse rate (See **Figure)**.


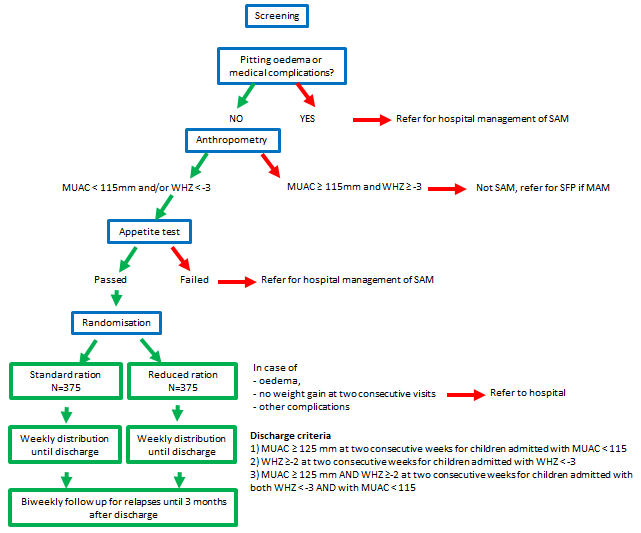


**FIGURE Study design**

**Methods**

***Anthropometry,*** i.e. rate of weight gain, change in mid-upper-arm circumference (MUAC), change in weight-for-height, and change in length-for-age will be measured using standard anthropometric equipment. All measurements will be done in duplicate using standardised methods and calibrated equipment.

***Cost-effectiveness*** of the CMAM program applying the standard and the optimized ration will be calculated as total program cost per child recovered, taking in consideration weekly ration as well as average time to recovery, defaulter rate etc.

***Blood samples*** will be taken from all participants at admission and discharge to measure vitamin A status and iron status. In addition, the ***body composition*** of the children will be measured using a bioimpedance method at admission and at discharge.

**Participants**

A total of 800 children aged 6-59 months admitted to outpatient treatment for uncomplicated severe acute malnutrition, defined as WHZ < -3 and/or MUAC < 115 mm without oedema or other complications requiring inpatient treatment. Participants will be recruited from 10 health centres, and randomisation will be stratified by health centre.

**Timing of events**

|  | Admission | CMAM treatment | | | Follow up for  3 mo after DC |
| --- | --- | --- | --- | --- | --- |
|  | Arrival | Weekly until discharge | | Discharge (DC) | Monthly for  four months after DC |
| Screening for eligibility | x |  |  | |  |
| Information, consent and randomisation | x |  |  | |  |
| Questionnaire on sociodemographic and household conditions | x |  |  | |  |
| Morbidity questionnaire | x | x | x | | x |
| Clinical assessment | x | x | x | | x |
| Anthropometry | x | x | x | | x |
| Blood sampling | x |  | x | |  |
| Distribution of RUTF | x | x |  | |  |
| Supplement adherence and acceptability |  | x | x | |  |
| Dietary intake |  | wk 4 |  | |  |

**Data management**

All data will be collected electronically via tablets using the Open Data Kit (ODK) software. At the end of each day, all data collected will be transferred to a central computer for treatment and storage. A data manager shall be responsible of the cleaning and quality control of the data and making regular safeguards. A Data Safety and Monitoring Board will be put in place and shall look over the data on agreed time points to evaluate the safety of the trial. Once all data has been collected it will be consolidated in one final database after which the data can be analysed. All analysis will be done using the *intention to treat* hypothesis.

**Ethical considerations**

Ethical approval will be sought from Burkina’s national Ethical Committee and the French Committee for People Protection. Upon enrolment potential participants will be explained the purpose of the project and the study procedures. The voluntary nature of participation will be emphasized. A letter of information will be shared with those that agree to participate and they shall be informed of the possibility to drop out at any time during the trial may they wish to do so.

**Table of Contents**

[Overview ii](#_Toc1461616)

[List of abbreviations 1](#_Toc1461617)

[Summary 2](#_Toc1461618)

[1. Introduction 1](#_Toc1461619)

[1.1. Background 1](#_Toc1461620)

[1.2. The context of the study area 3](#_Toc1461621)

[1.3. Treatment dosage 3](#_Toc1461622)

[1.4. Rationale 4](#_Toc1461623)

[1.5. Outcome measures 8](#_Toc1461624)

[2. Objectives 9](#_Toc1461625)

[2.1. General objective 9](#_Toc1461626)

[2.2. Specific objectives 9](#_Toc1461627)

[3. Methods 10](#_Toc1461631)

[3.1. Study design 10](#_Toc1461632)

[3.2. Blinding 11](#_Toc1461633)

[3.3. Randomization and blinding 12](#_Toc1461634)

[3.4. Study population 12](#_Toc1461635)

[3.4.1. Inclusion criteria 12](#_Toc1461636)

[3.4.2. Exclusion criteria 13](#_Toc1461637)

[3.5. Consent, inclusion and follow up of participants 13](#_Toc1461638)

[3.6. Recovery 14](#_Toc1461639)

[3.7. Defaulters and lost to follow-up 14](#_Toc1461640)

[3.8. Referrals 15](#_Toc1461641)

[3.9. Sample size 15](#_Toc1461642)

[3.10. Pilot study and training 16](#_Toc1461643)

[3.11. Questionnaires 17](#_Toc1461644)

[3.12. Dietary intake 17](#_Toc1461645)

[3.13. Anthropometry 18](#_Toc1461646)

[3.14. Body composition 19](#_Toc1461647)

[3.15. Clinical assessment and search for oedema 19](#_Toc1461648)

[3.16. Blood samples and analyses 19](#_Toc1461649)

[3.17. Qualitative study on RUTF sharing practices 20](#_Toc1461650)

[4. Data and Safety Monitoring Board (DSMB) 21](#_Toc1461651)

[5. Data management and analysis 21](#_Toc1461652)

[5.1. Data management 21](#_Toc1461653)

[5.2. Data analysis 22](#_Toc1461654)

[6. Risks and assumptions 23](#_Toc1461655)

[6.1. Funding and resources 23](#_Toc1461656)

[6.2. Community awareness and local cooperation 23](#_Toc1461657)

[6.3. Recruitment rate and study population 24](#_Toc1461658)

[7. Ethical considerations 24](#_Toc1461659)

[7.1. Autonomy (Informed consent) 24](#_Toc1461660)

[7.2. Treatment of complications and of patients not enrolled 25](#_Toc1461661)

[7.3. Confidentiality 25](#_Toc1461662)

[7.4. Risks (do not harm) 25](#_Toc1461663)

[7.5. Benefits to participants and community 26](#_Toc1461664)

[8. Dissemination of results 27](#_Toc1461665)

[9. Collaborating partners and conflicts of interest 27](#_Toc1461666)

[10. Management and financing 28](#_Toc1461667)

[11. References 30](#_Toc1461668)

[12. List of annexes 34](#_Toc1461669)

[12.1. Annex 1 Information to participants 35](#_Toc1461670)

[12.2. Annex 2 Consent form 39](#_Toc1461671)

[12.3. Annex 3 Chronogram 40](#_Toc1461672)

[12.4. Annex 4 Field team composition 41](#_Toc1461673)

[12.5. Annex 5 Project governance 43](#_Toc1461674)

[12.6. Annex 6 Signatures 45](#_Toc1461675)

[12.7. Annex 7 Optidiag study 46](#_Toc1461676)

#

#

# Introduction

## Background

Worldwide, it has been estimated that 52 million children under 5 are suffering from acute malnutrition, of which 19 million have severe acute malnutrition (SAM) (1). Acute malnutrition can manifest over a short period of time when the body does not receive adequate amounts of energy or other nutrients, either as a result of insufficient dietary intake or through malabsorption of nutrients and anorexia due to illness. Acute malnutrition increases the risk of infection through suppressing immunity (3), and is recognized as a direct and underlying cause of child mortality (1,4,5). Acute malnutrition also has negative implications on morbidity, long-term growth, cognitive and behavioural development, and work capacity amongst survivors (6,7).

SAM is defined as a weight-for-height less than 3 standard deviations below the median of the growth standard (WHZ < -3), or a mid-upper arm circumference (MUAC) less than 115 mm or the presence of bilateral oedema (8). Current protocols for the treatment of uncomplicated SAM recommend ambulatory care through the distribution of Ready-to-Use Therapeutic Food (RUTF) and standardized medical treatment (9).

Until the development of RUTF late 1990’s, children with SAM were treated with liquid therapeutic diets (F75/F100) in therapeutic feeding units as inpatients (10). However, hospital based treatment is associated with high risk of cross contamination from other sick individuals, high opportunity costs for caregivers, and high demand for skilled health professionals (2). With appropriate treatment diets and continuous follow up, SAM can be managed at home in the absence of life threatening complications. The lipid based therapeutic diet, the RUTF, has a low water activity. Bacteria do not grow in it if accidentally contaminated, and RUTF is therefore safe for community-based management of acute malnutrition (CMAM) (9).

However, even though CMAM programs are effective in terms of recovery from SAM, the average weight gains observed in most CMAM programs remain far below the weight gains obtained with RUTF in inpatient settings. One of the first studies of RUTF replacing the liquid treatment diet F-100 during the recovery phase of nutritional rehabilitation obtained a rate of weight gain of 15 g/kg/day providing 3 daily meals of RUTF ad libitum in addition to 3 daily meals prepared from local ingredients (11). The study was conducted in an inpatient setting in Senegal and the average total daily energy intake was 193 kcal/kg/d. Studies assessing the effectiveness of RUTF in CMAM settings have reported varying rates of weight gains despite the provision of equal RUTF rations (175kcal/kg /day) with rates of weight gain between less than 3.5g/kg/day and 12g/kg/day, depending on the country and context (12). Although limited information is available on the cause for variation in weight gain and response to treatment, it is assumed that selling and sharing of RUTF are the main reasons (13). As mentioned, despite the low rate of weight gain, the percentage of children recovering from SAM in CMAM programs remains high (median recovery rate 84.0%) (14) and within the Sphere standards (15).

The Coverage Monitoring Network has recently identified breaks in RUTF supply as one of the top 4 barriers influencing access and having a significant impact on attendance to CMAM programs (16). One key challenge in RUTF supply relates to the volume of RUTF as it is bulky and difficult to transport. Moreover, local governments in the countries affected by acute malnutrition remain dependent on external support. In 73% of countries with CMAM programs, UNICEF provides 100% of RUTF. The reliance of governments on RUTF donated by UNICEF or the Clinton Foundation is obviously a challenge to the sustainability of the approach at national level (17). To guarantee sustainability of CMAM, there is a need to shift responsibilities from UNICEF to the local government. However, the high cost of the product (up to 50% of overall CMAM cost (17)) prevents local governments from becoming the direct purchasers and responsible for the RUTF supply chain.

Depending on operational constraints, several adaptations to the international protocol have been implemented in the treatment of SAM. Médecins Sans Frontières (MSF) provided 2 sachets (1000kcal) of RUTF per day to 328 children 6 mo-5 years of age undergoing home-based treatment for uncomplicated SAM in Niger (18). The majority of the children (n=319, 90.1%) were between 12 and 35 months of age and the treatment resulted in a median ± SD weight gain of 9.8 ± 4.6 g/kg/day and an average length of stay of 29 ± 13.5 days (18). Caregivers were advised to provide family meals and hence RUTF served as a complement to the daily food ration. ACF implemented a low-dose RUTF protocol in Myanmar whereby RUTF was dosed according to beneficiary’s bodyweight, until the child reached a WHZ of ≥-3 and MUAC ≥110mm. From this point, the child received a fixed quantity of 1 sachet (500kcal) of RUTF per day, independent of bodyweight until discharge. A median [IQR] length of stay of 42 days [28; 56] and weight gain of 4.0 g/kg/day [3.0; 5.7] was reported and indicated that a lower RUTF dose, combined with specific measures to ensure good service quality and beneficiary support, was successful in treating uncomplicated SAM in this setting (19).

The above mentioned studies are examples of special situations with limited resources for a short duration of time, and have demonstrated that reduced dosages of RUTF may not weaken programme performance under such conditions. The MANGO research project aims to test, in a randomised design, the efficacy of a reduced dosage of RUTF in the context of CMAM. The underlying assumption is that the standard ration generates a surplus when administered at home in the context of other family members and household diets. Children are not consuming the entire ration and/or caregivers are giving family foods in addition to the RUTF. Unless caregivers are sharing a fixed proportion of the amount, irrespective of the amount given, it is expected that children who are given the reduced ration will receive an equal amount of RUTF as children given the standard ration, but less RUTF is available for sharing and/ or selling. If this assumption is true, the average weight gain will not differ between the two groups.

## The context of the study area

This study will be conducted in Fada N’Gourma health district, which is located in the eastern region of Burkina Faso. The district has a surface of 11,200 km² and a population of 391,282 inhabitants of which 21% are children below five years of age (20). Food access relies on the annual crop production. In addition, traditional beliefs, local practices and tradition remain barriers to prevent malnutrition (ACF, Burkina Faso Nutritional Causal Analysis report, 2013).

The district is characterized by a high crude birth rate (51.7‰), which contributes to a relatively high population growth rate (3.5%) (21). General mortality (11.8‰), infant mortality (91.8‰) and infant and child mortality (142.6‰) remain high in the district (21). In 2013, the prevalence (95% confidence interval) of global acute malnutrition (GAM), defined as WHZ <-2, in eastern region was estimated at 9.3% (8.3-10.4) and for severe acute malnutrition (SAM), defined as WHZ<-3, at 1.7% (1.3-2.3) (22). The prevalence of GAM and SAM in Fada N’Gourma health district seemed slightly higher with 10.2% (7.6-13.5) and 2.4% (1.4-4.0), respectively. This is classified as a medium to high level of acute malnutrition, requiring public health action (23).

The health district of Fada N’Gourma counts 42 public health centres providing outpatient care and one inpatient facility. Besides public health facilities, the district counts 12 private and 7 private-public health facilities. All health centres provide a minimum package of nutrition activities and enrol children identified with uncomplicated SAM in CMAM programs. Coverage of SAM treatment was estimated at 48% in 2014 (ACF SQUEAC survey 2014).

ACF's intervention in the district has since 2012 facilitated access to proper free SAM treatment while reinforcing the health system capacity and autonomy together with promotion of better practices within communities. The activities undertaken by ACF in 2015 and beyond are the following:

- *Prevention*: promotion of better food diversity through health gardens, infant enriched flour consumption and behaviour change through sensitization and care practices;
- *Support to detection, referral and treatment of SAM cases*: based on ACF's July 2014 health system diagnosis, focus will be made on the quality of service and ownership through health staff behaviour change promotion, capacity building, as well as management and storage of supplies
- *Improved access* to water and sanitation in health facilities.

Finally, ACF is continuously advocating for a better inclusion of malnutrition treatment within the health system.

## Treatment dosage

Analysis of response to treatment for SAM in children indicates that weight gain is higher during the initial weeks of treatment and slows down into a stable weight gain afterwards. At this stage there is no robust explanation but the compliance to treatment and the degree of undernutrition could play an important role.

All children will receive the standard RUTF (Plumpy’nut®) as is used in routine CMAM programs i.e. rounded up number of sachets of 500 kcal each (9) , but different dosages will be given (Table 1):

- Control group will receive standard RUTF ration as outlined in the national protocol (see **Table 1**)
- Intervention group will receive the low MANGO RUTF ration.

Table 1 RUTF dosage table for the two groups, in number of sachets per week and kcal/kg/day

|  | Normal dose - RUTF | | Reduced dose - RUTF | | | |
| --- | --- | --- | --- | --- | --- | --- |
|  | Admission to discharge | | Week1-2 | | Week 3 to discharge | |
| Weight (kg) | Sachets/wk | Kcal/kg/d | Sachets/wk | Kcal/kg/d | Sachets/wk | Kcal/kg/d |
| 3.0-3.4 | 8 | 168-190 | 8 | 168-190 | 7 | 147-167 |
| 3.5-4.9 | 10 | 183-204 | 10 | 183-204 | 7 | 102-143 |
| 5.0-6.9 | 15 | 155-214 | 15 | 155-214 | 7 | 72-100 |
| 7.0-9.9 | 20 | 144-204 | 20 | 144-204 | 14 | 101-143 |
| 10.0-14.9 | 30 | 144-214 | 30 | 144-214 | 14 | 67-100 |

^1^ One sachet of RUTF (92 g) equals 500 kcal

The distributor will careful instruct caregivers on how to give the RUTF (in this case Plumpy Nut from Nutriset production). This information will follow the national guidelines (24), but underline how other foods should complement the treatment. Briefly it will contain the following messages

- RUTF is a medicinal food intended only for very thin patients, and it should not be shared with other children or family
- Give plenty of clean water or breast milk to the child. RUTF is a very dry food, and the child needs to drink more than usual
- The child may not want to eat when s/he is ill. Give small amounts of RUTF frequently and feed the child 5-8 times a day, but do not force feed
- If the child asks, other foods can be offered when the daily ration is consumed
- If the child is still breastfed, it is important to continue breastfeeding
- Wash the child's and your own hands with soap before feeding
- Keep RUTF in hygienic conditions and in covered containers.

## Rationale

- - 1. **Current challenges in the management of severe acute malnutrition**

Governments and (International) Non-Governmental Organizations have used RUTF since its development early 2000’s. As outlined before, the current RUTF ration has been taken directly from what was provided in inpatient settings and not adapted to the outpatient model where treatment compliance and observed rate of weight gain are different. Dietary treatment in supervised inpatient settings focused on maximizing weight gain to reduce duration of hospital stays.

Children with SAM have increased energy needs, both to maintain the basic metabolic activities (80-100 kcal/kg/day) and for catch-up growth (25–27). Energy needed for catch-up growth depends on the rate of weight gain and on the different types of tissue that need to be replaced in the body. The tissue deficit in uncomplicated SAM is about 50% fat and 50% lean tissue (28). Assuming the corresponding weight gain will consist of mixed tissue (50% lean and 50% fat), the energy required per gram weight gain is 4.9 kcal/g (29). However, allowing for 10% malabsorption in the malnourished child, the energy required to synthesize 1 g of mixed tissue is about 5.5 kcal/g (29). With the current dose of 175 kcal/kg /day, assuming 80-100 kcal/kg/day for maintenance, a weight gain between 14 and 17 g/kg/day is expected. Thus, using the same underlying assumptions, the energy intake behind an observed average rate of weight gain of for example 5 g/kg/day, would be 108 - 128 kcal/day. In this example, 25-40 % of the distributed energy was not converted to weight gain and as such regarded as waste.

There is some concern that rapid weight gain in early life may cause increased risk of chronic diseases in later life. Undernutrition in early life is associated with increased risk of adult diabetes (30), but it is not clear if this is mediated by rapid weight gain during treatment of acute malnutrition or by the undernutrition *per se*. Rapid weight gain in infancy, even followed by growth faltering, was associated with higher BMI and earlier menarche in a recent cohort study from South Africa (31). Thus, a slower rate of weight gain, even during recovery from SAM might be desirable in a life time perspective.

Initiatives investigating alternative dosages are rare and robust evidence to draw solid conclusions is lacking. As indicated in the latest Cochrane review on the use of RUTF, there is insufficient evidence to reach definite conclusions regarding differences in clinical outcomes in children with SAM who are treated with RUTF, standard diet or different quantities of RUTF (32). It raises the need for adequately powered randomized controlled trials to assess effectiveness of different treatment models.

**Main objective of the MANGO study:** Considering the lack of understanding of the different possibilities for the management of malnutrition, we propose to conduct a randomised trial among children treated for SAM in CMAM programs. We will assess the effect of a reduced ration of RUTF on rate of weight gain and recovery rate compared with the standard ration of RUTF. In addition, we will evaluate the cost-effectiveness of the two interventions.

In addition to the main objective, we shall investigate several other aspects that are key to better understand the physiology and the needs of acutely malnourished children. Indeed SAM children are often deficient in a range of nutrients. RUTF is designed to provide sufficient energy for normal energy requirements plus catch up growth assuming it is the only food consumed during recovery from SAM. However, the micronutrient content in RUTF is also higher than the recommended nutrient intakes (RNI) to allow for extra needs for tissue accretion and replacement of lost micronutrient reserves. Depending on the age group, the content of most micronutrients in RUTF are 2-3 times higher than RNI for healthy children (

Table 2) (29). Deficiencies of vitamin A and iron are among the most common micronutrient deficiencies related to childhood undernutrition and are both linked to compromised immune function (33). There is a need to address to which extent CMAM programs with their assumed lower than intended intakes of RUTF followed by lower rates of weight gain are effective in treating deficiencies of vitamin A and iron and to prevent deficiencies during catch up growth. Worth mentioning, replenishment for essential nutrients mainly happens in the first few weeks of the treatment, whereby a reduced dose can be provided in a second phase where household diet is given in addition to RUTF (19).

Table 2 Recommended nutrient intakes (RNI) for healthy children and RUTF content of vitamin A and iron. Values are expressed as nutrient densities (amount/1000 kcal).

|  | RNI^1^ | RUTF^2^ |
| --- | --- | --- |
| Vitamin A (µg/1000 kcal) | 743 | 1500 |
| Iron (mg/1000 kcal) | 17.8 | 24 |

^1^ (34)

^2^ (29)

Weight gain and its daily rate being the principal outcomes in the study, we should note that the quality of this weight gain is also of interest. Malnourished children present both a reduced lean as well as fat mass and the nutritional rehabilitation is supposed to re-establish both tissue deficits. However, a recent study has suggested that children discharged as recovered from malnutrition programs still present a lower lean mass compared to normal children from the same context (35). Indeed is seems that fat mass is more rapidly re-established than lean mass (36,37). Would children receiving different doses of RUTF gain weight differently in terms of tissue quality? And if the weight gain velocity is slower, would that imply a different tissue type being de deposited? While it will be interesting to characterize the different type of weight gained it should be noted that no reference exists to date to describe “normal” body composition among children. To enable a more clear interpretation of the results, a convenience sample of non-malnourished children will be recruited among the children present at the same health centers where the study takes place.

As the study hypothesis is that children in the 2 groups are going to have the same weight gain velocity, we envisage two possible ways for this to happen: either the consumption of RUTF is indeed identical between the groups, which would mean that the control group shares more of the product and the reduced dosage group optimizes the use of the RUTF better. Or, in the second case, the consumption of RUTF is different between the 2 groups but the children in the reduced dosage group compensate the “lack” of RUTF by consuming more family foods and thus have a similar energy intake to the control group. These 2 scenarios will be studied through 2 means: 1) a weekly questionnaire on the consumption of RUTF and other foods by the child being treated and 2) a more detailed dietary recall at the 4^th^ week to estimate the quantity and the quality of foods consumed by the child during 2 consecutive days when still on RUTF treatment.

Finally, to better understand the different values that users attach to the therapeutic product we propose to investigate the perceptions of communities related to RUTF. This socio-anthropological study is needed to gain a more global and comprehensive understanding of the uses of the product that very likely go beyond the medical ends it is meant for. This information is crucial to take into account not only to better adapt our communication strategy with the beneficiaries but also when considering the next steps after the project. The economic, social and cultural values attached to the product will help us to interpret our results in the light of the reality in the field and therefore better prepare our response to the question of optimizing the treatment of acute malnutrition.

**Secondary objectives of the MANGO study:** We propose to investigate the micronutrient status of malnourished children at the start and at the end of the treatment. In addition, we are going to measure the body composition of malnourished children as well as their dietary consumption in the course of the treatment. Finally we are going to evaluate the perception of communities around RUTFs in order to better understand the values attached to this therapeutic product.

- - 1. **Current challenges in the diagnostics of severe acute malnutrition**

The MANGO study will serve as a host for one component of a multi-centric observational study, the Optidiag study. This study will compare different types of anthropometric diagnoses of SAM accounting for a range of indicators of nutritional needs and mortality and response to treatment (see Annex 7 Optidiag study). The Optidiag study is mainly extracting existing data from the MANGO study with only one addition: a point-of-care test (leptin), which will be analysed using rapid field tests and can be accommodated within the 2*2.5 ml blood already collected at admission and discharge and hair samples at admission and discharge.

Recent work on metabolomic analysis and micro-assays used to characterize the metabolic status of malnourished Ugandan infants and young SAM children revealed that a major biochemical predictive factor for mortality is low-level leptin (38); leptin is a marker of adipose tissue reserve and a critical modulator of immune function. These findings suggest that fatty acid metabolism plays a central role in the adaptation to acute malnutrition and that a low level of adipose tissue hormone leptin is associated with, and may predict, mortality prior and during treatment (38).

Isotopic analysis of stable carbon and nitrogen in human hair can be investigated and measured throughout the course of nutritional deprivation to reconstruct the onset and duration of undernourishment (39) as well as tracing the temporal evolution of nutritional status (36,40–43). Variations in stable isotopic signals, specifically δ13C and δ15N are linked to the catabolism of body fat and protein deposits respectively; variations in these stable isotopic signals can be assessed to understand the influence of diet as well as other physiological factors on an individual (44). Since keratin remains unchanged after synthesis, and the speed of hair growth is constant (around 2.5 mm per week), weekly information on protein-energy metabolism can be traced back along the hair follicle, thereby indicating not only the severity of the episode of wasting but also the metabolic effects of the nutritional rehabilitation (on both lipid and protein anabolism). Isotopic evaluation of stable carbon and nitrogen in hair will therefore be used to create a retrospective timeframe of nutritional status and trace the physiological recovery of children during SAM management.

The presence of ketones in the urine, indicating lipid catabolism (fat tissue disintegration and rapid weight loss) was evidenced during fasting and SAM (41,45). Metabolic status for SAM children at the time of enrolment in CMAM has been characterized by ketonemia; yet, lipolysis decreases in response to nutritional rehabilitation suggested by total ketones (41). Moreover, biomarkers of urinary infections like urinary nitrites and urinary leucocyte esterase (LE) have also been shown to be associated with an increased mortality risk in SAM children (46). Positive dipstick urinalysis administered as a bedside screening test for either nitrates or LE is associated with a higher case fatality and was shown to be a strong predictor of mortality in children admitted with SAM (46).

Thus we propose to collect non-sterile urine samples when possible to investigate these biological parameters through the urinary multiple indicator strips (e.g. Roche laboratory, or Combi Screen of Analyticon).

## Outcome measures

The primary outcome will be rate of weight gain (in g/kg/d) until discharge or other endpoint. Rate of weight gain is not uniform across the entire recovery period: children who default early may have a high rate of weight gain (in g/kg/d) because weight gain is highest in the first few weeks when the undernutrition is more pronounced. Thereby, children who leave the program early, and before being defined as recovered, may have a high rate of weight gain solely due to their short stay. If defaulter rates differ between the two groups, the rate of weight gain expressed as g/kg/d will be affected. Therefore, in addition to the summary measure rate of weight gain (g/kg/day), weight (g) will also be analysed as a function of time using mixed linear models where the variation ascribed to individual can be adjusted for. Different confounders such as weight at admission, age and sex can be adjusted for and treatment phases and treatment outcome can be included in the model. In any case, rate of weight gain is directly influenced by amount of RUTF consumed, and will be sensitive to any negative effect of a lower ration of RUTF.

Other dimensions of anthropometric changes will be measured such as growth velocity for MUAC, height and body composition, and included as supporting outcomes.

Since the cost-effectiveness of a program depends on duration of treatment as it affects human resources as well as total product need per child treated, length of stay (days from admission to discharge from the program) will be used as secondary outcome. From a programmatic point of view, rate of weight gain and length of stay as well as recovery rate need to be considered together in order to evaluate the quality of the program.

# Objectives

## General objective

To assess the efficacy and cost-efficacy of a reduced RUTF dosage in children aged 6 to 59 months treated for uncomplicated severe acute malnutrition. The overall aim is to contribute to improving accessibility and coverage of SAM treatment in Low and Middle Income Countries (LMICs) through optimizing the amount of RUTF needed per treatment i.e. reducing it.

## Specific objectives


Primary objective

In the context of outpatient treatment of SAM in children 6-59 months of age, to assess the effect of a reduced dose of RUTF on:

- **Rate of weight gain** (g/kg/d) in children 6-59 months of age from admission to discharge

Secondary objectives

In the context of outpatient treatment of SAM in children 6-59 months of age, to assess the effect of a reduced dose of RUTF on

- **the duration of treatment** (days) until recovery
- **recovery rate, defaulter rate, and mortality**
- **cost-efficacy** (average and incremental costs per child recovered) of the CMAM program

**Auxiliary objectives**

In the context of outpatient treatment of SAM in children 6-59 months of age, to assess the effect of a reduced dose of RUTF on:

- Growth velocity of **anthropometric variables and indices**
- **Vitamin A and iron status**
- **Body composition** of children
- **Proportion of energy intake from family foods** 4 weeks after initiation of treatment
- **Relapse rate** until 3 months after discharge
- **Caregivers perceptions** around RUTF and sharing practices at household level (prior to the study) and to compare practices between the two treatment groups

# Methods

## Study design

The study is a randomized controlled, parallel non-inferiority trial. Children will be individually randomized to receive either normal-RUTF dosage (control) or reduced-RUTF dosage (intervention) (See **Figure 2**).

Randomization will be stratified by ten health centres which will be selected in collaboration with local authorities based on the following criteria:

- SAM caseload of the health centre (minimum 7 admissions per month)
- Accessibility of the health centre even in the rainy season to allow weekly treatment visits without interruption

In addition to the RUTF distributed to the caregivers according to the group allocation, all participants will be treated according to the standard of care outlined in the Burkinabe national protocol for management of SAM (24) . This includes medical check-up and standard treatment for infections as well as referral to hospital for any complications requiring medical attention.

Children and caregivers will be appointed weekly at the health centre for medical examinations and replenishment of RUTF for one week’s treatment according to the random sequence. After discharge (WHZ ≥ -2, or MUAC ≥ 125mm, or WHZ ≥ -2 and MUAC ≥ 125mm, depending on the admission criteria used), relapse rate will be assessed monthly for 3 months. Children will be appointed for fortnightly follow-up visits at the health centres.


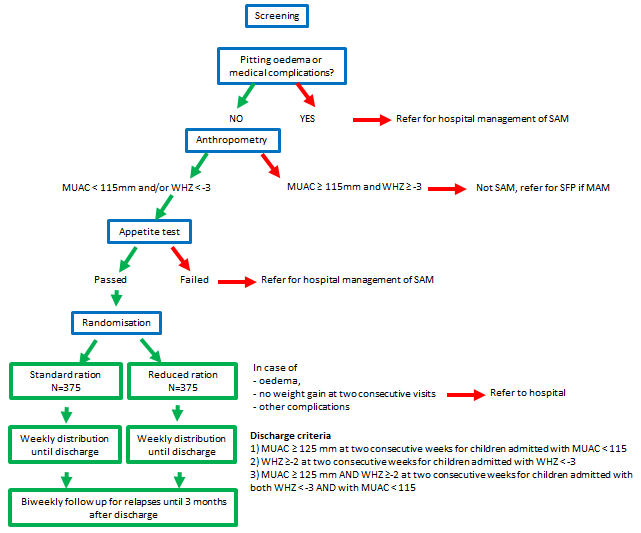


Figure 2 ADMISSION AND PATIENT FLOW OF PARTICIPANTS IN the mango study

## Blinding

This is an open trial since participants cannot be blinded with respect to the amount of RUTF. However, participants will not be directly informed, which group they are in, and dosing will be different according to the child’s size and treatment stage, thereby masking the intervention group. The research staff engaged in measurements and interviews will be kept unaware of the intervention group. Collection of empty sachets and distribution of RUTF at weekly visits will be conducted by the distributor, the only person in the field with access to the intervention codes.

Likewise, treatment allocation will be blinded to investigators until data cleaning and initial data analyses have been completed. The main data file will contain a unique study ID, but will not contain information on intervention group. The intervention code will be kept in a separate file, only available to a person responsible for the randomization lists, and not directly involved in data analyses. Once data collection and data cleaning is complete, a file with two unnamed groups will be released to enable assessment of confounding, and only after decision has been made about the final analysis and which variables to adjust for, the code will be broken into high/low dose of RUTF.

An independent data safety and monitoring board (DSMB) will get access to the code file for interim analyses during data collection.

## Randomization and blinding

Recruitment of subjects in the study is expected to take place from August 2016 to July 2017. Stratified, blocked randomization will be used to randomly allocate children to the two treatments. Stratification will be done by recruitment site, i.e. separate random sequences will be made, one to be used at each of the ten sites. This will ensure that all study sites, which may be strongly associated with the outcome and thus important potential confounders, will be equally distributed between the groups. Blocked randomization will be used, with varying block size, to ensure that for each site, children will be allocated evenly to the two groups at any given point of time. The random sequences will be generated by a person not directly involved in conducting the study using [www.randomization.com](http://www.randomization.com). The lists will be printed and stored in a sealed envelope at ACF headquarters and at field level. The Principal Investigator (PI) will extensively train 1 person (distributor) per mobile research team on how to read this list and assign the treatment to the study participant at each visit. Without compromising the concealment, the PI or the field supervisor will conduct regular quality control and supervision of randomisation procedures to preserve the integrity of the management of the randomization lists. The treatment allocation list will not be shared and the distributor will not be involved in assessment of any of the outcomes except adherence to and distribution of RUTF. All research staff, including the PI, will be blinded to the treatment code and the assigned treatment per study participant will only be disclosed after data collection has been finalized.

Before enrolment to the study, all participants will be made aware of the different treatments and it will be explained that the allocation of treatment will be done randomly and participants can hence not choose which treatment their child will receive (see information letter and consent form in annex 1 and 2).

## Study population

## Inclusion criteria

- Children aged 6-59 months
- Diagnosed with uncomplicated SAM and eligible for CMAM treatment, defined as
  - WHZ <-3 and/or MUAC <115mm
  - No pitting bilateral oedema
  - Passed a standardized appetite test
  - No medical complications requiring hospitalization as per Burkina protocol (see table 3)
- Resident in the catchment area at the time of inclusion
- Caregivers consent for the child to participate

## Exclusion criteria

- Severe anaemia defined as haemoglobin concentration < 4g/dl. Children with severe anaemia will be referred to the appropriate health facility for care
- Plans to leave the catchment area in the next 6 months
- Known peanut and/or milk allergy
- Treatment for SAM already received within the last 6 months, including re-admissions after defaulting, relapses, medical transfers
- Malformation or handicap which may affect food intake such as cleft palate, cerebral palsy, Down’s syndrome

## Consent, inclusion and follow up of participants

Caregivers who bring their children to the health centre where these are screened and diagnosed with SAM, are referred towards the research team and invited to participate to the study if eligible.

Once the children have been referred and screened eligible (with a positive appetite test and other criteria) to be included to the MANGO project, the caregivers are informed about the study in the local languages (mooré and gourmantchéma) and an information letter is distributed by the research team (Annex 1). Thereafter, they are invited to participate, and if they consent, asked to sign or thumb print the consent form (Annex 2).

Those who do consent will be given consecutive health centre specific study ID numbers which will determine which intervention group they belong to, according to the random sequences. The RUTF distribution takes place after all assessments and measurements. Finally, a socio-economic questionnaire is administered at the end of the admission visit. Each participant will receive an appointment card with study ID, name and age of child and name and contact phone number of caregiver. Caregivers will be informed to bring the appointment card at each visit. The unique study ID number will be added to all participant documents and entered in all databases. Furthermore, participation in the study will be registered in the child’s health card to minimize the risk of double registration or participation in other feeding programs. The participants will be followed and assessed according to the activity plan in **Table 3.**

Table 3. Overview of participant flow and study activities

|  | CMAM | | | FOLLOW UP |
| --- | --- | --- | --- | --- |
|  | Admission | Weekly visits | Discharge | Bi-weekly for 3 months |
| Screening for eligibility | x |  |  |  |
| Information, consent and randomisation | x |  |  |  |
| Socio-economic questionnaire ^1^ | x |  |  |  |
| Morbidity questionnaire | x | x | x | x |
| Clinical assessment | x | x | x | x |
| Anthropometry ^2^ | x | x | x | x |
| Blood sampling ^3^ | x |  | x |  |
| Distribution of RUTF | x | x |  |  |
| Supplement adherence and acceptability |  | x | x |  |
| Dietary intake |  | wk 4 |  |  |

## Recovery

A child will be considered **recovered** when he/she reaches one of the 3 following criteria (corresponding to the admission criterion):

- For those admitted based on WHZ < -3 (but MUAC>115mm): WHZ ≥ -2 at two consecutive visits and the absence of illness
- For those admitted based on MUAC < 115 mm (but WHZ ≥ -3): MUAC ≥ 125mm at two consecutive visits and the absence of illness
- For those admitted based on WHZ < -3 and MUAC < 115 mm: WHZ ≥ -2 and MUAC ≥ 125mm at two consecutive visits and the absence of illness

Discharge criteria should be met within 16 weeks after admission and child should have no oedema nor medical complications. The minimum duration is treatment is therefore two weeks for children who have reached discharge criteria already after the first week of treatment, and the maximum treatment is 16 weeks. If discharge criteria are not met within 16 weeks the child will be referred for further examination and/ or inpatient treatment.

## Defaulters and lost to follow-up

Subjects who spontaneously discontinue their participation in the research project are defined as drop-outs including withdrawal of consent, defaulting and lost to follow-up. Participants are free to withdraw from the study at any point of time, as explained more in depth under ethical considerations (see section 7). Whenever possible, the caregiver will be advised to continue treatment under the national nutrition program if the child has not yet successfully reached discharge criteria. Where relevant, data collected until the point of defaulting will be used in the data analysis, unless otherwise indicated by the caregiver. Where relevant, available data from confirmed defaulters and participants lost to follow-up will be used in the data analysis until the last contact. Definitions and actions related to dropouts are outlined in **Table 4**. Drop-outs will not be replaced as loss to follow up has already been taken into account in the sample size considerations.

Table 4. Definitions of and action related to withdrawals and defaulters

| Reason for withdrawal | Further follow up | Data analysis |
| --- | --- | --- |
| Withdrawals |  |  |
| Explicit withdrawal of consent | None | Use relevant data until withdrawal  Record final outcome as defaulted before discharge |
| Defaulter (missed three consecutive visits, confirmed alive at home visit, and not interested to come back) | Refer to standard treatment if child has not recovered | Use relevant data until withdrawal  Record final outcome as defaulted before discharge |
| Lost to follow up (missed three consecutive visits, with no contact after standardised tracing procedures) | None | Use relevant data until withdrawal  Record final outcome as unknown |

## Referrals

At any time during the treatment, from admission to the final follow up deterioration of the child’s condition will be closely monitored and appropriate action taken (Table 5). As part of the routine CMAM programme at the health centre, children will undergo a medical examination at each weekly visit. While this is the responsibility of the regular staff at the health centres, the project staff will be co-responsible for detecting medical condition requiring hospital treatment and will assist in facilitating referral to inpatient treatment.

Table 5. conditions requiring referral for inpatient treatment

| Referrals |  |
| --- | --- |
| Condition | **Action** |
| Child develops oedema or other condition requiring hospitalisation | Refer for inpatient treatment |
| No weight gain for 4 consecutive visits and/or weight loss for 3 consecutive visits | Check situation, refer for inpatient treatment |
| Any other danger sign such as  - Edema,  - Fever >39°C or hypothermia <35°C  - severe dehydration  - repeated vomiting  - severe respiratory difficulty  - Haemoglobin < 4 g/dl  - Severe malaria  - Abscess or large skin lesions requiring IM or IV fluids  - Very weak, apathetic, unconscious  - Convulsions or faintness | Check situation, refer for inpatient treatment |

## Sample size

As a basis for the sample size considerations we have used data from 4605 children participating in ACF administered CMAM programs in Burkina Faso (unpublished data). The mean (SD) rate of weight gain among all participants was 3.5 (2.6) g/kg/day. We assumed an expected difference between the two intervention groups of 0 g/kg/day. We applied a non-inferiority margin of 0.5 g/kg/day (difference between two groups of up to 0.5 g/kg/day not considered different) and a minimum acceptable mean rate of weight gain in any group of 3 g/kg/day. Applying these considerations to a standard sample size calculation for non-inferiority trials (47) and assuming a power of 80% and a 5% significance level, 335 children are needed in each group to verify that the maximum true difference between intervention groups is not larger than 0.5 g/kg/day. Allowing for a drop-out of 20% the total target sample size should be 800 children.

## Pilot study and training

Before launching the main trial, a feasibility phase will be conducted to experiment the methodologies, the research instruments and the procedures to be used in the main study.

The research staff will be extensively trained in all aspects of the study procedures from recruitment of participants, through administration of questionnaires and other data collection tools to measurements and blood sampling. All study procedures will be documented in the study manual consisting of a set of standard operating procedures (SOPS).

The following methodologies will be tested in the pilot study:

- Eligibility criteria, including appetite test and assessment of oedema
- Anthropometry (weight, height/length, MUAC)
- Explanation of the study to caregivers including the consent procedure
- Randomization procedures
- Acceptability of the new dosage by families
- Blood collection procedures
- Cold chain procedures
- Data collection tools such as questionnaires and methods for data entry using tablets
- Tracing, referral and follow up procedures
- Key messages for targeted population based on socio-anthropological study
- Visual analogue scale adaptation to local context
- Quality control procedures.

During the feasibility phase volunteer participants will be recruited from the MANGO health centres and include both healthy and malnourished children and their caregivers. Clinical procedures will be tested on the children and questionnaires administered to the mothers. A small compensation has been reserved for the participants and the content of this will be defined together with local health professionals.

The procedures mentioned in the following sections will be adapted to study context after completion of the pilot study.

## Questionnaires

All data will be collected using tablets and thus will be available immediately in electronic form at the office. Every individual questionnaire will be linked to the rest of the data by the same individual with the help of the unique ID number each patient has. To ensure the availability of key information at the field level, registries will be developed to help and monitor each patient and enable the field teams to appreciate the advancement of the project.

A baseline socio-economic questionnaire will be administered upon admission. This information will be collected through individual interview with the caregiver. The questions include socio-economic indicators, family size, income and expenditure and medical history of the child. Additional humanitarian assistance received by the household will be recorded to control for other sources of nutritional inputs. An initial mapping of NGO/UN/Governmental community support to villages will allow the enumerator to probe, if necessary.

In addition, the caregiver will be asked weekly to score the child’s health on a visual analogue scale. This data will be compared to the child’s nutritional progress to assess the relationship between maternal perception and quantitative data.

Since there is no objective way of quantifying the accumulated consumption of RUTF, the adherence to the treatment (RUTF quantity consumed) will be assessed weekly by maternal recall.

## Dietary intake

The semi-quantitative dietary intake information aims at estimating the contribution of household foods to the total energy intake. The main caregiver will be interviewed to obtain dietary intake information of the child. Within the context of Burkina Faso, estimating dietary intake is challenging since family members eat from a common plate. To obtain detailed food intake and nutrient intakes of a child is therefore difficult. The aim is to estimate energy intake and distribution and dietary diversity.

**A 24h dietary recall** focusing on energy intake will be conducted at week 4 of the treatment of SAM using a pre-coded data collection form with standard portion sizes. The caregiver will be interviewed regarding the dietary intake of the child the day before the interview. In case the child was fed by another person it will be recorded, and it will also be recorded if the day before the interview was representative of the child’s normal meal pattern at this point of time. The 24h recall will be built chronologically and start from the time the child woke up the day before until the child woke up on the day of the interview. The caregiver will be asked to recall all the foods the child had to eat yesterday: both the quantity of the meal components and the ingredients used to prepare them will be asked. The caregiver will also be asked to describe (qualitatively) the meals ingested by the child in the 2 days preceding the 24h recall day. The interviewer will be allowed to probe for snacks between meals as they are often forgotten. RUTF consumption will be specifically addressed by the interviewer. As part of the pilot study, a list of commonly eaten foods and dishes will be established and a data collection form constructed using household measures and standard portion sizes (small, medium and large) to aid the estimation of portion size (48). Standard recipes of dishes will be constructed to convert amount of food to energy intake.

**Dietary diversity score** (DDS) expressing the number of food groups consumed the previous day will be calculated according to the food groups recommended by FAO (49) and used to describe the quality of the family food consumed and to complement the interpretation of vitamin A and iron status outcomes (biological indicators).

## Anthropometry

Weight, height, MUAC and oedema will be measured in all children. All anthropometry will be measured weekly.

Weight will be measured to the nearest 0.1 kg with an electronic scale (SECA scale) and will allow for double weighing of caregiver and child, i.e. weighing the caregiver first, zeroing the scale and allowing the caregiver to stay on the scale and hold the child while its body weight is measured. A standard weight of 5 kg will be used for daily calibration of the scale and a wooden plank will be used to ensure the scale is in a horizontal and stable position.

Length and height will be measured to the nearest 0.1 cm with a wooden height board with graduated index strips in millimetres on each side (ACF model). A standardized length stick will be used to check the accuracy of the equipment. Children below 2 years of age will be measured lying down and older children will be measured standing. To facilitate estimation of age for children who do not bring birth or vaccination certificate or other documented age, a local event calendar (including major festivals, constructions, religious events during the last five years, and seasons before and after events) will be used to estimate age (50). In case the age cannot be verified or estimated, children less than 87cm will be measured lying down. Children 2 years or older who are not able to stand, will be measured lying, and 0.7 cm will be subtracted the recumbent length during data analysis and for the purpose of W/H Z-score interpretation (51).

MUAC will be measured with a non-stretchable MUAC tape on the left arm to the nearest mm.

Anthropometry will be measured and recorded twice. Two people are necessary for taking the measurements alternating between the role of the measurer and assistant. The measurer will take the first measurement and note it down and then the roles are switched and the assistant takes the second measurement. In case of large difference between the measurements, the procedure will be repeated but this time with the team supervisor. All measuring tools will be calibrated and checked daily for accuracy and replaced if needed. To ensure good quality measurements from start to finish, calibration sessions will be held regularly.

## Body composition

The body composition of children will be measured with the help of a bio-electrical impedance measurer. This will be done at admission and at discharge to look into potential differences in the quality of weight gained between the 2 study groups: if a group gains more fat mass or more lean mass than the other group. In addition, we shall evaluate which group comes closer to the reference group of normal children. In order to do this, we plan to measure the body composition of approximately 50 children without acute malnutrition who will serve as a reference group.

The body composition parameters will be measured using NutriGuard device (from Data Input, Germany) and following the protocol described elsewhere (52). Self-adhesive disposable electrodes will be attached to the right hand and foot of the patient. Measurements will be taken in duplicate, each spaced 5 minutes apart, while children are supine with limbs abducted from the body. Bio-electrical impedance vector analysis (BIVA) will be used to analyse the data.

## Clinical assessment and search for oedema

Oedema will be measured by applying thumb pressure on top of both feet for three seconds. In case pits are observed at the feet, the same procedure will be repeated at the lower legs to check for the degree of oedema.

The nurse will conduct a standard weekly clinical assessment of the child (i.e. temperature, respiratory rate, pulse rate, cough, ear and nasal discharges) according to the national protocol for management of SAM (24). Symptoms, diagnosis and treatments prescribed will be recorded. The clinical assessment will also serve to monitor development of complications which need inpatient care. All serious adverse events or development of complications will be immediately reported to the study supervisor and the child referred to inpatient treatment if needed (see 3.8).

## Blood samples and analyses

2.5 ml of venous blood sample will be collected by standard phlebotomy in serum tubes using closed vacutainer system.

Before coagulation, one drop of fresh whole blood will be used to estimate haemoglobin concentration using HemoCue (HemoCue®301), one drop will be used for malaria rapid-test (SD Bioline Malaria).

Once the sample has been obtained it will be stored in an ice box until transport to a central lab at the end of the day for centrifugation and separation of serum. Serum will be transferred to microvials in aliquots of 0.2 ml, labelled with unique sample ID numbers containing study ID and visit number and stored at minimum -20⁰C until shipped for external laboratories for analysis in batches. The maximum duration of storage will be 20 months.

Serum retinol binding protein (RBP) will be used to assess vitamin A status. Serum ferritin and serum soluble transferrin receptor (sTfR) will be used to assess iron status. In addition, the two acute phase reactants C-reactive protein (CRP) and α_1_-acid glycoprotein (AGP) will be measured and used to adjust for the effect of inflammation on the micronutrient status indicators. Inflammation is known to elevate serum ferritin and depress retinol binding protein as part of the biological acute phase response to inflammation (53–55). These five proteins will be measured in Germany using a special ELISA kit which analyses all five components (56).

Since no laboratory in Burkina Faso performs all the desired analyses for research purposes, the samples will be exported for analysis.

- 0.2 mL of serum from baseline and discharge visits will be sent to Germany (VitMin lab, Dr Jürgen Erhardt) for analysis of C-reactive protein (CRP), α_1_-acid glycoprotein (AGP), ferritin, Retinol Binding Protein (RBP), and soluble Transferrin Receptor (sTfR) (57).

## Qualitative study on RUTF sharing practices

Prior to the launch of MANGO trial, during feasibility test, a socio-anthropologist will be assessing perceptions among families and opinion leaders in terms of health seeking behaviour for children, child food culture and attitudes, food taboos and favoured foods, attitudes to food for malnourished children etc. Health personnel in health centres as well as in the community will be also assessed on the same topics and ACF staff as well.

*Qualitative data* collection methods will be used to obtain information regarding sharing practices of RUTF, perceptions of the treatment and food intake through the use of individual and focus group discussions. These interview sessions will be organised by teams of two; one person will lead the interview and the other will facilitate the recording. Interviews will be recorded and audio files will be transcribed by trained transcribers and translated to French.

The socio-anthropology assessment will be conducted prior to start MANGO inclusions.

Findings will be used to tailor communication to beneficiaries of SAM treatment on MANGO research, to estimate sharing practices of RUTF, and improve adherence to treatment and minimize relapse.

# Data and Safety Monitoring Board (DSMB)

The Data and Safety Monitoring Board (DSMB) is a group of independent experts that will advise ACF and the study investigators. The primary responsibilities of the DSMB are to 1) periodically review and evaluate the accumulated study data for participant safety, study conduct and progress, and, when appropriate, efficacy, and 2) make recommendations to ACF concerning the continuation or termination of the study. Stopping rules will be defined in agreement with the DSMB, once it has been established. It may be based on unequal defaulter rate, referral rate or mortality rate between the 2 groups.

Ideally, the DSMB will consist of a chairman, an external & independent statistician and a national independent researcher, all not involved at any level of the present research project, being independent from this initiative. A detailed overview of the roles and responsibilities and defined time intervals for review will be established before study start.

# Data management and analysis

## Data management

All questionnaire data and measurements will be recorded through digital data collection using tablets and the software Open Data Kit (ODK, www.opendatakit.org). The data manager will upload the data to a backup server every evening. The data will then be treated with Excel and STATA software to check the coherence, consistency and completeness. For anthropometric measurements we will apply the standardization tools available in ENA for SMART software (<http://www.nutrisurvey.de/ena/ena.html>). Regular supervision and refresher training workshops will be organised to maintain a high data quality.

In addition to the digital database, an OTP card with name, study id, date, age, length and weight at admission will be produced at enrolment. This will be kept at each health centre and new values of anthropometry entered at each visit to be able to track the child’s progress on the spot and to take action if the child’s situation is deteriorating. Field tables for conversion of anthropometry to z-scores will be available in the health centres.

Caregivers will receive an appointment card on which the date for new appointment will be written after each visit. A weight gain chart will be printed on the card, in which the child’s weight will be plotted at each visit enabling the caregiver to follow the progress of the child.

Laboratory data analysed in external laboratories will be merged according to study ID into the main dataset.

All participants will be registered in an individual registry where each visit will be registered with date of attendance. Participants who do not show up at the health centre will be contacted by telephone, and if not successful, a team of community health workers will try to trace the family at the home address and encourage them to go to the health centre for continuation of the treatment.

## Data analysis

All analyses will be done on an *intention to treat* basis, i.e. all study participants will be included in the analysis, irrespective of degree of adherence to their treatment group.

The **primary outcome** (rate of weight gain) will be standardized according to body weight and duration of treatment and calculated as

$Average rate of weight gain (g {kg}^{-1}{day}^{-1})=\frac{(discharge weight-minimum weight)}{minimum weight* length of stay}$

Weight is measured weekly during the study, and in case a child leaves the program before discharge criteria are met, the last measured weight and date will be used to calculate the total weight gain.

The **secondary outcome** (length of stay) will be calculated as the number of days between discharge and admission, the latter included. Length of stay is only relevant for children who are discharged as recovered.

Outcomes will be compared between the two treatment groups using T-tests for variables that are normally distributed. If necessary, variables will be appropriately transformed to conform to a normal distribution.

In randomised controlled studies there is generally no need to adjust for confounders. However, equal distribution of potential confounders between groups will be assessed at baseline and if intervention groups differ by chance, the identified confounders will be adjusted for in linear regression analyses using dummy variables for categorical explanatory variables. Potential confounders include age, sex, and degree of undernutrition (MUAC, WHZ and HAZ category) at admission.

Since defaulter rate might be influenced by the amount of RUTF given and thereby indirectly affecting the weight gain, the analyses will be conducted for all participants, as well as only for children who are discharged as recovered. Additional analyses will also be conducted using mixed linear models, where random effects of individual will be accounted for and confounders such as age and sex can be adjusted for and the effect in different phases of treatment can be estimated. As the potential confounders might also act as effect modifiers, two-way interactions between intervention group and age group, sex, and degree of undernutrition will be assessed.

There are no concrete plans to apply any concrete adjustment for multiplicity in the secondary analyses. Rather, the results will be considered with appropriate humility. All statistical analysis will be done using STATA® software or similar.

# Risks and assumptions

## Funding and resources

It is assumed that ACF technical support to Ministry of Health led nutritional programme in Fada district will continue to be funded by humanitarian donors in 2016 and 2017 (on a yearly basis). The presence of ACF in the district facilitates the work of the MANGO team who will be able to benefit from the ACF teams working with the routine staff to improve the treatment of acute malnutrition in the health centres.

Funding for direct research project costs has been secured through research grants from CIFF (Children’s Investment Fund Foundation) and ECHO (**European Commission**'s **Humanitarian Aid and Civil Protection Department**).

A constant supply and sufficient safety stock of RUTF is essential to the success of the project. Project staff will monitor the RUTF demand and supply weekly at each health centre. An action plan will be developed in case of eventual shortage of RUTF at the MANGO health centres to avoid and minimise all negative effects this could have on the project and to prevent a total stock out of RUTF.

## Community awareness and local cooperation

Study participants will be enrolled at health centre level and a smooth collaboration between ACF research project and the Ministry of Health and health centre staff is required for the study’s success. ACF has been collaborating closely with Ministry of Health for several years and is continuously improving this working collaboration.

A qualitative assessment will be conducted prior to the study to retrieve information on sharing and selling practices at community level, perception of malnutrition and perception of treatment. This information will be used to tailor the communication messages to the context. A dedicated communication officer will be recruited to ensure all community leaders and stakeholders are aware of the project.

## Recruitment rate and study population

The recruitment rate could be lower than anticipated either due to low case load, or due to eligible individuals refusing to participate. When selecting the 10 health centres where the study should be conducted, additional second choice health centres will be listed for easy expansion of the catchment area. However, if the number of health centres needs to be increased, it will result in the need for more study staff.

The generalizability of the results is limited to contexts similar to the study population in terms of age group, underlying conditions such as disease pattern, food insecurity etc. Most children are enrolled in a supplementary feeding program (SFP) after discharge from CMAM in the study area. Results on relapse after discharge have to be seen in this context and cannot immediately be translated to contexts without SFP.

Although HIV status might be a potential confounder or effect modifier, HIV status will not be assessed. The prevalence of HIV in Burkina Faso is very low (estimated prevalence among adults 15-49 years of age was 0.9% in 2014) and although an unknown HIV status may create noise, it is expected to be equally distributed between intervention groups, and should not affect the main conclusions in a randomized study design (58).

# Ethical considerations

This research protocol will be submitted for ethical clearance at the national ethical committee of Burkina Faso (Comité national d'éthique pour la recherche en santé (CNERS)) and the national clinical trials committee of Burkina Faso (Direction General de la Pharmacie, du medicament et des Laboratoires (DGPML)).

## Autonomy (Informed consent)

As this study is conducted in children less than 5 years of age, caregivers will consent on behalf of their child. Before enrolment in the study, study personnel will explain the study to the caregiver in local language. The same information will also be distributed in a written document that includes the aim of the research, the principle of random allocation of treatment, the expected duration of their participation, the measurements that will be done, and the potential health risks and benefits (see Annex 1). The caregivers will have the opportunity to ask questions before they decide whether to participate or not. It will be explained that the care of their child will not be affected if they decide to decline. In addition, it will be explained that they are free to withdraw from the study at any time, without any impact on their right to treatment. A proper feedback mechanism will be put in place as described in the Information Letter (Annexe 1). Participants who agree to take part in the research will then be asked to sign the consent form (see Annex 2). Illiterate caregivers will sign with thumb print, and an independent witness will sign to validate the fingerprint as signature.

Whereas the mother might be the primary caregiver and bring the children for health care, the father is often the decision maker in the study area. Therefore, it is essential that caregiver will get opportunity to contact her spouse before giving informed consent. For this purpose, a free phone call is proposed for the participants in need to consult their spouse. Also, it will be emphasised that the spouse can at any time contact the research personnel either by telephone or by coming to the health center in order to get more information on the study procedures.

## Treatment of complications and of patients not enrolled

Children with medical complications such as any grade of bilateral pitting oedema, evidence of systemic infection, or anorexia will be treated according to standard of care as defined in the national protocol for management of malnutrition (24) and referred to inpatient care. Children who present with moderate acute malnutrition during screening will be referred to the national SFP for treatment and will be advised to return to the health centre if the child’s status deteriorates or if the child develops illness. Children who are eligible but who decline the invitation to take part in the study will be enrolled in the standard CMAM program at the health centre and treated according to standard of care (24).

## Confidentiality

Confidentiality and anonymity will be applied at all stages of the research (data collection, data analysis, participant’s files, etc.). Names are kept in data files as long as the study is running, to be able to verify participant identity. A unique study ID number will be used for recording purposes and different data files merged by study ID. However, after data cleaning and verification, names and contact information will be removed from the data files. Photos and videos of participants will not be taken and or used without caregiver’s specific consent (Annex 2).

## Risks (do not harm)

The suggested amount of RUTF is lower than the standard care, and a close follow up of all children under treatment is necessary to monitor if children are deteriorating due to insufficient RUTF during the treatment. Children will return weekly to the health centre for check-up and a close follow up system will be applied. Reminder calls will be done prior to appointment and community health workers will be involved to visit households from which children were absent. Caregivers will be asked to return to the health centre as soon as their child falls ill or loses weight.

Key treatment messages will be given at admission (see section 1.3), including advice on how and when to give the RUTF, how to complement it with household food and feed according to the child’s appetite. Knowing that questionnaires and quantitative methods are not suitable for assessment of practices which deviate from recommendations, sharing practices of RUTF will be studied through qualitative methods, such as focus group discussions.

Children included in the study will be referred to inpatient treatment if medical complications develop. Data on these children will be used until referral and their final status will be classified as “referred for medical treatment”. Children who are discharged as recovered are enrolled in a SFP (1 sachet of RUSF per day). As part of the study, they will be followed up monthly during an additional 3 months to timely identify relapse and refer accordingly.

Blood sampling will be performed at admission and at discharge. Adequate communication and a gentle contact with the child will be sought to avoid traumatising experience. Veins may be difficult to find in children with SAM, and the nurse who is going to do the phlebotomy can try in 3 places: on the upper hand, mid arm or at the femoral site. In total, two attempts will be allowed per child after which the sampling will be considered failed. The total amount of 2x2.5 mL blood taken over a minimum of four weeks is well below the safety limits recommended for paediatric research, typically 3-5 ml/kg over 1-3 months (59).

Allergic reactions to ingredients in the RUTF (peanuts) are potential risks and the first dose of RUTF will be consumed under supervision by health staff as part of the routine appetite test. In case of known allergic reactions, the child will not be included and referred to inpatient treatment of SAM.

The study participants will benefit from a closer monitoring of their treatment progress compared to the routine CMAM program. There will be more focus on defaulters and intensive measures to encourage caregivers to bring their child for follow up. Reminder calls and home visits will be implemented as part of the study. Thereby, children are less likely to deteriorate at home.

Any unexpected problems that can be ascribed to the study will be covered through a patient insurance which ACF will sign up for.

## Benefits to participants and community

Each patient will have their appointment card that will be used to note down the scheduled follow up visits for the child. At the back side of the appointment card, a weight gain chart will be printed, and the child’s initial weight and target weight values noted and the evolution of the weight gain plotted on each visit so that the caregivers will be able to follow the treatment progress of their child.

In recognition of the efforts our beneficiaries have made when participating to the study, the households will receive 2 compensations: at the last nutrition program visit (that is at discharge visit) we have planned to take an instant photo of the child that will be immediately given to the caregiver. And then at the end of the follow up period after discharge a small compensation kit has been planned to be given to the beneficiaries including a bucket and soap.

A bracelet will be available for the children to choose from when they have completed blood samples.

The results will be published in international journals and presented at international and national conferences and meetings and made available to national and international policy makers. If the study concludes that the use of a lower dosage of RUTF is as effective as the standard regimen, ACF commits itself to advocate for its implementation in the national guidelines. With a lower dosage, the CMAM programs can work with higher degree of national autonomy, due to reduced RUTF consumption. It is always of high priority to apply cost effective health interventions, and particularly so in financially challenged health systems. In addition, the risk of excess RUTF being available at local markets, and used inappropriately for healthy individuals will be lower. This will also benefit the community, particularly in the context of nutrition transition and double burden of malnutrition.

# Dissemination of results

Results of the project will be translated into scientific articles submitted for peer review in internationally acknowledged scientific journals. An advocacy strategy will be developed with ACF advocacy department for the diffusion and dissemination of the results. After publication of the results, ACF will disseminate the findings through available networks such as CMAM forum, EN-Net and Field Exchange. The results and lessons learnt will be shared at national and international level with stakeholders through conferences and presentations. In order to reach a wide public, easy to read flyers and one-pagers displaying the main results will be developed and disseminated through ACF international network.

A local dissemination workshop will be organised where local health authorities are invited. The results will be presented and other lessons learnt will be discussed. However, even if the MANGO results are in favour of a lower dosage, it will be emphasized that national treatment guidelines from Ministry of health should always prevail the time necessary to update and upgrade treatment based on recent findings.

# Collaborating partners and conflicts of interest

The project ownership is shared between ACF-France and ACF-Burkina Faso and a number of external partners are involved to support the scientific quality. A steering committee and a working group have been established.

Multi-stakeholder Steering Committee comprising external experts to be responsible to make decisions on key steps of the project cycle during the inception and the implementation phase. Members:

- André Briend - University of Copenhagen / ACF International Scientific Council
- Henrik Friis - Professor of International Nutrition and Health - University of Copenhagen
- Anne Dominique Israel - Senior Nutrition & Health Advisor – ACF-France
- Myriam Aït-Aissa – Senior Research Coordinator – ACF-France
- Alexandre le Cuziat - Regional Operations Director – ACF-France
- Elise Rodriguez - Head of Advocacy & Analyses – ACF-France
- Thomas Loreaux - Country Director – ACF-Burkina Faso
- Cécile Salpéteur, Nutrition Research Project Coordinator, ACF-France
- Carlos Navarro Colorado, epidemiologist – CDC Atlanta
- Jonathan Wells, professor of International Nutrition – UCL, UK

Technical Working Group responsible for the design and preparation of the project: they are accountable to make different suggestions and provide argumentation to the steering committee who will take decisions based on that.

Members:

- Suvi Kangas- Head of Research Project MANGO – ACF France mission in Burkina Faso
- Cécile Salpeteur - Nutrition Research Project Coordinator – ACF-France
- Pernille Kæstel – consultant
- Abdul Aziz Biga Hassoumi - Head of Nutrition Department– ACF-Burkina Faso
- Nathalie Benarrosh - Coordinator of Research – ACF – Burkina Faso
- Olivia Freire - Nutrition Advisor– ACF-France

None of the project affiliates have declared any conflicts of interest.

# Management and financing

The management of the operational aspects of the projects will be divided between ACF headquarter and ACF mission in Burkina Faso. At headquarter level, the Nutrition Research Project Coordinator will lead the research team and the head of research project (HoRP) will follow up the research process during the preparation phase from HQ. During the field implementation phase, the HoRP will be based in the field to monitor the research activities and follow up at field level. The Technical Advisor in Paris, and the Head of Nutrition Department and Field Coordinator in Burkina Faso will follow up the implementation of the operational activities with the research project.

The Head of Research Project will be the focal person regarding coordination and communication on the project. She will be responsible to drive the working group and to involve scientific partners in the project. The HoRP will be assisted by a deputy for the management of the research team and as a back-up during her absence. Both will be based in Fada N’Gourma with frequent field visits to the involved health centres sites and with a few coordination meetings at Ouagadougou level. Two research teams will be recruited (see Annex 4 for team composition details).

The study is funded by research grants from various donors as detailed in **Table 6**.

Table 6. Funding sources for the MANGO project

| Donor | Duration | Amount | Phase |
| --- | --- | --- | --- |
| ACF internal funds | 2014 | 100,000 € | Phase 1 |
| HIF small grant | 2014-2015 | 20,000 £ | Phase 1 |
| ECHO Burkina | 2015- 2016 | 66,000 € | Phase 2 |
| CIFF | 2015-2019 | 1 028 000 USD | Phase 2 |
| ECHO ERC | 2016-2017 | 383 445 € | Phase 2 |

# References

1. Black RE, Victora CG, Walker SP, Bhutta Z a., Christian P, De Onis M, et al. Maternal and child undernutrition and overweight in low-income and middle-income countries. The Lancet. 2013;382(9890):427–51.

2. Collins S. Treating severe acute malnutrition seriously. Arch Dis Child. 2007;92(5):453–61.

3. Tomkins A and FW. Malnutrition and Infection − A review − Nutrition policy discussion paper No. 5. Nutrition. 1989;(5).

4. Black RE, Morris SS, Bryce J. Where and why are 10 million children dying every year? The Lancet. 2003;361(9376):2226–34.

5. Black RE, Allen LH, Bhutta Z a., Caulfield LE, de Onis M, Ezzati M, et al. Maternal and child undernutrition: global and regional exposures and health consequences. Vol. 371, The Lancet. 2008. p. 243–60.

6. Alderman H, Hoddinott J, Kinsey B. Long term consequences of early childhood malnutrition. Oxf Econ Pap. 2006;58(3):450–74.

7. Martorell R. The nature of child malnutrition and its long-term implications. Vol. 20, Food and Nutrition Bulletin. 1999. p. 288–92.

8. WHO. Guideline: Updates on the management of severe acute malnutrition in infants and children. Geneva; 2013.

9. WHO, WFP, UNSCN, Unicef. Community-based management of severe acute malnutrition. World Health Organization; 2007.

10. WHO. Management of severe malnutrition: a manual for physicians and other senior health workers. Geneva; 1999.

11. Diop EHI, Dossou NI, Ndour MM, Briend A, Wade S. Comparison of the efficacy of a solid ready-to-use food and a liquid, milk-based diet for the rehabilitation of severely malnourished children: A randomized trial. Am J Clin Nutr. 2003;78(2):302–7.

12. Ashworth A. Efficacy and effectiveness of community-based treatment of severe malnutrition. Food Nutr Bull. 2006;27(SUPPL.3):S24–48.

13. Ciliberto MA, Sandige H, Ndekha MJ, Ashorn P, Briend A, Ciliberto HM, et al. Comparison of home-based therapy with ready-to-use therapeutic food with standard therapy in the treatment of malnourished Malawian children: A controlled, clinical effectiveness trial. Am J Clin Nutr. 2005;81(4):864–70.

14. Guerrero S, Rogers E. Access for all, Volume 1: Is community-based treatment of severe acute malnutrition (SAM) at scale capable of meeting global needs? Vol. 1. London; 2013.

15. The Sphere project. The Sphere Handbook: The humanitarian charter and minimum standards in disaster response. 3rd ed. Greaney P, Pfiffner S, Wilson D, editors. The Sphere project. Rugby: Practical Action Publishing; 2011.

16. Puett C, Swan SH, Guerrero S. Access for all, Volume 2: What factors influence access to community-based treatment of severe acute malnutrition? Vol. 2. London; 2013.

17. Emergency Nutrition Network. Government experiences of scale-up of Community-based Management of Acute Malnutrition ( CMAM ). A synthesis of lessons. 2012.

18. Gaboulaud V. Stratégies pour la réhabilitation nutritionnelle des enfants de 6 mois à 5 ans malnutris sévères département de Maradi, Niger. Vol. 33, Report, Epicentre MSF. 2004.

19. James PT, Van den Briel N, Rozet A, Israël A-D, Fenn B, Navarro-Colorado C. Low-dose RUTF protocol and improved service delivery lead to good programme outcomes in the treatment of uncomplicated SAM: a programme report from Myanmar. Matern Child Nutr. 2015;n/a-n/a.

20. Ministère de la Santé Burkina Faso, Secretariat Général, Direction Génerale de l’information et des statistiques sanitaires, Direction des statistiques génerales de santé. Projections demographiques de 2011 à 2020 des régions et districts sanitaires du Burkina Faso [Internet]. Available from: http://www.sante.gov.bf/index.php?option=com_edocman&view=document&id=118&catid=20&Itemid=1133

21. Ministère de la santé du Burkina Faso, Direction Régionale de la santé de l’Est, District Sanitaire de Fada N’Gourma. Plan d’action 2015 District sanitaire de Fada N’Gourma. 2015.

22. Ministère de la Santé Burkina Faso. SMART 2013. Enquête nutritionnelle nationale Burkina Faso. 2013.

23. WHO. Global Database on Child Growth and Malnutrition. Cut-off points and summary statistics [Internet]. 2015 [cited 2015 May 21]. Available from: http://www.who.int/nutgrowthdb/about/introduction/en/index5.html

24. Ministere de la Sante. Protocole national de prise en charge de la malnutrition au Burkina Faso. Ouagadougou, Burkina faso; 2014.

25. Ashworth A, Millward DJ. Catch-up growth in children. Nutr Rev. 1986;44(5):157–63.

26. Fjeld CR, Schoeller DA, Brown KH. A new model for predicting energy requirements of children during catch-up growth developed using doubly labeled water. Pediatr Res. 1989;25(5):503–8.

27. Spady DW, Payne PR, Picou D, Waterlow JC. Energy balance during recovery from malnutrition. Am J Clin Nutr. 1976;29(10):1073–88.

28. Golden MH. Protein-energy interactions in the management of severe malnutrition. Clin Nutr Edinb Scotl. 1997;16 Suppl 1:19–23.

29. Golden MH. Proposed Recommended Nutrient densities for moderately malnourished children. Food Nutr Bull. 2009;30(3 SUPPL. 1).

30. Jiang X, Ma H, Wang Y, Liu Y. Early life factors and type 2 diabetes mellitus. J Diabetes Res. 2013;2013(Figure 1).

31. Salgin B, Norris SA, Prentice P, Pettifor JM, Richter LM, Ong KK, et al. Even transient rapid infancy weight gain is associated with higher BMI in young adults and earlier menarche. Int J Obes 2005. 2015;epub ahead of print.

32. Schoonees A, Lombard M, Musekiwa A, Nel E, Volmink J. Ready-to-use therapeutic food for home-based treatment of severe acute malnutrition in children from six months to five years of age. Cochrane Database Syst Rev. 2013;6(6):CD009000.

33. Müller O, Krawinkel M. Malnutrition and health in developing countries. CMAJ Can Med Assoc J J Assoc Medicale Can. 2005;173(3):279–86.

34. Institute of Medicine. Dietary reference intakes. Food and nutrition board, editor. Washington, D.C.: Natrional l Academy of Sciences; 2001.

35. Bahwere P, Balaluka B, Wells JCK, Mbiribindi CN, Sadler K, Akomo P, et al. Cereals and pulse-based ready-to-use therapeutic food as an alternative to the standard milk- and peanut paste-based formulation for treating severe acute malnutrition: a noninferiority, individually randomized controlled efficacy clinical trial. Am J Clin Nutr. 2016 Apr;103(4):1145–61.

36. de Luca A, Tea I, Robins R, Charles M-A, Hankard R. Des cheveux pour évaluer le métabolisme protéique chez l’homme. Cah Nutr Diététique. 2013;48(2):86–91.

37. Castilla-Serna L, Pérez-Ortiz B, Cravioto J. Patterns of muscle and fat mass repair during recovery from advanced infantile protein-energy malnutrition. Eur J Clin Nutr. 1996 Jun;50(6):392–7.

38. Bartz S, Mody A, Hornik C, Bain J, Muehlbauer M, Kiyimba T, et al. Severe Acute Malnutrition in Childhood: Hormonal and Metabolic Status at Presentation, Response to Treatment, and Predictors of Mortality. J Clin Endocrinol Metab. 2014;99(6):2128–37.

39. Neuberger FM, Jopp E, Graw M, Püschel K, Grupe G. Signs of malnutrition and starvation—Reconstruction of nutritional life histories by serial isotopic analyses of hair. Forensic Sci Int. 2013;226(1–3):22–32.

40. Hatch K, Crawford M, AW K, Thomsen S, Eggett D, Nelson S, et al. An objective means of diagnosing anorexia nervosa and bulimia nervosa using 15N/14N and 13C/12C ratios in hair. Rapid Commun Mass Spectrom. 2006;20:3567–77.

41. Mekota A-M, Grupe G, Ufer S, Kunz U. Serial analysis of stable nitrogen and carbon isotopes in hair: monitoring starvation and recovery phases of patients suffering from anorexia nervosa. Rapid Commun Mass Spectrom. 2006;20:1604–10.

42. Mekota A-M, Grupe G, Ufer S, Cuntz U. Identifying starvation episodes using stable isotopes in hair. Rechtsmedizin. 2009;19(6):431–40.

43. Petzke KJ, Fuller B, Metges C. Advances in natural stable isotope ratio analysis of human hair to determine nutritional and metabolic status. Curr Opin Clin Nutr Metab Care. 2010;13(5):532–40.

44. D’Ortenzio L, Brickley M, Schwarcz H, Prowse T. You are not what you eat during physiological stress: Isotopic evaluation of human hair. Am J Phys Anthropol. 2015;157(3):374–88.

45. Jackson JM, Blaine D, Powell-Tuck J, Korbonits M, Carey A, Elia M. Macro- and micronutrient losses and nutritional status resulting from 44 days of total fasting in a non-obese man. Nutr Burbank Los Angel Cty Calif. 2006 Sep;22(9):889–97.

46. Thuo N, Ohuma E, Karisa J, Talbert A, Berkley JA, Maitland K. The prognostic value of dipstick urinalysis in children admitted to hospital with severe malnutrition. Arch Dis Child. 2010 Jun;95(6):422–6.

47. Julious SA. Tutorial in biostatistics: Sample sizes for clinical trials with Normal data. Vol. 23, Statistics in Medicine. 2004. p. 1921–86.

48. Gibson RS, Ferguson EL. An interactive 24-hour recall for assessing the adequacy of iron and zinc intakes in developing countries. Washington DC: HarvestPlus; 2008.

49. FAO Nutrition and Consumer Protection Division. Guidelines for measuring household and individual dietary diversity. 2008. 1–21 p.

50. FAO, IFAD. Guidelines for estimating the month and year of birth of young children. Rome; 2008.

51. WHO. Training course on child growth assessment - Measuring a child’s growth. Vol. 7. 2008.

52. Lukaski HC, Johnson PE, Bolonchuk WW, Lykken GI. Assessment of fat-free mass using bioelectrical impedance measurements of the human body. Am J Clin Nutr. 1985 Apr;41(4):810–7.

53. Tomkins A. Assessing micronutrient status in the presence of inflammation. J Nutr. 2003;133(5 Suppl 2):1649S-1655S.

54. Thurnham DI, McCabe GP, Northrop-Clewes CA, Nestel P. Effects of subclinical infection on plasma retinol concentrations and assessment of prevalence of vitamin a deficiency: Meta-analysis. Vol. 362, Lancet. 2003. p. 2052–8.

55. Thurnham DI, McCabe LD, Haldar S, Wieringa FT, Northrop-Clewes CA, McCabe GP. Adjusting plasma ferritin concentrations to remove the effects of subclinical inflammation in the assessment of iron deficiency: A meta-analysis. Am J Clin Nutr. 2010;92(3):546–55.

56. Erhardt JG, Estes JE, Pfeiffer CM, Biesalski HK, Craft NE. Combined measurement of ferritin, soluble transferrin receptor, retinol binding protein, and C-reactive protein by an inexpensive, sensitive, and simple sandwich enzyme-linked immunosorbent assay technique. J Nutr. 2004;134(11):3127–32.

57. Erhardt JG. Measurement of the vitamin A and iron status in blood [Internet]. [cited 2015 May 27]. Available from: http://www.nutrisurvey.de/blood_samples/index.htm

58. UNAIDS. HIV and AIDS estimates. [Internet]. Available from: http://www.unaids.org/en/regionscountries/countries/burkinafaso

59. Howie SRC. Blood sample volumes in child health research: review of safe limits. Bull World Health Organ. 2011;89(1):46–53.

# List of annexes

Annex 1 Information to participants

Annex 2 Consent form

Annex 3 Chronogram

Annex 4 Field team composition

Annex 5 Project governance

Annex 6 Signatures

Annex 7 Optidiag study

## Annex 1 Information to participants

**Action Contre la Faim**

BP 10 221 Ouaga 06

Rue 13-22, Quartier Zogona (Zone du Bois) - Porte 557

Ouagadougou, Burkina Faso

Tel (reception):+226 25 36 98 30

**Study contact:**

**BAGAYA Fatimata**: 64 18 00 24

**ZONGO Barnabé**: 66 39 79 46

**INFORMATION LETTER**

**PROJECT TITLE**

*Efficacy of an optimized RUTF dose (“Plumpy Nut”) on the recovery of uncomplicated severe acute malnutrition in children aged 6 to 59 months*

Before you decide whether or not to participate in this research, it is important that you understand why the research is being done and what it will involve. Please take time to read this information letter carefully and discuss it with others if you wish. Ask us if there is anything that is not clear or if you would like more information. Take time to decide whether or not you wish to take part. Thank you for reading this.

**WHY HAVE I BEEN ASKED TO PARTICIPATE?**

We are asking you whether you and your child would like to participate because you have a child between 6 and 59 months who has been diagnosed with severe acute malnutrition and has the right to receive nutritional treatment in the form of a fortified peanut butter to take home.

**WHAT IS THE PURPOSE OF THE RESEARCH?**

The focus of this study is to investigate if different amounts of ready-to-use therapeutic foods (RUTF), in this case Plumpy Nut are equally effective for treatment of malnutrition. One group will receive the normal ration of RUTF whereas the second group will receive the same product, but after the first critical weeks a slightly lower ration. As with medicine, there is no need to give higher doses than what is adequate for recovery from illness. If treatment is followed properly, the reduced ration should still be sufficient for your child to recover from severe acute malnutrition (SAM).

**CAN I CHOOSE IF MY CHILD RECEIVES THE STANDARD RATION OR THE REDUCED RATION?**

No, it is very important for the results of the research that participants or the researchers do not choose the ration themselves. It is therefore done by drawing lots. However, the recovery process is followed carefully in all children, and should your child not be gaining weight sufficiently, he or she will be transferred for additional treatment.

**WHAT WILL HAPPEN TO ME AND MY CHILD IF WE PARTICIPATE?**

If you agree on behalf of your child to participate in the research, you will be asked some questions about your child, yourself and your family.

If it has not already been done, the appetite of your child will be checked through an observation in which s/he is offered a small quantity of Plumpy Nut. If your child likes the RUTF and is able to consume the dose, s/he will be assigned to one of the two groups (high or low dose). This allocation is already decided in advance and depends solely on the ID number you will be given, and you cannot choose which group you prefer your child to belong to. Your child will receive all necessary treatment and there is no difference in the care provided between the groups apart from the amount of food given.

You will be asked to follow the treatment every day and return on a fixed day every week for monitoring of your child’s recovery and to receive the treatment food.

Every week, when you come to the health centre, your child will be seen by the research team. They will measure weight, height, arm circumference, and assess for signs of oedema. In addition, the team will ask you questions regarding the health of the child and examine your child for diseases.

On the first and last day of the treatment, a blood sample (2.5 ml as the small tube the nurse has shown you) will be taken from your child. We will use small sterile disposable needles specifically for children. This blood will be used to measure if your child has malaria, anaemia, iron deficiency or vitamin A deficiency. If your child has malaria or anaemia, we will share the information immediately with you and your child will get treatment if necessary.

We also wish to measure the quantity of water in your child’s body and how it is distributed because it is an important marker of health. We can do this by passing a very weak (undetectable) electric current between two electrodes placed in the hand and in the foot of the child while they are lying down. This test is completely harmless and cannot be sensed and only takes some seconds. This test will also be conducted at admission and at the end of treatment.

After your child has recovered from the malnutrition, you will be asked to return to the health centre every 2 weeks for 3 months after your child was discharged. The research team will measure your child’s weight, height, arm circumference and possible oedema. When you come, we will ask you about the child’s wellbeing, and about other foods he/she eats.

We will visit you at home in case your child is absent to understand the reason for your absence.

**ARE THERE ANY BENEFITS OR RISKS TO PARTICIPATE?**

The main benefit of participating to the study is that your child will receive treatment for malnutrition, as in normal service by health centre.

There are no other direct benefits from taking part in this research except that your child will be followed a bit closer by the research team than in routine treatment. However we hope that the information from you and your peers can be used to develop an improved treatment of malnutrition that would benefit all children with severe acute malnutrition in the future.

If your child receives the smaller amount of RUTFs/he could gain weight less fast. However, your child will be checked every week and if weight gain is not enough or s/he is losing weight, we will do several things, as per routine protocol and for all children. At first, we will see if your child is sick and make sure s/he receives treatment for any other illness. We will also find out how much RUTFs/he is really eating. You will receive advice from us on how to make sure s/he gains enough weight to recover. If s/he is not gaining weight, we will organise for your child to receive more attention by the health team to find possible reasons for this. In case your child presents danger signs such as high fever, vomiting, diarrhea, etc., he/she will be referred to the hospital for inpatient treatment, as per routine protocol.

**WILL MY PARTICIPATION IN THE RESEARCH BE KEPT CONFIDENTIAL?**

All information which is collected about you, your child and your family during the course of the research will be kept strictly confidential. Apart from the consent form, your name and details will be removed from any other information you supply to us before we analyse the data. The consent forms will be kept separately from information about your child, yourself and your family. We will only take photos of you and your child if you agree, and we will only use them for teaching and presentation if you allow us to.

**DO I HAVE TO PARTICIPATE?**

It is up to you to decide whether or not to take part. If you decide to take part, you are still free to withdraw at any time and without giving a reason. If you do decide to take part you will be given this information sheet to keep and be asked to sign a consent form. If you decide to take part you are still free to withdraw at any time and without giving a reason.

If you decide not to take part to the study from the beginning or to stop participating after a certain time, your child will still receive treatment for his illness through the normal nutrition program. The standard treatment and care of your child will continue even if you decline to participate or decide to stop the study at any point of time.

**WHAT WILL HAPPEN IN THE CASE OF INJURY?**

If your child is injured or if you have questions about any unexpected injuries as a result of taking part in this study, please contact a study nurse at the health centre. The study is covered by an insurance and the study team will ensure the management and indemnification of any adverse effect observed on the child and that should arise from using the nutritional product of from participating to this study, during the study period.

**HAS THE STUDY BEEN OFFICIALLY APPROVED?**

The national ethical committee (Comité national d'éthique pour la recherche en santé (CNERS)) in Burkina Faso had reviewed and approved the current study protocol. The study is funded by Action Against Hunger and several other research donors such as European Union and the Children’s Investment Fund Foundation.

**WHO CAN I CONTACT IF I HAVE A QUESTION OR COMPLAINT?**

Complaints or questions can be addressed to any member of the study team at the health centre. Please do not hesitate to ask questions, small or large. We will do our best to answer your questions.

Receive our sincere greetings

Thank you

The research team

## Annex 2 Consent form

Study ID: _________

**CONSENT FORM**

**PROJECT TITLE**

*Efficacy of an optimized RUTF dose (Plumpy Nut) on the recovery of uncomplicated severe acute malnutrition in children aged 6 to 59 months*

**YES NO**

I confirm that I have read and understood the information sheet for the above
study and have had opportunity to ask questions

I understand that participation is voluntary and that I am free to withdraw
participation at any time, without giving any reason, and that this will not affect my

child’s rights to treatment in any way

I understand that I and my child are not entitled to receive any special treatment,

payment or gifts in return for participating to the study

I agree, on behalf of my own, or my custody child, to take part in the study

**USE OF PHOTOS**I agree, that photos or videos taken of my child and/or me can be used for training or

presentation purpose at a conference

**NAME AND SIGNATURE OF PARTICIPANT**

_____________________

(Name of child)

_____________________ ___________ __________________

(Name of caregiver) (Date) (Signature or thumbprint)

**NAME AND SIGNATURE OF RESEARCH STAFF**

___________________ ___________ __________________

(Name of ACF staff) (Date) (Signature)

## Annex 3 Chronogram


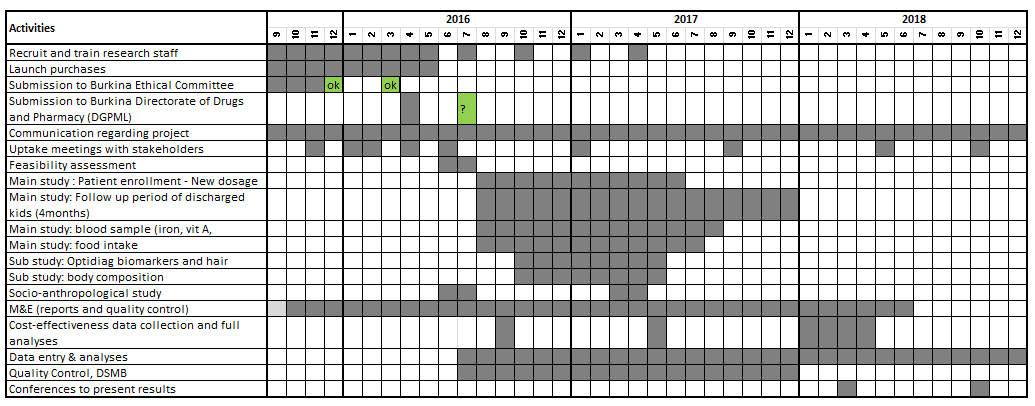


## Annex 4 Field team composition

| Position | Activity | Nb |
| --- | --- | --- |
| OFFICE TEAM | | |
| Head of Research Project (HoP) | To lead the field implementation of the entire project  Contact person with local institutions & external collaborators  Ensure the respect of the scientific protocol during the field implementation  Data analysis and cleaning  Ensure ethical considerations are respected | 1 |
| Assistant HoP | Supervision of two research teams  Ensure the research protocol is respected via the application of the standard operating procedures  Assist in data collection and project monitoring | 1 |
| Data Manager | Extract the data collected in the field and ensure its daily upload on the server  Ensure the quality control of the data  Analyze the data and construct tables and graphs representing the data  Ensure the confidentiality and archiving of the data at the end of the project | 1 |
| Communication Officer | Ensure a good internal and external communication of the project  To develop communication tools regarding the project  Coordinate the communication activities of the project | 1 |
| Lab technician | Treat the blood samples  Store the serum samples  Maintenance of the lab materials and equipments | 1 |
| CEA translator | FR-EN translator (1month only) able to speak local languages also best |  |
| CEA surveyor | To conduct focus group discussion and/or interviews for CEA (1month only) |  |
| *SUBTOTAL FOR OFFICE STAFF* | | **5** |
| TWO FIELD TEAMS | | |
| Team supervisors | Supervise the team and ensure backup of absent members  Key contact person for field teams and for routine staff at health centers  Ensure data collection and routine checks on data quality  Enrol patients and register their consent to participate | 2 |
| Clinical Nurse | Ensure registration and follow up of participants on the OTP cards  Conduct medical checks of children & the clinical decisions  Conduct blood sampling | 2 |
| Measurers | Administer the research questionnaires  Take anthropometric measurements at admission and at each weekly visits  Mobilise, form and sensitise beneficiaries on the project and the good hygienic, dietary and care practices  In collaboration with the community health workers, ensures the tracing of children absent from follow up visits | 6 |
| Distributor | Administer the appetit tests  Administer the weekly adherence questionnaires  Distribution of product according to random sequence | 2 |
| Driver car | to bring team from 1 site to the other | 2 |
| *SUBTOTAL FOR FIELD TEAMS* | | ***14*** |
| TOTAL | | **19** |

## Annex 5 Project governance

|  | Name | Contact information |
| --- | --- | --- |
| Principal Investigator: | Suvi Kangas | Head of Research Project - MANGO  Action contre la Faim – France, mission in Burkina Faso  06 BP 10221 Ouagadougou  Tél : +226 77 69 97 16  [rpmango-fa@bf.missions-acf.org](mailto:rpmango-fa@bf.missions-acf.org) |
| Co-Investigateur : | Cécile Salpéteur | Nutrition Research Project Coordinator  Action Contre la Faim - France  14-16 Boulevard Douaumont – CS 80060  FR-75854 PARIS CEDEX 17  Tél. : +33 (0)1.70.84.73.49  [csalpeteur@actioncontrelafaim.org](mailto:csalpeteur@actioncontrelafaim.org) |
| Scientific partners: | Henrik Friis, MD, PhD University of Copenhagen | Professor of International Nutrition and Health, Dept Nutrition, Exercise and Sports, University of Copenhagen  Rolighedsvej 30  DK-1958 Frederiksberg  Tel: +45 35 33 38 60  [hfr@nexs.ku.dk](mailto:hfr@nexs.ku.dk) |
|  | Jonathan Wells, PhD, University College of London | Professor of Anthropology and Pediatric Nutrition  Childhood Nutrition Research Centre  UCL Institute of Child Health  30 Guilford Street  London WC1N 1EH  [jonathan.wells@ucl.ac.uk](mailto:jonathan.wells@ucl.ac.uk) |
|  | Carlos Navarro Colorado, MD, MSc, PhD  Center for Disease Control and prevention – Atlanta, USA | Emergency Response and Recovery Branch / Division of Global Disease Protection / Center for Global Health, US Centers for Disease Control and Prevention  1600 Clifton Road  Atlanta, GA, 30333, USA  [CNavarroColorado@cdc.gov](mailto:CNavarroColorado@cdc.gov) |
| Steering committee | André Briend, MD | Independent expert  [andre.briend@gmail.com](mailto:andre.briend@gmail.com) |
|  | Henrik Friis, MD, PhD | Professor of International Nutrition and Health, Dept Nutrition, Exercise and Sports, University of Copenhagen  Rolighedsvej 30  DK-1958 Frederiksberg  Tel: +45 35 33 38 60  [hfr@nexs.ku.dk](mailto:hfr@nexs.ku.dk) |
|  | Anne-Dominique Israel-de Monval | Senior Nutrition and health advisor  Action Contre la Faim - France  14/16 Boulevard Douaumont - CS 80060  FR- 75854 PARIS CEDEX 17  Tel: +33.(0)1.70.84.70.22  [adi@actioncontrelafaim.org](mailto:adi@actioncontrelafaim.org) |
|  | Myriam Aït-Aissa | Senior Research Coordinator  Action Contre la Faim - France  14/16 Boulevard Douaumont - CS 80060  FR- 75854 PARIS CEDEX 17  Tel: +33.(0)1.70.84.70.58  [maitaissa@actioncontrelafaim.org](mailto:adi@actioncontrelafaim.org) |
|  | Elise Rodriguez | Responsible Advocacy  Action Contre la Faim - France  14/16 Boulevard Douaumont - CS 80060  FR- 75854 PARIS CEDEX 17  Tel: +33.(0)1.70.84.73.81  [eliserodriguez@actioncontrelafaim.org](mailto:eliserodriguez@actioncontrelafaim.org) |
|  | Alexandre le Cuziat | Directeur Régional des Opérations  Action Contre la Faim - France  14/16 Boulevard Douaumont - CS 80060  FR- 75854 PARIS CEDEX 17  Tel: + 33 (0) 1 70 84 70 08  [alecuziat@actioncontrelafaim.org](mailto:alecuziat@actioncontrelafaim.org) |
| Working group | Suvi Kangas | Head of Research Project - MANGO  Action contre la Faim – France mission in Burkina Faso  06 BP 10221 Ouagadougou  Tél : +226 77 69 97 16  [rpmango-fa@bf.missions-acf.org](mailto:rpmango-fa@bf.missions-acf.org) |
|  | Cécile Salpéteur | Nutrition Research Project Coordinator  Action Contre la Faim - France  14-16 Boulevard Douaumont – CS 80060  FR-75854 PARIS CEDEX 17  Tel: +33 (0)1.70.84.73.49  [csalpeteur@actioncontrelafaim.org](mailto:csalpeteur@actioncontrelafaim.org) |
|  | Pernille Kaestel | Consultant  [Pernille.kaestel@gmail.com](mailto:Pernille.kaestel@gmail.com) |
|  | Olivia Freire | Operational Technical Advisor in Nutrition  Action Contre la Faim - France  14-16 Boulevard Douaumont – CS 80060, 75854 PARIS CEDEX 17  Tel: +33 (0)1.70.84.70.14  [ofreire@actioncontrelafaim.org](mailto:ofreire@actioncontrelafaim.org) |
|  | Abdulaziz Biga Hassoumi | Head of Nutrition Department  ACF-Burkina Faso  Tel : +226 75 24 65 35  [cmn@bf.missions-acf.org](mailto:cmn@bf.missions-acf.org) |
|  | Carlos Navarro Colorado, MD, MSc, PhD  Center for Disease Control and prevention – Atlanta, USA | Emergency Response and Recovery Branch / Division of Global Disease Protection / Center for Global Health, US Centers for Disease Control and Prevention  1600 Clifton Road  Atlanta, GA, 30333, USA  [CNavarroColorado@cdc.gov](mailto:CNavarroColorado@cdc.gov) |

## Annex 6 Signatures

|  | Name, Date & Signature |
| --- | --- |
| **Alexandre le Cuziat**  Regional Director of Operations  Adequation of project with operational strategy in selected country. Adequation of proposed resources /objectives on the mission. |  |
| **Anne-Dominique Israel-de Monval**  Senior Advisor for Nutrition & Health Sector :  Adequation of the project with technical strategy |  |
| **Myriam Aït-Aissa**  Senior Advisor for Research:  Adequation of the project with research strategy  Adequation of proposed resources /objectives |  |
| **Henrik Friis**  Scientific partners |  |
| **Elise Rodriguez**  Director of Expertise & Advocacy Department |  |

## Annex 7 Optidiag study

🞂 CONTEXT

In 2012, 19 million children worldwide were suffering from SAM, most of them living in sub-Saharan Africa and Southeast Asia. SAM children have higher risk of mortality (relative risk between 5 and 20). It is an underlying factor in over 50% of the 10-11 million preventable deaths per year among children under 5. At present, 65 countries have implemented outpatient treatment for (SAM) but the programs have very low coverage, reaching only around 10-15% of SAM children.

WHO recommends two anthropometric criteria for SAM diagnosis and monitoring in children aged 6-59months: WHZ<-3 or MUAC<115mm (WHO 2009, 2013). There is however a significant and sometimes huge discrepancy between these two criteria: they do not usually identify the same children as suffering from acute malnutrition and display different levels of gravity of the nutritional situation in the population.

In 2009, WHO estimated that only about 40% of the children selected by one criterion were also selected by the other (WHO 2009). There are in fact huge variations across contexts, and extreme discrepancies are frequently reported. A recent study in Cambodia concluded that MUAC<115 mm and WHZ<-3 identify a different set of children with malnutrition, with hardly any overlap between the 2 indicators (Laillou 2014). Most of the time, caseloads defined by WHZ are much larger than by MUAC (Dasgupta 2013, Laillou 2014, Myatt 2009, Roberfroid 2013, ACF survey datasets), but the contrary may happen as well, especially in the younger age groups, as evidenced in recent surveys conducted by ACF in Sierra Leone and Madagascar.

A major challenge is thus to investigate the possible variations in the needs of undernourished children identified by different diagnostic measures and thereby to ensure optimized identification and treatment of SAM children.

Besides, MUAC and WHZ are imperfect and inadequate markers of the risks of long-term morbidity (stunting and wasting) and mortality in malnourished children. Despite advances in treatment, the mortality rates among SAM children also remain staggeringly high (Buttha 2013, Kerac 2014). Current diagnostic criteria lack specificity and sensitivity for mortality and have limited utility for guiding treatment or for predicting long-term outcomes such as lack of weight gain, growth failure (“stunting”). Improvements in diagnosis tools and criteria are therefore highly needed to identify SAM children at highest risk of disability and death.

While the signification of current anthropometric diagnostic heterogeneity was initially pointed out by WHO as requiring more investigation, very little has been done so far.

The question of possible diagnostic errors has already been raised by several experts. Current hypothesis of errors are that WHZ overestimates the diagnosis of acute malnutrition in populations with a slender morphology (i.e. with a low sitting-to-standing-height ratio; SSR) as observed in pastoralists (Myatt 2009), and that MUAC at a fixed cut-off underestimates acute malnutrition in older, male, and non-stunted children (Roberfroid 2013). Several recent reviews highlight the fact that we are essentially comparing two different methods and that we lack a gold standard.

WHZ and MUAC also may identify a separate kind of physiological deficit. It has been hypothesized that this might be related to differing impairments of fat and muscle mass stores, with MUAC reflecting preferentially fat mass for some authors (Chomto 2006) and muscle mass for others (Brambilla 2000). Children identified by different criteria may thus require different treatments. For instance, lower anthropometric response to treatment (lower MUAC gain and weight gain, longer treatment duration and higher proportion of non-responders) has already been observed in younger and stunted female children (Bekele 2009), three characteristics which are independently associated with MUAC diagnosis (WHO 2009, Berkley 2005, Shams 2012). These factors may influence the underlying pathophysiology of SAM and also the treatment efficacy of nutritional rehabilitation. Also, a recent meta-analysis of follow-up datasets evidenced a dramatic increase in mortality risk in children combining low WHZ and stunting (MUAC was not factored in) (McDonald 2013).

All reviews of available evidence on this issue highlighted the need for robust research (EN-net 2012; Roberfroid 2013) to further investigate the physiological significance of the different anthropometric criteria and to better understand how the clinical status and nutritional needs of the children are addressed over the course of nutritional rehabilitation.

In particular, solid evidence is needed on the risks of children with a WHZ<-3 but a MUAC above 115mm. Also, the extent to which all children with MUAC<115 mm require standard nutritional rehabilitation must be clarified. Finally, the extent to which a combination of stunting and wasting affects children and influences their response to SAM treatment needs to be explored.

In the meanwhile, reliance on these SAM anthropometric criteria and rationalization of the access to treatment to one of them may in fact lead to misappropriation of critical resources and may prevent identification and treatment of high-risk children. However, MUAC, since its introduction by WHO in 2007, is more and more used in practice as the only admission criterion for medical-nutritional rehabilitation programs (Roberfroid 2013), while the signification of the heterogeneity in diagnosis is still unknown. This is causing ethical and technical problems today, especially in emergencies and other contexts, where the MUAC criterion, which is preferred to WHZ for practical reasons, is increasingly used as a standalone criterion for rapid assessments and nutrition programming.

**The need for this evidence is particularly well acknowledged by ACF field missions. For instance, in Bangladesh, national guidelines are only considering low MUAC as an admission criterion for uncomplicated SAM management, which is de facto excluding the vast majority of the SAM children, those who have MUAC≥115mm. There is thus a crucial need to confirm or infirm existing policy.**

The proposed research aims, through the production of evidence from a variety of geographical and ethnical contexts, at addressing these knowledge gaps on:

• The reality and diversity of the needs associated with different types of anthropometric deficits;

• The adequacy of current medico-nutritional programs to meet the needs of these different types of patients, restoring healthy growth over time and reducing risk of mortality.

ACF France and ITM Antwerp have undertaken an analysis of a variety of ACF datasets (from surveys and programs) to describe and understand the degree of discrepancy between MUAC and WHZ in the identification of malnourished children. The proposed research project directly builds on this work, which has already been presented (posters) in several congresses and whose publications are being finalized. Useful to check and formulate hypothesis, retrospective data are limited in providing sound evidence on what group of children needs to be treated in priority.

Answering these questions will contribute to evidence-based policies and practices for the identification of individuals who can benefit most from standard treatment.

🞂 GENERAL OBJECTIVE

To generate new evidence on pathophysiological process, nutritional needs and risks associated with different types of anthropometric deficits in children under 5, in order to optimize the diagnosis and treatment of SAM.

🞂 SPECIFIC OBJECTIVES

- To compare nutritional status, metabolism, pathophysiological process and risks in different types of SAM anthropometric diagnosis, with or without concomitant stunting.
- To analyze the extent to which current SAM treatment is promoting recovery and healthy growth in different categories of children.
- To evaluate the relevance of current discharge criteria used in nutrition programs and their association with metabolic recovery, in different age groups and among those who are stunted.
- To test novel rapid tests of emerging biomarkers predicting long-term outcomes and mortality risk in the field
- To investigate all this in a variety of contexts in order to evaluate the possible effect of ethnical and geographical variability

🞂 METHODOLOGY

**General considerations**

A wide range of supplementary information related to nutritional status, body composition, metabolic and immune status, including emerging biomarkers of metabolic deprivation and vulnerability, will be collected in addition to anthropometric measurements in observational studies.

These parameters and studies are selected for their relevance as well as their practicality of implementation, so that the study procedures fit within the routine activities of the nutrition rehabilitation programs, and will not necessitate implementing interventions which do not already exist.

The reason for this approach is that there is no gold standard to date to define SAM or describe its severity, thus a large number of indicators and biomarkers are necessary to generate valuable evidence.

Also we want to collect this information in remote health centers, so that the results obtained will be valuable for all SAM cases and not only for the few complicated and very severe cases reaching the nutrition ward of the hospital.

Finally, we want to be able to collect this information in a variety of contexts.

The studies will be run in three different countries: Burkina Faso (Gourma district), Bangladesh (Cox Bazaar district) and Indonesia (Kupang district).

**OptiDiag Indicators**

The supplementary indicators which will be assessed besides demographic characteristics (age, gender, region), usual anthropometric measurements (weight, height, MUAC), health status, and socioeconomic indicators (number of siblings, family income, access to clean water etc) are:

• caretaker’s statement on child’s health status (weight changes, food intake, observed morbidity)

• biomarkers of micronutrient deficiencies (e.g. Iron status biomarkers like serum ferritin/ serum transferrin receptor; Vit A status biomarkers like retinol binding protein; Vit C in the urine)

• biomarkers and indicators of body composition and energy metabolism (urinary ketones/natural enrichment of nitrogen and carbon stable isotopes in hairs/ circulating leptin and adiponectin)

• biomarkers of non-specific immune response or urinary infections (C-reactive protein level/urinary nitrites/urinary leucocytes esterase)

Indeed, several studies proved that the variation of δ15N and δ13C (Nitrogen and Carbon stable isotopes ratios) in the hair can be used for tracing the temporal evolution of nutritional status (Hatch 2006, Mekota 2006, 2009, DeLuca 2010, Petze, Neuberger 2013). Sections of hair synthesised during food deprivation with decreasing body mass index (BMI) were indeed shown to contain increased δ15N and decreased δ13C, indicating catabolism of body protein and fat stores. Since keratin remains unchanged after synthesis, and the speed of hair growth is constant (around 2.5mm/week), the information on protein-energy metabolism week after week can be traced back along the hair, thereby indicating not only the severity of the episode of wasting but also the metabolic effects of the nutritional rehabilitation (on both lipid and protein anabolism).

Recently the serum concentration of C-reactive protein was shown to be associated with risk of mortality in SAM children (Page 2014). CRP as well as biomarkers of Iron, Vit A status can be assessed in a few drops of capillary blood (Erhardt 2004).

Finally, in recent studies, Dr. Freemark and colleagues used metabolomic analysis and micro-assays to characterize the metabolic status of malnourished Ugandan infants and young SAM children (Mody 2014, Bartz 2014), and identified hormonal and metabolic factors at presentation that predicted mortality during treatment. The baseline factors most closely associated with mortality were two hormones produced by white adipose tissue: leptin and adiponectin.

**Study design**

This proposal includes two cross-sectional surveys involving representative samples of children between 6 months and 5 years old (approximately 700 by survey) and three prospective follow-up studies involving cohorts of SAM children between 6 months and 5 years old (600 in Burkina, 400 in Bangladesh and 200 in Indonesia). Surveys in representative samples are useful to check how alternative indicators of metabolic and nutritional impairments are distributed in the population, check their coherence (are we able to identify cases vs non-cases?), and finally their diagnosis performance (sensitivity specificity for cases) compared with anthropometric indicators. On another hand, SAM children cohort studies are necessary to compare the current types of anthropometric diagnosis of SAM, through alternative indicators, check the prognosis value of alternative indicators, and finally to see how far the metabolic and nutritional status is restored by current SAM management.

The cross-sectional surveys will be nested into SMART surveys.

The cohort studies will be nested into SAM management programs run with the technical support of ACF-France, in hospitals as well as healthcare centers involved in SAM management. Children admitted as SAM at the nutrition centers will be enrolled into the cohort. The follow up duration for each child enrolled will be at least three months. The duration of each cohort study will be around one year.

In both types of studies, the activities, besides the regular reporting of the data collected, will mainly consist in formal patient recruitment procedure (informed consent), in biological samples collection and rapid tests, and in the shipment of samples to France or Germany, as detailed below.

A single collection of hair sample from SAM children at recruitment (SMART survey) or at the end of the treatment (cohort study) will be required to characterize the nature and the magnitude of nutritional deficits (in terms of duration and severity) at admission, and also the efficacy of treatment (only for the cohort study). Then the samples will be sent to AgroParisTech lab in Paris, where different sections of the hair will be analyzed for δ15N and δ13C through EA-IRM analysis, as described elsewhere (Neuberger 2013)

Leptin and adiponectin will be assessed at admission and follow-up time through novel Point-Of-Care assays currently developed by Pr Freemark at Duke University Medical Center. This rapid test relies on the collection of a single finger-prick drop of blood.

Small serum samples from few finger-prick drops of blood will be collected at admission and follow-up time, frozen, and sent for analysis of micronutrient deficiencies and immune response biomarkers in Germany (VitMin Lab).

In the older children (above two years old), a quick assessment of different biological parameters in urine can be done through the collection of non-sterile urine samples and then the use of urine test stripes with multiple indicators (Roche or Combi Screen, Analyticon).

**HR role and responsabilities**

This project is based on a strong and established partnership between ACF-France and three key academic partners: AgroParisTech University, Institute of Tropical Medicine of Antwerp, and Duke University Medical Center. ACF will be in charge of the global coordination of the project, of the recruitment, training and supervision of the staff implementing the studies protocols at field level, in collaboration with day-to day program staff, and of the coordination with authorities and local health services providers. Pr Patrick Kolsteren, head of Child health Unit at ITM Antwerp, who has a strong expertise in epidemiology and public health issues related to undernutrition, and a long-lasting experience of conducting research projects in humanitarian context, in collaboration with national academic partners, will be the principal Investigator. He will provide a scientific support of the whole project, on study designs and on data analysis, and will have a strong role in the interpretation and dissemination of the results. The team of Pr Jean-François Huneau at AgroParisTech has an expertise in the assessment of human energy and protein metabolism. They will be in charge of the analysis of stable isotope ratio in hair samples and contribution significantly to the interpretation and dissemination of the results. Finally, the team of Pr. Michael Freemark at Duke University medical School will provide assistance in the development and design of the studies and point-of-care assays of metabolic biomarkers.

This project will form the basis of an academic PhD project, conducted by the Project Manager hired by ACF, under the academic co-supervision of Jean-François Huneau (AgroParisTech) and Patrick Kolsteren (ITM Antwerp), under the hierarchical management of ACF Nutrition and Health Research Coordinator Benjamin Guesdon.

There will thus be a scientific coordinating body made up of ITM, AgroParisTech and ACF research staff.

In each study site, a national research manager and the supplementary medical staff, possibly including staff from a national academic partner, will be recruited and specifically dedicated to research activities. There will be one supplementary medical staff by survey team in the SMART survey, and two supplementary medical staff by center at admission/visit time in the cohort study. After training by the OptiDiag Project Manager (PhD student), they will support the day-to-day programme staff in the implementation of the children’s recruitment procedure and in the implementation of the sample collection protocols. The OptiDiag Project Manager, the Research Coordinator and external academic partners will support the set-up and implementation of the studies by both remote and in-country (through regular visits) supervision.

The monitoring will be ensured by continuous data collection and analysis by the HQ-based research staff and by academic partners. Data on the number of children recruited and on the different parameters collected will thus be shared on a monthly basis by the field-based research staff. Shipment of the samples will be regularly organized, depending on the storage capacities at ACF mission level.

🞂 EXPECTED OUTCOMES

- Confirmation of current hypotheses related to:

a) possible misdiagnosis of SAM made by MUAC or WHZ criteria,

b) varying degree of severity and need for admission to treatment of the different types of diagnosis,

c) underlying heterogeneity of the pathophysiology.

- Generation of new algorithms for the assessment and classification of malnourished children, based on the combined use of emerging biomarkers and anthropometric measures, or on the modification of anthropometric criteria.
- Generation of new treatment paradigms based on the predictive value of biomarkers in combination with traditional anthropometric measures. This will enable us to assess the power of current treatment regimens to promote long-term weight gain and growth and will allow us to tailor treatment to the physiological needs of the child.

🞂 Contacts

- **ACF-France:**

Dr. Benjamin Guesdon, Nutrition and Health Research Coordinator,

[bguesdon@actioncontrelafaim.org](mailto:bguesdon@actioncontrelafaim.org)

- **Institute of Tropical Medicine of Antwerp :**

Pr. Dr. Patrick Kolsteren, Head of Child Nutrition and Health Unit, Institute of Tropical Medicine of Antwerp, Belgium,

[PKolsteren@itg.be](mailto:PKolsteren@itg.be)

- **AgroParisTech University:**

Pr. Dr. Jean-François Huneau, Department of life Science & Health, Division of Human Nutrition, [jean_francois.huneau@agroparistech.fr](mailto:jean_francois.huneau@agroparistech.fr)

- **Duke University Medical Center:**

Pr. Dr. Michael Freemark, Chief, Division of Pediatric Endocrinology and Diabetes, Vice Chair for Pediatric Research,

[michael.freemark@duke.edu](mailto:michael.freemark@duke.edu)
