# Supplementary material for: Impact of reduced dose of ready-to-use therapeutic foods in children with uncomplicated severe acute malnutrition: A randomised non-inferiority trial in Burkina Faso
Source: PLoS Med. 2019 Aug 27;16(8):e1002887. doi: 10.1371/journal.pmed.1002887 (PMC6711495; doi:10.1371/journal.pmed.1002887)
Supplement: S3 Text — (DOCX) [file pmed.1002887.s006.docx]

Statistical Analysis Plan

Study title :

**MANGO**

= Modelling an Alternative Nutrition Protocol

Generalizable for Outpatient care

| **Version** | **Date** | **Update concerning** | **Clarification to update** |
| --- | --- | --- | --- |
| 1 | 10/8/2018 |  |  |
| 2 | 30/1/2019 | 1) False discharge  2) Per protocol (PP) definition | 1) Adding a false discharge category for those children that were falsely discharged as recovered or referred with weight loss or stagnant weight where they should have not been.  2) Previously: PP defined as recovered, non-response or deceased  Now: PP defined only via adherence & respect of protocol with 1) acceptable intake of RUTF, 2) no missed visits, 3) no false discharge, 4) receiving the right RUTF dose |
| 3 | 15/6/2019 | Interaction terms & outcomes tests for interactions | Upon revision of the manuscript submitted to peer review and following the comments received, a revision of the analysis plan was made concerning the interaction terms included in the analysis and the outcomes tested for those interactions in order to limit the risk of false positives with multiple testing. In the final analysis plan, only 5 interaction terms were tested for (those pre-specified in the protocol) and only 4 key outcomes were tested for these interactions.  Final interaction terms:   1. Sex 2. Age 3. MUAC category at admission 4. WHZ category at admission 5. Stunting status at admission   Outcomes tested for interactions:   1. Weight gain velocity 2. Recovery 3. Length of stay 4. Height gain velocity |

# Introduction

This statistical analysis plan describes the statistical analyses to be carried out for the MANGO study main results. The plan covers the primary outcome (weight gain velocity) and those secondary outcomes that are planned to be published in the same scientific article including anthropometric and programmatic outcomes. Analysis of body composition data, food and nutrient intake data and micronutrient status data will not be included in this plan.

# Objectives for the main analysis

The main objective of the analysis of the primary outcomes to be covered in the 1^st^ paper is to assess the safety and efficacy of the reduced dosage of RUTF in the treatment of uncomplicated SAM.

Primary outcome:

1. **weight gain velocity** (total and after 1^st^ 2 weeks), comparing the 2 groups

Secondary outcomes:

1. **length of stay** in the nutrition program, comparing the 2 groups
2. **programmatic outcomes**, comparing the recovery, referral, defaulter, non-response, lost-to-follow-up and death rate between the 2 groups
3. **severe adverse events** in terms of differences in referral reasons and illnesses, comparing the 2 groups
4. **linear and MUAC growth** rates during treatment, comparing the 2 groups
5. **relapse rate** after discharge, comparing the 2 groups

# Study design

The MANGO study was designed to investigate the efficacy of a reduced dose of RUTF in the treatment of uncomplicated SAM in a randomised controlled trial setting recruiting children to 2 study arms: 1) intervention group that receives a reduced dose of RUTF from the 3^rd^ treatment week onwards and 2) control group that receives the standard RUTF dose throughout the treatment period. The main outcome to be compared was chosen to be the weight gain velocity of the 2 groups of children from admission to discharge.

The study was designed as a non-inferiority trial with a margin of 0.5g/kg/d to indicate non-inferiority from an estimated mean weight gain velocity of 3.5g/kg/d with a minimum acceptable weight gain velocity of 3.0g/kg/d. The sample size was calculated according to the primary outcome of non-inferiority of weight gain velocity by a margin of 0.5g/kg/d assuming a standard deviation of 2.6g/kg/d as per a preliminary analysis of programmatic data suggested, a power of 80% and a one-sided significance level α=0.05. This calculation gave an estimated sample of 335 children per arm. Accounting for 20% of loss to follow up the target sample size per group was fixed to 400. The study was conducted recruiting patients from 10 different health centres (HCs) by 2 research teams, each operating in 5 HCs. Patients were randomised individually at each HC according to a pre-defined block randomisation code list.

# Hypothesis

Following the non-inferiority design, the null hypothesis (H_0_) of the MANGO study asserts that the reduced dosage is not non-inferior to the standard treatment in terms of weight gain velocity. This includes any result where either the one-sided or the 2-sided 95%CI crosses the 0.5g/kg/d non-inferiority margin. Conversely, the alternative hypothesis (H_1_) of non-inferiority becomes true if the difference in weight gain velocity is less than 0.5g/kg/d including the lower bound of the one-sided 95% confidence interval (see Figure 1).

Non-inferiority will be accepted only if the non-inferiority is proven and the intervention group reaches the minimum acceptable weight gain velocity of 3.0g/kg/d in both PP and ITT analysis (to be defined below). This also applies to sub-groups.


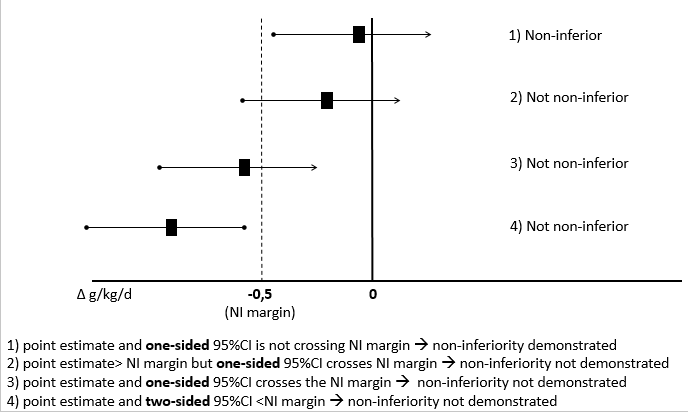


Figure 1: Possible outcomes of the non-inferiority hypothesis

# Intention to treat versus per protocol analysis

As per the recommendations for non-inferiority trials, both intention to treat (ITT) and per protocol (PP) analysis will be performed on the primary outcome of weight gain velocity with ITT being the primary analysis for hypothesis checking. Both ITT and PP will also be done for the variables relating to the primary outcome such as length of stay to provide further details on the possible underlying differences leading to different results in the ITT and PP analysis of the primary outcome. All other outcomes will be analysed on an ITT basis.

The ITT analysis will be done based on all available cases including all randomised subjects who have available outcome data, regardless of possible protocol violation.

The PP analysis will include children whose caregivers declare that they ingested >50% of the RUTF dose throughout the treatment, that had no unscheduled missed visits, that were not falsely discharged and that received the correct RUTF dose throughout treatment.

# Participant flow in the study

Figure 2 shows the format for presenting the flow of children during the four main stages of the trial (enrolment, allocation, follow-up and analysis). Children that present with SAM based on the anthropometric criteria of WHZ<-3 and/or MUAC<115 will be assed for eligibility by the study team and numbers of children excluded for pre-established reasons will be given. Children who are randomised but later discovered non-eligible based on wrong anthropometric diagnosis will be excluded as well. Once a discharge point is reached those children declared as recovered will continue to the post-discharge follow-up for evaluating relapse rates.


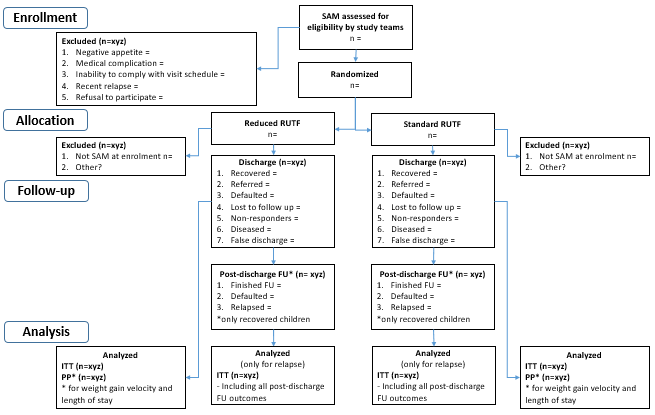


Figure 2: Flow of patients in the MANGO trial

# Descriptive statistics

Descriptive statistics will be reported on the characteristics of both groups included in the intention to treat analysis. Population characteristics (Table 7) will be reported by intervention group as proportions, means (SD), and median (25th- and 75th percentiles) as appropriate, with corresponding group size.

Table 1: Description of baseline characteristics to be reported among the 2 groups

| **Variable** | **Type** | **Units/categories** |
| --- | --- | --- |
| **Age** | continuous | Months |
| **Sex** | binary | %male:%female |
| **Weight** | continuous | Kg |
| **Height** | continuous | Cm |
| **MUAC** | continuous | Mm |
| **WHZ** | continuous | SD |
| **HAZ** | continuous | SD |
| **WAZ** | continuous | SD |
| **Urban (%)** | binary | %urban:%rural |
| **Rural Wealth index (pc1)** | continuous |  |
| **Urban wealth index (pc2)** | Continuous |  |
| **Maternal age** | continuous | years |
| **Maternal education** | binary | % with no formal education |
| **Ethnic group** | categorical | %gourmanché; % mossi; %peul |
| **HFIAS score** | ordinal | % food secure; % mildly food insecure; % moderately food insecure; % severely food insecure |

# General issues in the analysis of intervention effects

Effects are measured by comparing outcomes measured during or after the treatment period or post-discharge follow up between the 2 groups. We will estimate effects when possible; P-values, where reported, will be 2-sided unless otherwise mentioned.

We will describe outcome variables by intervention groups as proportions, means (SD) and median (25th- and 75th percentiles) as appropriate, with corresponding group sizes.

Intervention effects will be assessed as absolute differences (proportions and means) or relative differences in geometric means (variables with lognormal distributions) between groups. To facilitate interpretation, odds ratios resulting from these analyses will be converted to differences in proportions. To allow extrapolation of results to other settings, we will also consider reporting relative effects for binary outcomes (i.e. proportion ratios). Outcomes that are not normally distributed, even after log-transformation, will be compared using non-parametric tests (e.g. Mann-Whitney U test)

# Weight gain velocity

Weight is measured at each visit twice and the mean weight of each participant at each visit will be used to model the mean weight at admission and discharge per group.

Total weight gain velocity will be calculated as:

$$\frac{(\frac{weight at discharge-weight at admission (g)}{weight at admission (kg)})}{length of stay (days)}$$

weight gain velocity after the first 2 two weeks will be calculated as:

$$\frac{(\frac{weight at discharge-weight at visit 2 \left( g \right)}{weight at admission \left( kg \right)})}{length of stay-14 (days)}$$

Missing data on weight at 2^nd^ visit will be estimated as interpolations between available data as

- Weight at visit 1+ 1/2 of the weight gain between visit 1 and visit 3 or
- Weight at visit 1 + 1/3 of the weight gain between visit 1 and visit 4 or
- Weight at admission + 2/3 of weight gain between admission and visit 3.

The difference in weight gain velocity between the two groups will be analysed both for the weight gain from admission to discharge as well as the weight gained after the 1^st^ two weeks of similar treatment among the 2 groups until discharge.

In addition, the weekly evolution of weight gain among the 2 groups will be analysed at each visit and compared. Weekly weight gain will be calculated as mean weight gain of whole group (g) per mean weight at previous visit (kg) per 7 days:

$$\frac{(\frac{mean weight at visit n-mean weight at visit n-1 (g)}{mean weight at visit n-1 (kg)})}{7 (days)}$$

Table 2: Suggested statistical analysis for weight gain velocity

| **End point** | **Primary analysis** | **Extra analysis** |
| --- | --- | --- |
| **Total weight gain velocity from admission to discharge** | **Non-inferiority:**   - Linear mixed effects model of log transformed data* (see below) - 1-sided test (α=0.05) - Both ITT & PP | **(see below)** |
|  | **Difference between control and intervention:**   - Linear mixed effects model on log transformed data* - 2-sided test (α=0.05) - both ITT and PP - Separately for recovered only   *Fixed effects*:   - Intervention   *Random effects:*   - research team - study site | **Test for interaction (only ITT):**  *Potential effect modifiers*   - sex - age category at admission (<12months/≥12months), - MUAC category at admission (<115mm /≥115mm) - WHZ category at admission (<-3 / ≥-3) - Stunting status at admission (HAZ<-2 / HAZ≥-2) - If interaction: sub-group analysis   **Adjusted model:**  *Potential confounders:*   - sex - age at admission - admission weight, - admission MUAC, - admission height, - admission WHZ-score, - month of admission, - wealth score, - length of stay in treatment |
| **Weight gain velocity after 1^st^ 2 weeks** | Same as above | Same as above |
| **Weekly weight gain velocity** | **Difference between control and intervention:**   - Linear mixed effects model on log transformed data - 2-sided test (α=0.05) - ITT   *Fixed effects*:   - Intervention   *Random effects:*   - Research team - Study site - Participant ID | **Adjusted model:**  *Potential confounders:*   - sex - age at admission - admission weight, - admission MUAC, - admission height, - admission WHZ-score, - month of admission, - wealth score, - length of stay in treatment |

# Length of stay

Length of stay will be calculated as days lapsed from the admission to the nutrition program until discharge. Separate analysis will be performed for the main discharge categories of recovered, referred and defaulted as these might have different lengths of stay that influence the ITT analysis: referrals and defaulters might have shorter lengths of stay thus reducing the overall LoS in ITT analysis, giving the wrong impression of a positive outcome.

Table 3: Suggested statistical analysis for length of stay

| **End point** | **Primary analysis** | **Extra analysis** |
| --- | --- | --- |
| **Length of stay** | **Difference between control and intervention:**   - **Linear mixed effects model** - 2-sided test - both ITT and PP (because will inform the possible difference in WGV) - Separately for recovered, referred and defaulted   *Fixed effects*:   - Intervention   *Random effects:*   - research team, - study site | **Test for interaction (only ITT):**  *Potential effect modifiers:*   - sex, - age category at admission (<12months/≥12months), - MUAC category at admission (<115mm /≥115mm) - WHZ category at admission (<-3 / ≥-3) - Stunting status at admission (HAZ<-2 / HAZ≥-2) - If interaction: sub-group analysis   **Adjusted model:**  *Potential confounders:*   - Sex, - age at admission, - admission weight, - admission MUAC, - admission height, - admission WHZ-score, - month of admission, - wealth score, - length of stay in treatment |

# Programmatic outcomes

Outcome of the nutrition program is recorded as one of the following:

1. Recovery
2. Referral to inpatient care
3. Non-response to treatment
4. Defaulter
5. Lost to follow-up
6. Death
7. False discharge

Recovery from SAM is declared based on anthropometric criteria following admission criteria. Thus children admitted with

1. MUAC < 115mm (but WHZ-score ≥ -3) are discharged when their MUAC reaches 125mm or more over two consecutive visits and there is no illness.
2. WHZ-score < -3 (but MUAC ≥ 115mm) are discharged when their WHZ-score reaches -2 or more over two consecutive visits and there is no illness.
3. WHZ-score < -3 and MUAC < 115mm are discharged when their WHZ-score reaches -2 or more AND their MUAC reaches 125mm or more over two consecutive visits and there is no illness.

Both admission and discharge Z-score categories will be declared based on field table readings with 5mm and 100g precisions in length/height and weight measurements respectively.

Children that do not meet the recovery criteria over 16 weeks of treatment are declared non-responders. These include children that only meet the recovery criteria for the 1^st^ time on the 16^th^ visit or who are absent at 16^th^ visit.

Defaulters include children having missed 3 consecutive visits but where a contact has been successfully made to confirm the child alive. Upon contact, the reason for defaulting is enquired and answers categorised into 3:

1. Reasons related to treatment (no need for treatment, the child appears to be healthy)
2. Reasons related to capacity to come to visits (rain, no means of transport, field work, lack of time, moved from the study area)
3. Reasons related to the study (too time consuming, too many procedures, spouse does not support participation, bad reception etc.)

Lost-to-follow up (LTFU) on the contrary, include children having missed 3 consecutive visits but where a contact has not been successful in confirming the child alive. In a sensitivity analysis on the programmatic outcomes, lost-to-follow up will be placed first with the defaulters and then with the diseased to see if this influences the potential differences between the 2 groups.

False discharge include children that 1) were discharged as recovered but upon verification did not meet the anthropometric discharge criteria twice or 2) were discharged referred due to weight loss or stagnant weight but did not meet the definition upon verification.

Table 4: Suggested statistical analysis for programmatic outcomes

| **End point** | **Primary analysis** | **Extra analysis** |
| --- | --- | --- |
| **Programmatic outcomes:**  **- recovery,**  **- referral,**  **- defaulter,**  **- lost to follow up,**  **- non-response,**  **- death,**  **- false discharge** | **Difference between control and intervention**   - Logistical mixed effects regression model - 2-sided test - ITT & PP - Separately for each outcome - Sensitivity analysis with LTFU categorised into defaulters versus diseased   *Fixed effects*:   - Intervention   *Random effects:*   - research team, - study site | **Test for interaction (recovery only):**  *Potential effect modifiers:*   - sex, - age category at admission (<12months/≥12months), - MUAC category at admission (<115mm /≥115mm) - WHZ category at admission (<-3 / ≥-3) - Stunting status at admission (HAZ<-2 / HAZ≥-2) - If interaction: sub-group analysis   **Adjusted model:**  *Potential confounders:*   - sex - age at admission - admission weight, - admission MUAC, - admission height, - admission WHZ-score, - month of admission, - wealth score, - length of stay in treatment |
| **Defaulter reason**  **- treatment related**  **- capability related**  **- study related** | **Difference between control and intervention**   - Chi-square |  |

# Severe adverse events and illnesses

Severe adverse events (SAE) are defined as medical complications requiring hospital admission of patients. Thus they can be considered a sub-category of the referrals to inpatient including all but weight related reasons for referral. The initial categories of referrals to inpatient care are:

1. Development of edema
2. Severe Acute Respiratory Infection (ARI)
3. Severe malaria
4. Diarrhea with dehydration
5. Persistent diarrhea
6. Dysentery (diarrhea with blood)
7. Fever> 39 °C or hypothermia <35 °C
8. Repeated or constant vomiting
9. Severe anemia (Hb level of <4 g / dl)
10. Abscess or extensive skin lesions
11. Very weak, unconscious or apathetic
12. Convulsions or discomfort
13. Negative appetite*
14. Weight loss for two (2) consecutive visits
15. Weight stagnant for three (3) consecutive visits

**In practice, after admission, a negative appetite alone was judged not sufficient for referral and only led to effective referral when accompanied by another severe illness as defined above*

Thus for the sake of the SAE analysis the referrals will be further categorised to:

1. Medical complications (including initial referral categories 1-12)
2. Weight loss defined as loss of ≥ 5% weight over 3 weeks without apparent illness explaining this (as per initial referral category 14)
3. Stagnant weight gains defined as no more than 100g weight gain over 4 weeks without apparent illness explaining this (as per initial referral category 15)

Illnesses are recorded at each visit as reported by the caregiver concerning any days with illness during the past week. Both the type of illness as well as the estimated number of days that the child has suffered from the illness are recorded. Thus both the frequency (at how many visits is there an illness reported for the past week) and the total number of days with illness during the treatment can be estimated and compared. Only illnesses reported after admission from first treatment visit onwards will be accounted for.

Table 5: Suggested statistical analysis for severe adverse events & illnesses

| **End point** | **Primary analysis** | **Extra analysis** |
| --- | --- | --- |
| **Severe adverse events**  **(i.e. referrals for medical complications)** | **Difference between control and intervention**   - Chi-square test (or no tests if too few observations) - 2-sided test - ITT - Separately for each main referral category (medical complication, weight loss and stagnant weight) - If difference in total number 🡪 separately for each medical complication | **Adjusted model:**  *Potential confounders:*   - sex - age at admission - admission weight, - admission MUAC, - admission height, - admission WHZ-score, - month of admission, - wealth score, - length of stay in treatment |
| **Frequency of illnesses**  (number of visits upon which an illness is reported during the past week) | **Difference between control and intervention**   - Linear mixed effects regression model - 2-sided test - ITT   *Fixed effects*:   - Intervention   *Random effects:*   - research team, - study site | If difference found: analysis per illness |
| **Days with illness**  (total number) | **Difference between control and intervention**   - Linear mixed effects regression model - 2-sided test - ITT   *Fixed effects*:   - Intervention   *Random effects:*   - research team, - study site | If difference found: analysis per illness |

# Linear and MUAC growth velocity

Height/length and MUAC measurements are recorded at each visit twice and their mean will be used to calculate the growth velocity during the study from admission to discharge regardless of exit category. Weekly linear and MUAC gain velocity will also be modelled per visit and per group.

If a child switches from recumbent length measurement to standing height measurement during the study, 7 mm will be added to the exit height to standardise start and end measurements to the same scale of recumbent length. Alternatively, if a child switches from standing height to recumbent length, 7 mm will be distracted from the exit measure.

Table 6: Suggested statistical analysis for linear and MUAC growth velocity

| **End point** | **Primary analysis** | **Extra analysis** |
| --- | --- | --- |
| **Linear growth velocity during treatment** | Difference between control and intervention:   - **Linear mixed effects model** (on log transformed data if data is not normally distributed) - 2-sided test - ITT & PP   *Fixed effects*:   - Intervention   *Random effects:*   - research team, - study site | Test for interaction:  *Potential effect modifiers:*   - sex, - age category at admission (<12months/≥12months), - MUAC category at admission (<115mm /≥115mm) - WHZ category at admission (<-3 / ≥-3) - Stunting status at admission (HAZ<-2 / HAZ≥-2) - If interaction: sub-group analysis   **Adjusted model:**  *Potential confounders:*   - sex - age at admission - admission weight, - admission MUAC, - admission height, - admission WHZ-score, - month of admission, - wealth score, - length of stay in treatment |
| **Weekly linear growth velocity during treatment** | Difference between control and intervention:   - **Linear mixed effects model** (on log transformed data if data is not normally distributed) - 2-sided test - ITT   *Fixed effects*:   - Intervention   *Random effects:*   - research team, - study site - Participant ID | **Adjusted model:**  *Potential confounders:*   - sex - age at admission - admission weight, - admission MUAC, - admission height, - admission WHZ-score, - month of admission, - wealth score, - length of stay in treatment |
| **MUAC growth velocity during treatment** | Difference between control and intervention:   - **Linear mixed effects model** (on log transformed data if data is not normally distributed) - 2-sided test - ITT & PP   *Fixed effects*:   - Intervention   *Random effects:*   - research team, - study site | **Adjusted model:**  *Potential confounders:*   - sex - age at admission - admission weight, - admission MUAC, - admission height, - admission WHZ-score, - month of admission, - wealth score, - length of stay in treatment |
| **Weekly MUAC growth velocity during treatment** | Difference between control and intervention:   - **Linear mixed effects model** (on log transformed data if data is not normally distributed) - 2-sided test - ITT   *Fixed effects*:   - Intervention   *Random effects:*   - Research team - Study site - Participant ID | **Adjusted model:**  *Potential confounders:*   - sex - age at admission - admission weight, - admission MUAC, - admission height, - admission WHZ-score, - month of admission, - wealth score, - length of stay in treatment |

# Relapse rate

Relapse rate will be calculated over the number of children discharged recovered from the initial SAM treatment and who then continued to a 3 month long follow up. Four possible end points are noted for these children:

1. Finished the 3-month follow up without relapsing
2. Defaulted during the 3-month follow up
3. Absent only at the last follow up visit
4. Relapsed to SAM during the 3-month follow up period

Relapse to SAM is defined as presenting a WHZ<-3, a MUAC<115mm or any grade of bilateral oedema.

Table 7: Suggested statistical analysis for relapse rate

| **End point** | **Primary analysis** | **Extra analysis** |
| --- | --- | --- |
| **Relapse to SAM** | **Difference between control and intervention:**   - **Logistic mixed effects model** - 2-sided test - ITT   *Fixed effects*:   - Intervention   *Random effects:*   - Research team - Study site | **Adjusted model:**  *Potential confounders:*   - sex - age at admission - admission weight, - admission MUAC, - admission height, - admission WHZ-score, - month of admission, - wealth score, - length of stay in treatment |
