# Supplementary material for: Impact of reduced dose of ready-to-use therapeutic foods in children with uncomplicated severe acute malnutrition: A randomised non-inferiority trial in Burkina Faso
Source: PLoS Med. 2019 Aug 27;16(8):e1002887. doi: 10.1371/journal.pmed.1002887 (PMC6711495; doi:10.1371/journal.pmed.1002887)
Supplement: S1 Abstract — (DOCX) [file pmed.1002887.s007.docx]

# Abstract

## Contexte

Les enfants Malnutris Aigus Sévères (MAS) sont traités à domicile à l’aide des Aliments Thérapeutiques Prêts à l’Emploi (ATPE). A présent, la quantité d’ATPE prescrite est déterminée selon le poids de l’enfant afin de couvrir 100% de ses besoins jusqu’à la sortie du traitement. Néanmoins, la dose est questionnée car elle semble être partagée donnant lieu à un cout-efficacité moindre du traitement de la MAS. Nous avons investigué l’efficacité d’une dose réduite d’ATPE dans la prise en charge communautaire de la MAS.

## Méthodes et Résultats

Nous avons mené un essai randomisé testant la non-infériorité de la vélocité de gain de poids chez les enfants MAS recevant a) une dose standard d’ATPE pendant deux semaines suivie d’une réduction de la dose (dose réduite), comparée à b) une dose standard d’ATPE durant tout le traitement (dose standard). Une différence moyenne de 0.0 g/kg/j était attendue avec une marge de non-infériorité fixée à -0.5 g/kg/j. Des modèles mixtes linéaires et logistiques ont été réalisés avec le site d’étude et l’équipe comme effets aléatoires.

Entre octobre 2016 et juillet 2018, 801 enfants avec MAS sans complication âgés de 6-59 mois ont été admis dans 10 centres de santé communautaires au Burkina Faso. A l’admission, l’âge moyen (±ET) était de 13.4 mois (±8.7), 49% étaient de sexe masculin et le poids moyen était de 6.2 kg (±1.3). La vélocité de gain de poids moyenne était de 3.4 g/kg/j et n’était pas différente entre les bras de l’étude (Δ 0.0 g/kg/j; CI 95% -0.4, 0.4; p=0.92) confirmant la non-infériorité (p=0.013). Cependant, après deux semaines la vélocité de gain de poids était significativement plus faible avec la dose réduite avec une moyenne de 2.3 g/kg/j comparée à 2.7 g/kg/j avec la dose standard (Δ -0.4 g/kg/d; CI 95% -0.8, -0.02; p=0.041).

La durée de séjour n’était pas différente (p=0.73) entre les groupes avec une médiane de 56 jours (EI 35, 91) dans les deux bras. Il n’y avait pas de différence entre la dose réduite et la dose standard en termes de taux de guérison (52.7% et 55.4%; p=0.45), taux de référencement (19.2% et 20.1%; p=0.80), taux d’abandon (12.2% et 8.5%; p=0.088), taux de non-réponse (12.7% et 12.5%; p=0.95), et rechute (2.4% et 1.8%; p=0.69), respectivement. Néanmoins, la dose réduite avait un petit effet négatif de 0.2 mm/s (IC 95% 0.04, 0.4; p=0.015) sur la vélocité de gain de taille avec une moyenne de 2.6 mm/s dans la dose réduite et 2.8 mm/s dans la dose standard. L’impact était plus prononcé chez les enfants âgés de <12 mois (interaction, p=0.019) qui gagnaient 2.8 mm/s avec la dose réduite et 3.1 mm/s avec la dose standard (Δ -0.4 mm/w; IC 95% -0.6, -0.2; p<0.001).

Les limites incluent le fait que les participants n’étaient pas en aveugle vis-à-vis de la dose d’ATPE reçue, et l’exclusion de tous les enfants avec un test d’appétit négatif. Les résultats sont généralisables à un contexte de sécurité alimentaire et a des populations de MAS jeunes.

## Conclusions

La réduction de la dose d’ATPE chez les enfants MAS après 2 semaines de traitement n’a pas réduit la vélocité de gain de poids ou de PB ni impacté la guérison ou prolongé la durée de traitement. Cependant, elle a eu un petit effet négatif significatif sur la croissance linéaire, surtout parmi les plus jeunes. Les effets potentiels de la réduction de la dose d’ATPE dans un programme de routine sur les résultats du traitement devraient être investigués avant une mise à l’échelle.

## Registration de l’essai

Registre ISRCTN ISRCTN50039021
